# Stable Intronic Sequences and Exon Skipping Events in the Human RPE65 Gene: Analysis of Expression in Retinal Pigment Epithelium Cells and Cell Culture Models

**DOI:** 10.3389/fgene.2019.00634

**Published:** 2019-07-19

**Authors:** Olga Postnikova, Eugenia Poliakov, Nady Golestaneh, Igor B. Rogozin, T. Michael Redmond

**Affiliations:** ^1^Laboratory of Retinal Cell & Molecular Biology, National Eye Institute, NIH, Bethesda, MD, United States; ^2^Departments of Ophthalmology, Neurology, Biochemistry and Molecular & Cellular Biology, Georgetown University Medical Center, Washington, DC, United States; ^3^National Center for Biotechnology Information, National Library of Medicine, NIH, Bethesda, MD, United States

**Keywords:** stable intronic sequence RNA, long non-coding RNA, RNA-seq, visual cycle, retinal dystrophy

## Abstract

Currently, there is much interest in intronic sequence-containing long non-coding RNAs and the role of intronic transcription in regulation of cellular metabolism and fate. Several stable intronic sequence RNAs (sisRNAs) were recently implicated in regulation of parental genes. To investigate transcription from introns of the RPE65 gene, we analyzed RNA-seq and Nanopore sequencing data from different cell models of human retinal pigment epithelium (RPE) and native bovine RPE. We discovered putative stable poly-adenylated transcripts with sequences corresponding to intronic regions of the RPE65 gene in the cytoplasm of RPE cells. These stable intronic sequences could be important for RPE65 transcription, splicing or translation. We also analyzed alternative splicing events in RPE65. Frequent exon skipping events involving exons 2, 3, and 7 were detected. The rate of these events was much higher in human RPE cell cultures compared with native RPE , consistent with lack of translation of RPE65 mRNA in cell cultures.

## Introduction

Light activation of visual pigments in the mammalian retina leads to the phototransduction pathway and concurrent release of the photoisomerized chromophore. Mammalian vision depends on constant recycling of 11-*cis* retinal chromophore in the multistep visual (retinoid) cycle in the retinal pigment epithelium (RPE), a monolayer epithelium adjacent to the photoreceptor outer segments. RPE65 is the central isomerohydrolase in the RPE visual cycle that catalyzes the conversion of all-*trans*-retinyl ester (atRE) into 11-*cis* retinol (Jin et al., 2005; Moiseyev et al., 2005; Redmond et al., 2005). RPE65 is one of three mammalian members of the carotenoid oxygenase family and has evolved to become a retinol isomerohydrolase in the vertebrate retinal visual cycle (Poliakov et al., 2012). RPE65 is highly expressed in RPE. Human RPE65 mutations cause a spectrum of retinal dystrophies that result in blindness (RP20, LCA2) (Marlhens et al., 1997; Morimura et al., 1998). Except for one recently described mutation D477G (Bowne et al., 2011), RPE65 mutations have been invariably recessively inherited (Thompson et al., 2000).

The RPE carries out several important functions including light absorption, epithelial transport, visual cycle, phagocytosis, secretion, and immune modulation (Strauss, 2005). It is extremely difficult to reproduce all RPE functions in cell culture models, and all of the currently existing RPE cell culture models carry out only some of the functions of native RPE (Boulton, 2014; Lehmann et al., 2014; Rizzolo, 2014). The human RPE cell line ARPE-19 provides a widely used alternative to native RPE. However, replication of the native RPE phenotype becomes increasingly difficult as these cells lose their specialized phenotype after multiple passages. Compounding this problem is the widespread use of ARPE-19 cells in an undifferentiated state to allow for transfection and for shorter timeframe experiments. mRNAs of RPE-expressed genes, including RPE65, RDH5, and RDH10, as well as miR-204/211, were greatly increased in ARPE-19 cells maintained at confluence for 4 months (Samuel et al., 2017). However, ARPE-19 does not express RPE65 protein. RPE65 mRNA and protein are both abundant in native RPE, while explanted bovine RPE cells lose RPE65 protein expression after 2 weeks in culture but retain RPE65 mRNA (Hamel et al., 1993). It was proposed that the 3′-untranslated region (UTR) of the mRNA might contain elements key to translational regulation (Liu and Redmond, 1998). However, the question as to why RPE65 protein is not expressed in cell culture is still not resolved. This issue may be related to the accuracy of RPE65 pre-mRNA splicing. Recently, a c.1430A > G (p.(D477G)) mutation in RPE was reported to cause a dominantly inherited retinal degeneration (Bowne et al., 2011). We found that instead of merely producing a missense mutant protein, the A > G nucleotide substitution generated an ectopic splice site that disrupts RPE65 splicing and protein expression in a mouse model of the c.1430A > G mutation (Li et al., 2019) and that was replicated in an *in vitro* exontrap assay of human RPE65 gene constructs. This was correlated with the relatively weak canonical splice sites for exons 12 and 13 close to the mutation.

Thus, accuracy of splicing may be an important factor in RPE65 expression. Taking into account that no functional alternatively spliced RPE65 isoforms have been detected in vertebrates, rare exon skipping events can produce non-functional mRNAs (Li et al., 2019). However, the extent of human RPE65 splicing defects remains unclear. Disturbed alternative splicing may be one possible explanation of the observed dominant inheritance of the pathogenic c.1430A > G human RPE65 mutation (Bowne et al., 2011; Li et al., 2019). Exon skipping is known for human the RPE65 gene; specifically, rare exon skipping events were detected for exons 2, 3, and 7 (Ryan et al., 2016). It is also known that long non-coding RNAs (lncRNAs) have major impact on splicing accuracy of some human genes (Romero-Barrios et al., 2018). Several lncRNAs have been linked to the modulation of alternative splicing in eukaryotes (Romero-Barrios et al., 2018). The main mechanisms involving lncRNAs in alternative splicing regulation can be classified in three different ways: 1) lncRNAs interacting with specific splicing factors; 2) lncRNAs forming RNA–RNA duplexes with pre-mRNA molecules; and 3) lncRNAs affecting chromatin remodeling, thus fine-tuning the accuracy of splicing of target genes (Romero-Barrios et al., 2018). However, alternative splicing of RPE65 pre-mRNA and lncRNAs associated with the RPE65 gene has never been studied systematically.

We conducted a comparison of RPE65 expression profiles in native and cell-based models of RPE. Significant differences in exon skipping events were detected for exons 2, 3, and 7. We also found a putative stable intronic sequence RNA (sisRNA) that encompasses the intron 9, the exon 10, and the intron 10. Analyses of RNA-seq and nanopore long-read sequencing data suggest that this sisRNA was moderately expressed in human RPE and ARPE-19 cell culture and is likely to be polyadenylated. We also predict the existence of another sisRNA in intron 13. The presence of both sisRNA-RPE65 transcripts was confirmed experimentally in ARPE-19 cell culture. Both sisRNAs were detected in native bovine RPE samples by RNA-seq and PCR experiments.

## Methods

### Data Sets and Cell Cultures

RNA-seq data used in this study are described in **Table 1**. Cultured human ARPE-19 cells were grown and differentiated as previously described (Samuel et al., 2017). Native RPE cells were harvested from bovine eyes as previously described by Postnikova et al. (2019). Primary human RPE cells were isolated from AMD patients and age-matched controls post-mortem and cultured for 4 weeks according to Golestaneh et al. (2017).

**Table 1:** RNA-seq data sets.

Data set/total reads	Reads/library	Description	Publication
Human retinal pigment epithelium (RPE)/choroid 17.7 million	2 × 100 bp Paired-end stranded	Retina and RPE/choroid from the temporal, macular, and nasal regions of four clinically normal human donor eyes	Whitmore et al. (2014)
Human ARPE-19 cells/87.3 million	2 × 50 bp Paired end rRNA depleted	ARPE-19 cells cultured for 4 days or 4 months	Samuel et al. (2017)
Bovine RPE cells/13.3 million	2 × 50 bp Paired end rRNA depleted	Fresh native bovine RPE; bovine RPE cultured for 4 and 8 weeks	Postnikova et al. (2019)
AMD/controls, primary human RPE cells/23.6 million	2 × 100 bp Paired-end stranded	Primary RPE from AMD donors and controls cultured for 4 weeks	Golestaneh et al. (2017)

### Sequence Analysis

Reads from native and cultured human RPE were aligned with Star 2.5 (https://github.com/alexdobin/STAR) and Cufflinks (http://cole-trapnell-lab.github.io/cufflinks/cuffdiff/) to the hg38 reference human genome. The dbEST database of National Center for Biotechnology Information (http://ncbi.nlm.nih.gov/) was searched using the BLASTN program with default parameters. Mobile elements were masked using the RepeatMasker program (http://www.repeatmasker.org/cgi-bin/WEBRepeatMasker) with default parameters. The UCSC genome browser (https://genome.ucsc.edu/cgi-bin/hgBlat) was used for analysis of evolutionary conservation of the RPE65 gene. The Fisher exact test was used to compare distributions of exon-skipping events across the RPE65 gene; calculations were done using the COLLAPSE program (Khromov-Borisov et al., 1999). We analyzed the correlation between raw numbers of reads of the exon 8 and intronic parts of the “AW205227.1” sisRNA according to the bb840fee-400c-4858-9439-fb612ed78dfe read (**Supplementary Table S1**), using the Pearson’s linear correlation coefficient. We also analyzed the correlation between intronic parts of the “AW205227.1” sisRNA and the raw number of exon 7 skipping events. The CLUSTERM program (Glazko et al., 1998; Kondrashov and Rogozin, 2004) was used for analysis of exon-skipping events in the 4-month-old ARPE-19 cell culture (4M_ARPE-19) data set. We assume that deviation from a standard binomial distribution reflects significantly elevated frequencies of certain exon-skipping events.

### Experimental Procedures

RNA was extracted from bovine native RPE and ARPE-19 cultured human RPE cells using Promega Maxwell RSC simplyRNA automated system, according to the manufacturer’s protocol. To obtain cytoplasmic and nucleus fractions, fractionation buffers from the PARIS kit (Ambion) were used according to the manufacturer’s protocol. cDNAs were prepared using oligo dT primer and random hexamers (High-Capacity cDNA Reverse Transcription Kit, Applied Biosystems). For all samples no-RT controls were generated and used for polymerase chain reaction (PCR).

For rapid amplification of cDNA ends (RACE) (Clontech) and nanopore sequencing isolation of polyA highly-purified intact RNA was achieved with Dynabeads Oligo(dT)25 (ThermoFisher Scientific) according to the manufacturer’s protocol. Primers are listed in the **Supplementary Table S2**.

Splice isoforms of RPE65 were examined by PCR amplification from ARPE-19 cDNA with RPE65 primers in 5′ and 3′ UTR regions (forward primer: 5′-caaagcaaccggtgatatcgtccttcttcattctgcag-3′ and reverse primer: 5′-cagtacaaagtgttacccctatttttgtcaatatttatttttaaacaatc-3′). Products were directly cloned into pv2 plasmid prepared with BamH1 and Xba1 restriction enzymes.

TaqMan primers for qPCR for RPE65 isoform without exon 7 were Probe 5′-/56FAM/ccactgcaa/zen/gcagttttggt/31ABkFQ/-3′; Primer 1 5′cccaaagactccatgaagaaag3′; Primer 2 5′gcctacaacattgtaaagatccc3′. Relative expression was calculated using delta Ct method relative to GAPDH (IDT).

Direct cDNA sequencing kits (SQK-DCS109, Oxford Nanopore Technologies) were used to prepare libraries for nanopore sequencing using the FLO-MINSP6 flow cell (Oxford Nanopore Technologies). For data analysis the Fastq Human Exome workflow from EPI2ME was used to identify reads that were aligned to RPE65 gene. After base calling, the reads from the sample are aligned to a set of reference sequences from Ensembl using the minimap2 aligner, covering the entire human exome. Alignments are performed against full gene sequences, including exons and introns. The reference sequences are taken from the feature strand of the GRCh38.

## Results

### Analysis of Human RPE65 Splicing Accuracy Using Nanopore Long-Read Sequencing and RNA-seq Data

We performed nanopore sequencing of cDNA from 4-month-old differentiated ARPE19 (4M_ARPE-19) cells. Thirty significant hits to the RPE65 gene were detected using a conventional nanopore pipeline (**Supplementary Table S1** and **Supplementary Data File** see Methods for details). We found several complete or nearly complete RPE65 mRNA sequences and many incomplete 3′UTR regions (**Supplementary Table S1**). In addition, we detected several reads that encompass various introns of the RPE65 gene (**Supplementary Table S1**).

**Figure 1:**
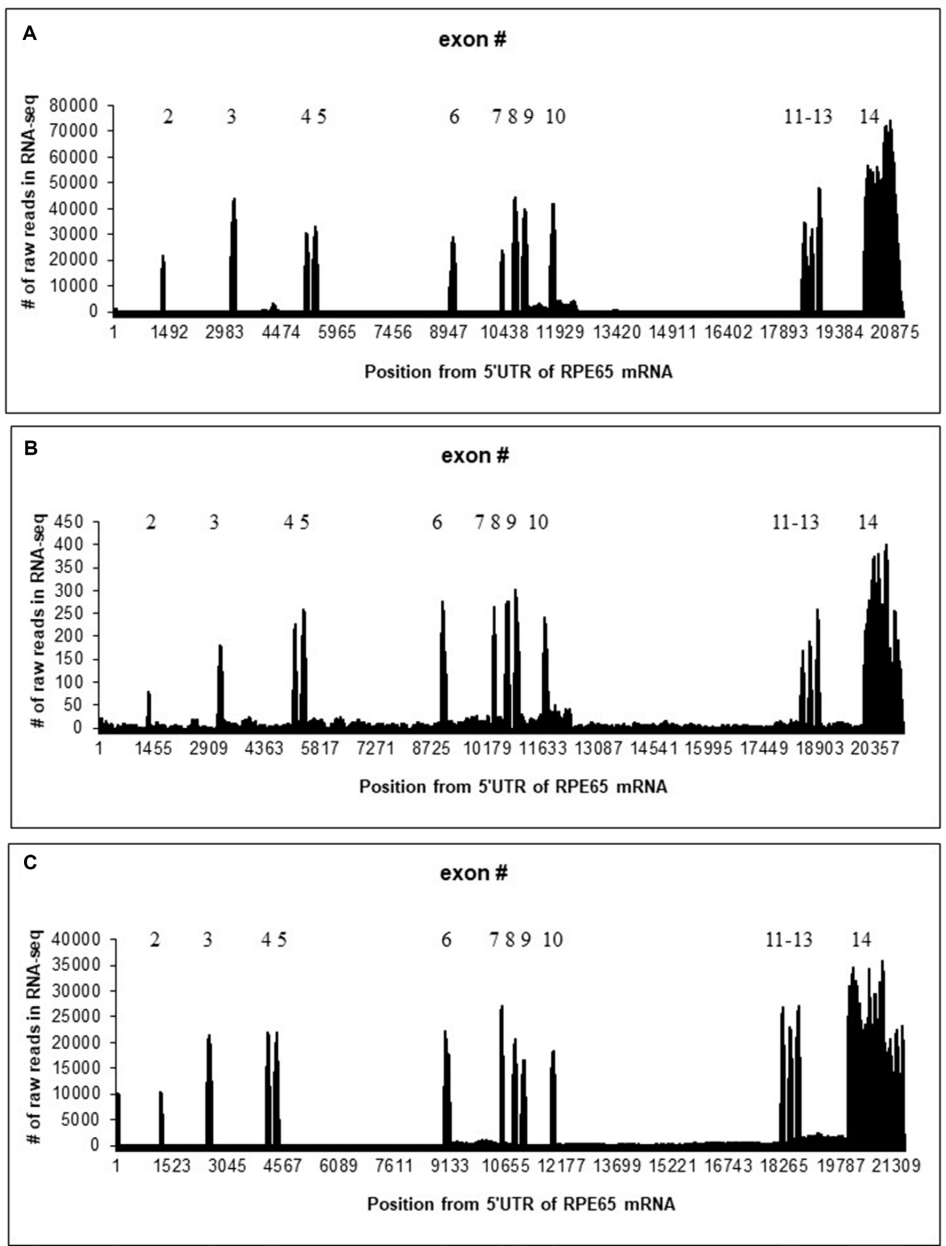
Distribution of the number of raw reads in each position of human/bovine RPE65 mRNA sequences (Y axis) mapped on the human/bovine genomic sequences (X axis). Position 1 corresponds to the first position of RPE65 mRNA (NM_000329.2). **(A)** Merged RNA-seq data for native human RPE/choroid. **(B)** Merged RNA-seq data for human 4M_ARPE-19 cell line. **(C)** Merged RNA-seq data for native bovine RPE (**Table 1**).

Mapping of RNA-seq reads to the RPE65 gene suggested that 4M_ARPE-19 and human RPE/choroid (Whitmore et al., 2014) samples are similar although the level of expression is much lower in 4M_ARPE-19: the number of raw reads in each position is roughly 100 times lower with approximately similar sizes of RNA-seq data sets (**Figure 1** and **Table 1**). Some noticeable differences of expression profiles in the 3′UTR (exon 14) may be due to differences in RNA-seq procedures.

Our analysis of splicing junctions revealed rare cases of exon-skipping events in native human RPE/choroid samples: exons 2, 3, 4, 7, and 12 have been found to be skipped with a low frequency (<5% of total amount of reads containing RPE65 exon junctions) (**Table 2**). The exon-skipping events are likely to be much more frequent in 4M_ARPE-19 cells (**Table 2**). Specifically, for exons 2, 3, and 7 significantly higher frequencies of skipping events were detected (**Table 2**). Skipping of exons 4 and 12 were not significantly different between native human RPE/choroid samples and 4M_ARPE-19 samples (**Table 2**). Analysis of exon-skipping events in 4M_ARPE-19 using the CLUSTERM program (Glazko et al., 1998; Kondrashov and Rogozin, 2004) delineates two classes based on the number of skipping events (corresponding to two binomial distributions), one class contains 0 to 2 exon skipping events, the second class contains 8 to 13 exon skipping events. This result suggested that exon skipping events of exons 2, 3, and 7 have much higher frequency compared to other exons in 4M_ARPE-19 samples (**Table 2**).

**Table 2:** Frequency of RPE65 exon-skipping events estimated using RNA-seq data sets.

Exon pairs (correct and incorrect splicing events)	RNA-seq library	Raw number of correct exon splicing events	Raw number of incorrect exon splicing events	Fraction of incorrect splicing events (*P* value)
Exons 1–2 and 1–3	Human RPE	15,898	137	0.009
Exons 1–2 and 1–3	ARPE-19 (4 months)	198	8	0.040 (0.0005)
Exons 2–3 and 2–4	Human RPE	23,407	233	0.010
Exons 2–3 and 2–4	ARPE-19 (4 months)	189	11	0.055 (0.00001)
Exons 3–4 and 3–5	Human RPE	27,555	112	0.004
Exons 3–4 and 3–5	ARPE-19 (4 months)	406	2	0.005 (0.68)
Exons 4–5 and 4–6	Human RPE	19,375	0	0
Exons 4–5 and 4–6	ARPE-19 (4 months)	227	0	0
Exons 5–6 and 5–7	Human RPE	26,418	0	0
Exons 5–6 and 5–7	ARPE-19 (4 months)	169	0	0
Exons 6–7 and 6–8	Human RPE	31,017	937	0.030
Exons 6–7 and 6–8	ARPE-19 (4 months)	178	13	0.073 (0.0046)
Exons 7–8 and 7–9	Human RPE	25,913	0	0
Exons 7–8 and 7–9	ARPE-19 (4 months)	122	0	0
Exons 8–9 and 8–10	Human RPE	23,428	0	0
Exons 8–9 and 8–10	ARPE-19 (4 months)	199	0	0
Exons 9–10 and 9–11	Human RPE	20,947	0	0
Exons 9–10 and 9–11	ARPE-19 (4 months)	201	0	0
Exons 10–11 and 10–12	Human RPE	21,608	0	0
Exons 10–11 and 10–12	ARPE-19 (4 months)	191	0	0
Exons 11–12 and 11–13	Human RPE	24,773	8	0.0003
Exons 11–12 and 11–13	ARPE-19 (4 months)	208	0	0 (1.00)
Exons 12–13 and 12–14	Human RPE	27,011	0	0
Exons 12–13 and 12–14	ARPE-19 (4 months)	197	0	0

P values (in parentheses, underlined) were estimated for comparisons “Human retinal pigment epithelium (RPE) vs. 4M_ARPE-19 cells” and “correctly spliced introns vs. incorrectly spliced introns” (2 × 2 contingency tables) using the Fisher exact test. To take into account variations in read lengths (**Table 1**), only the last 50 nucleotides and the first 50 nucleotides from each analyzed pair of exons around splicing junctions were used for analysis.

Experimentally, we were able to confirm the presence of RPE65 transcripts in differentiated 4M_ARPE-19 cells and in native bovine RPE (5′ RACE; with PCR; see Experimental Procedures) that lack exon 7 (**Supplementary Figure S1**). This mis-spliced transcript is much less abundant (∼4-fold less) according to qPCR data compared to normally spliced RPE65 mRNA in differentiated 4M_ARPE-19 cells (**Supplementary Figure S2**). We noticed that RPE65 mRNA amplified with exons 10 to 11 read-through primer is more abundant in cytoplasm compared to exon 13–14 read-through primer amplified mRNA (**Supplementary Figure S3** and **Table S3**). The nuclear retention for exons 13 to 14 read-through primer-amplified mRNA could be explained by genomic DNA contamination because of the short intron 13 (Ct = 30.5).

Otherwise, splicing of RPE65 pre-mRNAs was accurate for 4M_ARPE-19 and native human and bovine RPE samples where many RNA-seq mapped reads were observed (**Figure 1**). However, we detected a substantial fraction of reads in introns. Specifically, introns 9 and 10 were particularly enriched in paired reads (**Figure 1**). These results are consistent with several nanopore reads that encompass RPE65 introns (**Supplementary Table S1**). This may indicate the presence of stable non-coding RNAs within the RPE65 gene. The number of reads in the 4-day-old ARPE-19 cell culture (4D_ARPE-19) and AMD+Controls merged primary cell culture (Golestaneh et al., 2017) is very low (**Supplementary Figure S4**). All mapped reads are located within exons except one read in AMD+Controls (around the position 17,001, **Supplementary Figure S4**). This result is largely compatible with the hypothesis that the expression of putative stable non-coding RNAs within the RPE65 gene correlate with the expression of RPE65 mRNA.

### Analysis of Human Introns Using Nanopore and RNA-seq Data: Novel sisRNA Genes

**Figure 2:**
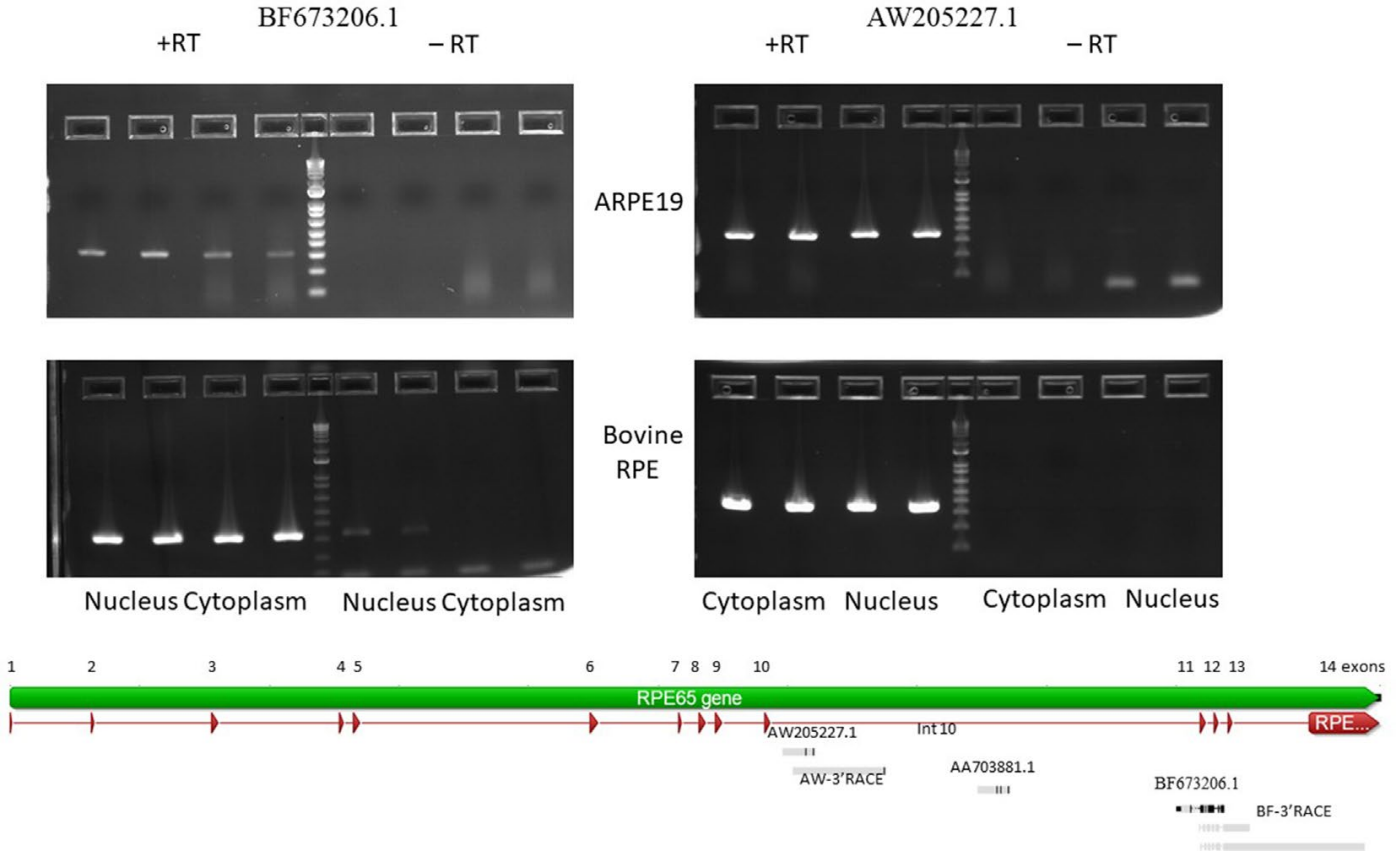
Agarose gel electropherogram of PCR products of BF673206.1 and AW205227.1 EST from cDNA and no RT control from 4M_ARPE-19 and native bovine RPE cells. Graphical representation of alignments of the EST and RACE products on to RPE65 gene (Geneious 9.1.5).

BLASTN searches of RPE65 introns against dbEST revealed three EST sequences homologous to RPE65 introns. One EST (AW205227.1) overlaps with the beginning of the intron 10 (**Supplementary Figure S5**). This EST seems to be polyadenylated with the canonical polyA signal AATAAA near the 3′ end (**Figure 2**); this EST has 99.8% identity with the intron 10, excluding the polyA sequence at the 3′ end. This EST corresponds to a region on chr1 68437577 to 68438036 that is enriched in mapped reads (**Figure 1**). We hypothesize that this is a fragment of a novel polyadenylated lncRNA. According to RNA-seq data, this sisRNA may be longer, encompassing exon 10 and intron 9 (**Figure 1**).

BF673206.1, another EST (**Figure 2**), overlaps with intron 10 (partially), exon 11, intron 11, and exon 12 (partially). It has 95.8% identity with chr1 68431350 to 68431958 and spans 609 bp. This EST has many mismatches with the genomic sequences (∼4%). This may be due to low quality of cloning/sequencing procedures. However, the number of raw reads in the intron 10 to exon 12 region is not high compared to the AW205227.1 sequence.

We hypothesize that both ESTs correspond to unknown putative sisRNAs. To test this hypothesis, we analyzed the distribution of reads in intronic sequences. We mapped paired reads only using the rule that one read should have an overlap (>10 nucleotides) with an intron, another read can have any location. The results of this analysis (**Supplementary Figures S6** and S7) suggest that the putative “AW205227.1” sisRNA is much more abundant (∼10 times) compared to the putative “BF673206.1” sisRNA. The analysis did not suggest any putative intronic sequences in sisRNAs (**Supplementary Figures S6** and S7) and some variations in the distribution of reads is likely to be the result of the imposed rules (at least one read should overlap with an intron).

Nanopore sequencing of 4M_ARPE-19 cells confirmed sisRNAs “AW205227.1” and “BF673206.1”, with a higher frequency of the “AW205227.1” sisRNA (**Supplementary Table S1**). However, the exact structure of either sisRNA remains elusive. There are two versions of the “AW205227.1” sisRNA: one nanopore read covers intron 9 (partially), exon 10 and intron 10 (partially) (bb840fee-400c-4858-9439-fb612ed78dfe, **Supplementary Figure S8** and **Supplementary Table S1**) while another read (33cd27e9-8975-4acd-adce-f033b9f9324b) is much longer, covering several exons in addition to intronic sequences (**Supplementary Table S1**). One partial “BF673206.1” sisRNA read was also recovered by the nanopore sequencing technique (c5597c60-f0ef-40e5-bd45-c0acf29eb582, **Supplementary Table S1**).

Correlation between the expression of non-coding RNAs and mRNAs is a powerful tool to find putative functional roles for non-coding RNAs (Fernandes et al., 2019; Ulitsky and Bartel, 2013). We analyzed a correlation between the raw number of reads of exon 8 and intronic parts of the “AW205227.1” sisRNA. Significant positive correlation was detected for native RPE samples and 4M_ARPE-19 samples (CC = 0.90 and 0.87) (**Supplementary Figure S9**). We also analyzed a correlation between intronic parts of the “AW205227.1” sisRNA and the number of exon 7 skipping events across native RPE samples and 4M_ARPE-19 samples (**Supplementary Figure S10**). No significant correlation was found for native RPE samples (CC = 0.1, P = 0.37, **Supplementary Figure S10A**), although a significant positive correlation was detected for 4M_ARPE-19 samples (CC = 0.5, P = 0.03, **Supplementary Figure S10B**), despite a much smaller number of exon skipping events (**Table 2**).

### Additional Experimental Verification of *in silico* Predictions

To confirm expression of the BF673206.1 and AW205227.1 ESTs that overlap with intronic sequences of the RPE65 gene, we performed PCR with primers located in introns of the RPE65 gene (**Supplementary Table S2**). PCR products corresponding to AW205227.1 and BF673206.1 sequences were clearly present in the cytoplasm and nucleus fractions of RPE from bovine eyes and the human differentiated cell line ARPE-19 (**Figure 2**). The absence of PCR product in no-RT controls (except the nucleus fraction for BF673206.1 EST; **Figure 2**) indicated that these transcripts indeed are stable in cytoplasm of native bovine RPE and ARPE-19 cells and that this result is not due to genomic contamination.

To find the transcription start and end sites for these ESTs we employed the RACE technique. In the human ARPE-19 line we were only able to obtain 3′ RACE clones for both ESTs. This might be due to the 10 to 100 times reduced expression of RPE65 mRNA in ARPE-19 cell line versus native RPE. Another possible explanation is the presence of secondary structure at the 5′ end of these RNAs. We recovered a 1,393 bp 3′ RACE polyadenylated product from the nested primer that ended in intron 10 for AW205227.1 EST. A similar 3′ RACE polyadenylated product was recovered from native bovine RPE (**Figure 2**). This product is longer than the putative “AW205227.1” sisRNA (**Supplementary Figure S8**). It is not easy to reconcile results of the 3′RACE with EST, RNA-seq and nanopore sequencing results. It is possible that 3′RACE recovered some rare longer isoform that we cannot detect using other methods.

Starting from the nested primer, two 3′RACE products for the BF673206.1 EST, 1534 bp and 742 bp long, were identified. These transcripts overlap with exons 11, 12, 13 and introns 11, 12, and 13 of the RPE65 gene, with two different polyadenylation signals in intron 13. Also, we recovered a transcript that overlaps with exons 11-14 and introns 11-13 (**Figure 2**). It is possible this could be an incompletely spliced (carrying at least part of intron 10 and full intron 11) polyadenylated transcript from RPE65 gene.

### Comparative Analysis of sisRNA-RPE65 in Mammalian Genomes

The analysis of evolutionary conservation of the sisRNA regions using the UCSC genome browser did not reveal any obvious islands of conservation in intronic sequences. For example, the polyA signal (AATAAA, **Supplementary Figures S5** and S8) is conserved in many primates (**Supplementary Figure S11**) but is not conserved in rodents and other mammals.

We also compared the expression profiles of human and bovine RPE65 (**Figure 1**). The region corresponding to the putative “AW205227.1” sisRNA does not have a larger number of hits, whereas the “BF673206.1” region has a much large number of reads (**Supplementary Figures S6** and S7). We mapped paired reads only using the rule that one read should have an overlap (> 10 nucleotides) with an intron, another read can have any location. Results of this analysis (**Supplementary Figures S6** and S7) suggested that the putative “AW205227.1” sisRNA is much less abundant (∼10 times) than the putative “BF673206.1” sisRNA. This trend is opposite to the observed trend in human RPE65 (**Supplementary Figures S6** and S7). The analysis did not suggest any putative intronic sequences in sisRNAs similar to human RPE65 (**Supplementary Figures S6** and S7).

## Discussion

Alternative splicing events seem to be relatively rare in normal human RPE, but these are much more frequent in 4M_ARPE-19 cells due to significantly higher frequency of skipping of exons 2, 3, and 7. Skipping events of exons 4 and 12 were not significantly different between native RPE samples and 4M_ARPE-19 (**Table 2**). It was concluded that a defect in splicing of exons 12 and/or 13 is associated with pathogenesis of the dominantly inherited c.1430A > G human RPE65 mutation (Li et al., 2019), but this avenue of research requires further investigation.

We discovered stable polyadenylated transcripts with sequences corresponding to the intronic region of RPE65 gene in the cytoplasm of native bovine RPE and 4M_ARPE-19 cells. At this point, it is difficult to classify them as lncRNAs or sisRNAs (some “AW205227.1” RNAs are likely to be products of an early termination of transcription in the intron 10 as suggested by the 33cd27e9-8975-4acd-adce-f033b9f9324b nanopore read that covers several exons in addition to intronic sequences, **Supplementary Table S1**). Also, there is the possibility of stable but not spliced transcripts from the RPE65 gene, although we did not detect them by the nanopore sequencing technique (**Supplementary Table S1**). Transcripts lacking exon 7 were found in both the human RPE cell line and native bovine RPE that are transcribed from the RPE65 promoter. This mis-spliced transcript is much less abundant according to qPCR data compared to normally spliced RPE65 mRNA in ARPE-19 cells (**Supplementary Figure S2**). Interestingly, this transcript is transported to the cytoplasm and apparently is stable and not rapidly degraded.

Intronic transcription was detected in intron 10, the longest intron (6.6 kb) of the RPE65 gene. One possibility is that it is the most difficult intron to remove, and therefore transcripts with unspliced intron 10 are over-represented. However, this does not explain the characteristic pattern of RNA-seq short reads being more abundant at the 5′ end of intron 10. In addition, transcripts with the unspliced intron 10 were not detected by nanopore sequencing (**Supplementary Table S1**).

The difficulty of cloning 5′RACE products of transcripts from intron 10 could be due to lariat structures present at their 5′ end. Linear sisRNAs are predicted to possess a secondary structure at their 5′ end after debranching of 5′ lariat structures with the 3′ tails being exposed (Chan and Pek, 2019; Osman et al., 2016). Some of these sisRNA were demonstrated to regulate their host genes' expression (Chan and Pek, 2019).

It is well established that, compared with protein-coding sequences and many structural RNAs, sisRNAs are weakly conserved in evolution. Many early studies, therefore, branded sisRNAs as “transcriptional dark matter” and considered them to be generally non-functional (Robinson, 2010; van Bakel and Hughes, 2009). However, low level or lack of detectable conservation does not necessarily imply that these molecules have no function (Pang et al., 2006). One example is a well-characterized, functionally important lncRNA gene, Xist, which is weakly conserved although it does contain short evolutionarily constrained regions (Elisaphenko et al., 2008). Thus, it is important to search for conserved sisRNAs by synteny instead of sequence similarity (Chan and Pek, 2019). However, the observed pattern of conservation seems to be non-trivial: the region with a large number of reads in human is different from the highly expressed region in cow (**Figure 1**). Our results suggest that these RNAs indeed are stable in cytoplasm of bovine RPE and ARPE-19 cells. The correlation analysis revealed a strong positive correlation (**Supplementary Figure S9**) between the exon 8 and intronic parts of the “AW205227.1” sisRNA (introns 9 and 10 according to the bb840fee-400c-4858-9439-fb612ed78dfe nanopore read, **Supplementary Table S1**). This may reflect the fact that this sisRNA may be non-functional and be the result of “transcriptional” noise (van Bakel and Hughes, 2009). However, we cannot exclude the possibility that these putative stable intronic RNAs could be important for RPE65 splicing or translation, taking into account that correlation analysis may be a less powerful approach for sisRNAs due to co-localization of sisRNAs and protein-coding genes (Chan and Pek, 2019). Also, these putative sisRNAs might have positive or negative regulatory effects on alternative splicing of RPE65 mRNA (Romero-Barrios et al., 2018), as suggested by differences between detected by the correlation analyses (no significant correlation for native RPE samples and a significant correlation for 4M_ARPE-19 samples, **Supplementary Figure S10**), although these possibilities need to be investigated further.

## Data Availability

Publicly available datasets were analyzed in this study. This data can be found here: https://www.ncbi.nlm.nih.gov/gap.

## Author Contributions

OP, IR, EP, and TMR contributed to the conceptualization of the study. OP, EP, IR, and NG contributed to the methodology and experimentation. IR was in charge of the software. Validation was performed by OP, IR, and NG. Formal analysis was conducted by OP and IR. Writing and original draft preparation were conducted by OP, IR, and EP. Writing, review, and editing were handled by OP, IR, NG, EP, and TMR.

## Funding

This research was supported by the Intramural Research Programs of the National Eye Institute and the National Library of Medicine, National Institutes of Health.

## Conflict of Interest Statement

The authors declare no conflict of interest. The funders had no role in the design of the study; in the collection, analyses, or interpretation of data; in the writing of the manuscript; or in the decision to publish the results.

## References

[B1] Boulton M. E. (2014). Studying melanin and lipofuscin in RPE cell culture models. Exp. Eye Res. 126, 61–67. 10.1016/j.exer.2014.01.016 25152361 PMC4143628

[B2] Bowne S. J. Humphries M. M. Sullivan L. S. Kenna P. F. Tam L. C. Kiang A. S. (2011). A dominant mutation in RPE65 identified by whole-exome sequencing causes retinitis pigmentosa with choroidal involvement. Eur. J. Hum. Genet. 19, 1074–1081. 10.1038/ejhg.2011.86 21654732 PMC3190249

[B3] Chan S. N. Pek J. W. (2019). Stable intronic sequence RNAs (sisRNAs): an expanding universe. Trends Biochem. Sci. 44, 258–272. 10.1016/j.tibs.2018.09.016 30391089

[B4] Elisaphenko E. A. Kolesnikov N. N. Shevchenko A. I. Rogozin I. B. Nesterova T. B. Brockdorff N. (2008). A dual origin of the Xist gene from a protein-coding gene and a set of transposable elements. PLoS One 3, e2521. 10.1371/journal.pone.0002521 18575625 PMC2430539

[B5] Fernandes J. C. R. Acuna S. M. Aoki J. I. Floeter-Winter L. M. Muxel S. M. (2019). Long non-coding RNAs in the regulation of gene expression: physiology and disease. Noncoding RNA 5 (1), E17. 10.3390/ncrna5010017 30781588 PMC6468922

[B6] Glazko G. B. Milanesi L. Rogozin I. B. (1998). The subclass approach for mutational spectrum analysis: application of the SEM algorithm. J. Theor. Biol. 192, 475–487. 10.1006/jtbi.1998.0668 9680721

[B7] Golestaneh N. Chu Y. Xiao Y. Y. Stoleru G. L. Theos A. C. (2017). Dysfunctional autophagy in RPE, a contributing factor in age-related macular degeneration. Cell Death Dis. 8, e2537. 10.1038/cddis.2016.453 28055007 PMC5386365

[B8] Hamel C. P. Tsilou E. Pfeffer B. A. Hooks J. J. Detrick B. Redmond T. M. (1993). Molecular cloning and expression of RPE65, a novel retinal pigment epithelium-specific microsomal protein that is post-transcriptionally regulated *in vitro* . J. Biol. Chem. 268, 15751–15757. 8340400

[B9] Jin M. Li S. Moghrabi W. N. Sun H. Travis G. H. (2005). Rpe65 is the retinoid isomerase in bovine retinal pigment epithelium. Cell 122, 449–459. 10.1016/j.cell.2005.06.042 16096063 PMC2748856

[B10] Khromov-Borisov N. N. Rogozin I. B. Pegas Henriques J. A. de Serres F. J. (1999). Similarity pattern analysis in mutational distributions. Mutat. Res. 430, 55–74. 10.1016/S0027-5107(99)00148-7 10592318

[B11] Kondrashov A. S. Rogozin I. B. (2004). Context of deletions and insertions in human coding sequences. Hum. Mutat. 23, 177–185. 10.1002/humu.10312 14722921

[B12] Lehmann G. L. Benedicto I. Philp N. J. Rodriguez-Boulan E. (2014). Plasma membrane protein polarity and trafficking in RPE cells: past, present and future. Exp. Eye Res. 126, 5–15. 10.1016/j.exer.2014.04.021 25152359 PMC4502961

[B13] Li Y. Furhang R. Ray A. Duncan T. Soucy J. Mahdi R. (2019). Aberrant RNA splicing is the major pathogenic effect in a knock-in mouse model of the dominantly inherited c.1430A > G human RPE65 mutation. Hum. Mutat. 40, 426–443. 10.1002/humu.23706 30628748 PMC6425930

[B14] Liu S. Y. Redmond T. M. (1998). Role of the 3′-untranslated region of RPE65 mRNA in the translational regulation of the RPE65 gene: identification of a specific translation inhibitory element. Arch. Biochem. Biophys. 357, 37–44. 10.1006/abbi.1998.0817 9721181

[B15] Marlhens F. Bareil C. Griffoin J. M. Zrenner E. Amalric P. Eliaou C. (1997). Mutations in RPE65 cause Leber’s congenital amaurosis. Nat. Genet. 17, 139–141. 10.1038/ng1097-139 9326927

[B16] Moiseyev G. Chen Y. Takahashi Y. Wu B. X. Ma J. X. (2005). RPE65 is the isomerohydrolase in the retinoid visual cycle. Proc. Natl. Acad. Sci. U. S. A. 102, 12413–12418. 10.1073/pnas.0503460102 16116091 PMC1194921

[B17] Morimura H. Fishman G. A. Grover S. A. Fulton A. B. Berson E. L. Dryja T. P. (1998). Mutations in the RPE65 gene in patients with autosomal recessive retinitis pigmentosa or leber congenital amaurosis. Proc. Natl. Acad. Sci. U. S. A. 95, 3088–3093. 10.1073/pnas.95.6.3088 9501220 PMC19699

[B18] Osman I. Tay M. L. Pek J. W. (2016). Stable intronic sequence RNAs (sisRNAs): a new layer of gene regulation. Cell Mol. Life Sci. 73, 3507–3519. 10.1007/s00018-016-2256-4 27147469 PMC11108444

[B19] Pang K. C. Frith M. C. Mattick J. S. (2006). Rapid evolution of noncoding RNAs: lack of conservation does not mean lack of function. Trends Genet. 22, 1–5. 10.1016/j.tig.2005.10.003 16290135

[B20] Poliakov E. Gubin A. N. Stearn O. Li Y. Campos M. M. Gentleman S. (2012). Origin and evolution of retinoid isomerization machinery in vertebrate visual cycle: hint from jawless vertebrates. PLoS One 7, e49975. 10.1371/journal.pone.0049975 23209628 PMC3507948

[B21] Postnikova O. A. Rogozin I. B. Samuel W. Nudelman G. Babenko V. N. Poliakov E. (2019). Volatile evolution of long non-coding RNA repertoire in retinal pigment epithelium: insights from comparison of bovine and human RNA expression profiles. Genes (Basel) 10 (3), E205. 10.3390/genes10030205 30857256 PMC6471466

[B22] Redmond T. M. Poliakov E. Yu S. Tsai J. Y. Lu Z. Gentleman S. (2005). Mutation of key residues of RPE65 abolishes its enzymatic role as isomerohydrolase in the visual cycle. Proc. Natl. Acad. Sci. U. S. A. 102, 13658–13663. 10.1073/pnas.0504167102 16150724 PMC1224626

[B23] Rizzolo L. J. (2014). Barrier properties of cultured retinal pigment epithelium. Exp. Eye Res. 126, 16–26. 10.1016/j.exer.2013.12.018 24731966

[B24] Robinson R. (2010). Dark matter transcripts: sound and fury, signifying nothing? PLoS Biol. 8, e1000370. 10.1371/journal.pbio.1000370 20502697 PMC2872672

[B25] Romero-Barrios N. Legascue M. F. Benhamed M. Ariel F. Crespi M. (2018). Splicing regulation by long noncoding RNAs. Nucleic Acids Res. 46, 2169–2184. 10.1093/nar/gky095 29425321 PMC5861421

[B26] Ryan M. Wong W. C. Brown R. Akbani R. Su X. Broom B. (2016). TCGASpliceSeq a compendium of alternative mRNA splicing in cancer. Nucleic Acids Res. 44, D1018–22. 10.1093/nar/gkv1288 PMC4702910 26602693

[B27] Samuel W. Jaworski C. Postnikova O. A. Kutty R. K. Duncan T. Tan L. X. (2017). Appropriately differentiated ARPE-19 cells regain phenotype and gene expression profiles similar to those of native RPE cells. Mol. Vis. 23, 60–89. http://www.molvis.org/molvis/v23/60 28356702 PMC5360456

[B28] Strauss O. (2005). The retinal pigment epithelium in visual function. Physiol. Rev. 85, 845–881. 10.1152/physrev.00021.2004 15987797

[B29] Thompson D. A. Gyurus P. Fleischer L. L. Bingham E. L. McHenry C. L. Apfelstedt-Sylla E. (2000). Genetics and phenotypes of RPE65 mutations in inherited retinal degeneration. Invest. Ophthalmol. Vis. Sci. 41, 4293–4299. 11095629

[B30] Ulitsky I. Bartel D. P. (2013). lincRNAs: genomics, evolution, and mechanisms. Cell 154, 26–46. 10.1016/j.cell.2013.06.020 23827673 PMC3924787

[B31] van Bakel H. Hughes T. R. (2009). Establishing legitimacy and function in the new transcriptome. Brief Funct. Genomic Proteomic. 8, 424–436. 10.1093/bfgp/elp037 19833698

[B32] Whitmore S. S. Wagner A. H. DeLuca A. P. Drack A. V. Stone E. M. Tucker B. A. (2014). Transcriptomic analysis across nasal, temporal, and macular regions of human neural retina and RPE/choroid by RNA-Seq. Exp. Eye Res. 129, 93–106. 10.1016/j.exer.2014.11.001 25446321 PMC4259842

