# Supplementary material for: Stable Intronic Sequences and Exon Skipping Events in the Human RPE65 Gene: Analysis of Expression in Retinal Pigment Epithelium Cells and Cell Culture Models
Source: Front Genet. 2019 Jul 19;10:634. doi: 10.3389/fgene.2019.00634 (PMC6658614; doi:10.3389/fgene.2019.00634)
Supplement: Supplementary file 1 [file Table_1.pdf]

Supplementary materials (Tables and Figures)

Stable intronic sequences and exon skipping events in the human RPE65 gene: analysis of expression in retinal pigment epithelium cells and cell culture models

Supplementary Table S1. Nanopore reads located in the RPE65 gene (ARPE-19 cells).

Read ID (ARPE19)	Brief description of reads
9016c825-eb53-4a17-a379-42d4e40d3cf7	Incomplete RPE65 mRNA, exons 11-14
a0715f31-4ebd-4adb-9ccf-fc76d8f3f7f8	Complete RPE65 mRNA
432ab81e-4271-46bf-b138-d710797bdbd1	Incomplete RPE65 mRNA, exons 4-14
c5597c60-f0ef-40e5-bd45-c0acf29eb582	“BF673206.1” sisRNA: 5’ end
00febf5a-3235-4a14-9cba-a4948eba0e5a	Incomplete RPE65 mRNA, exons 7-14
78289747-f173-41e0-87f8-f7745c4e11b3	3' UTR end
1fd7f69d-59f9-4bcd-80bc-05df9d905608	Incomplete RPE65 mRNA, exons 1-9
8513c523-e8a1-44aa-a881-1a45b7f28a69	3' UTR end
fa09503d-61eb-4954-bc72-ce29cf95b8a1	3' UTR end
2de15eb7-5d94-42ec-b6bc-092168123b83	Incomplete RPE65 mRNA, exons 12-14
04640489-b7c7-43e4-aa1a-c5fff3c6bbe3	intron 6 (partial), exon 7, inton 7, exons 8-14, early stop in 3'UTR
8429b8de-c612-4dca-b744-0ff3e7093ccf	Almost complete RPE65 mRNA (incomplete 3'UTR)
abfead6d-c832-454b-bc5e-406773fd12bf	“AW205227.1” sisRNA: 3’ end
1f4c3f6f-ea58-475e-89ba-9e96a70c93c4	Incomplete RPE65 mRNA, exons 2-14
a2cb22a7-59ce-45c1-a0aa-64875b46b05a	3’UTR end and extended 3’end
6905266a-fff4-427d-9b25-55ff8bcfa7aa	3'UTR end
928c9746-c452-4bfc-ae13-ba002e4f89c5	3'UTR end
d51a9296-ec75-4381-b967-e82ac386edd0	“AW205227.1” sisRNA: exons 1-9, intron 9, exon 10, intron 10 (partial)
e28e7c7f-e095-4244-8bc5-e86f1e32e5b0	“AW205227.1” sisRNA: 3’ end
5b069eaa-22b2-4acc-82d8-975edbcf37ff	Complete RPE65 mRNA
f7b1f02f-9ce3-41c4-8703-bf3e16ba7b30	3' UTR end
a3622fdb-3848-4cc7-b896-789e83e31809	3' UTR end
e963c893-6168-4551-af0b-c5d77ce789eb	Complete RPE65 mRNA
43b6d550-0bdb-4335-b8dc-0318acca5000	3'UTR end
c9bd1a7e-d69c-4c61-8bc6-99fba7a6267f	Complete RPE65 mRNA
29755ad7-6e08-4bc5-ae6f-71fdb0325219	3' UTR end
bb840fee-400c-4858-9439-fb612ed78dfe	“AW205227.1” sisRNA: intron 9 (partial), exon 10, intron 10 (partial)
33cd27e9-8975-4acd-adce-f033b9f9324b	“AW205227.1” sisRNA: exons 2-3, 5-9, intron 9, exon 10, intron 10 (partial)
6e98f670-c619-41cd-90ea-62b4fca5a437	3'UTR
294f75b3-df06-45a0-bcd9-0de09d900751	Complete RPE65 mRNA

Sequence information is provided in the Supplementary Figure S8 and the Supplementary Data File[1].

Supplementary Table S2. Primers used in this study.

PCR primers	F	R
BF673206.1 EST human	CCCTCATCTATAGCTTCCTGCA	AACATTTGTTCACTCCCGTGTG
BF673206.1 EST bovine	TCAGTGACTGACATGGACATGG	CCTATACCATCTCACCTTGGCG
AW205227.1 EST human	TCAGAAGAGTACTGTGACTTAG	GTCTCTAAATACAGGGAAGA
AW205227.1 EST bovine	TCCTGCTGTGTGTTTATAC	ATCCTTAATACCCTACGAC

RACE primers for BF673206.1 EST human	
GSP2-3'	CGAGGCCCTGAAAAGAGAACTTCAGGCTCC
NGSP2	GCGTGACTAAATTCTTGCCTGTGTCAGCCTAGG
GSP1-5'	CCCTCATCTATAGCTTCCTGCAGTTCCTCCCTG
NGSP1	TCACGCTCCCCAATACAACTGCCACTGC
RACE primers for AW205227.1 EST human	
GSP1	aagcttggcgtaatcGGAATCAGTGGATCTGCACTATTCACCG
NGSP1	aagcttggcgtaatcACCTGGTTCTTATCCTGGCTCTGCTGC
GSP2	GATTACGCCAAGCTTAGCAAAAGCAACTGCAGCAGAGCCAG
NGSP2	GATTACGCCAAGCTTCTTCCTCGGTGAATAGTGCAGATC
RACE primers for AW205227.1 EST bovine	
GSP1	aagcttggcgtaatcATCCTTAATACCCTACGACCAGTC
NGSP1	aagcttggcgtaatcCACTTTGACTCTGTCATAACTGGAT
GSP2	GATTACGCCAAGCTTAGTGAAACTGAAGCTCATGACTTAG
NGSP2	GATTACGCCAAGCTTCTTTGAAGGCTTTTAGGCTAGGC
RACE primers for BF673206.1 EST bovine	
GSP1	aagcttggcgtaatcGTTCTTGGTCTCGCCCTTCTAGA
NGSP1	aagcttggcgtaatcCTAAACTTGGAAACTGTTCTCACAG
GSP2	GATTACGCCAAGCTTGAAAGAGGATAACAGAAACCTCAGT
NGSP2	GATTACGCCAAGCTTCAGAAAACTCTAGAAGGGCGAGAC

Supplementary Table S3. Data table for figure S3. Percentage of RPE65 mRNA in cytoplasmic (cyt) and nucleus (nucl) fraction measured by qPCR. Ct values for no RT control are shown for each primer pair. Primers located in exons 13 and 14 give more noise as evident by no RT Ct value (Ct = 30.5 corresponds to amplification without reverse transcriptase from genomic DNA) and this noise contributes to signal especially from nucleus with more genomic contamination. Because of this issue, interpretation of qPCR data may be problematic.

	Cyt1	Cyt2	Cyt3	Nucl1	Nucl2	Nucl3	No RT Ct
RPE65 10-11 ex	93.65997	90.48379	58.50825	6.340032	9.516207	41.49175	36.913
RPE65 13-14 ex	23.77484	24.08809	54.9611	76.22516	75.91191	45.0389	30.461

(A)

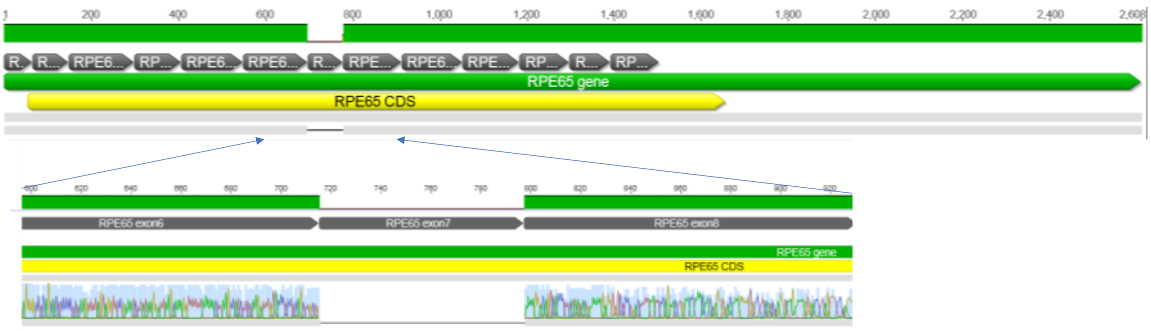

(B)

>rpe65-noex7-ARPE19
TCCTTCTTCATTCTGCAGTTGGTGCCAGAACTCTGGATCCTGAACTGGAAGAAAATGTCTATCCAGGTTGAGCATCCTGC
TGGTGGTTACAAGAAACTGTTTGAAACTGTGGAGGAACTGTCCTCGCCGCTCACAGCTCATGTAACAGGCAGGATCCCCC
TCTGGCTCACCGGCAGTCTCCTTCGATGTGGGCCAGGACTCTTTGAAGTTGGATCTGAGCCATTTTACCACCTGTTTGAT
GGGCAAGCCCTCCTGCACAAGTTTGACTTTAAAGAAGGACATGTCACATACCACAGAAGGTTCATCCGCACTGATGCTTA
CGTACGGGCAATGACTGAGAAAAGGATCGTCATAACAGAATTTGGCACCTGTGCTTTCCCAGATCCCTGCAAGAATATAT
TTTCCAGGTTTTTTTCTTACTTTCGAGGAGTAGAGGTTACTGACAATGCCCTTGTTAATGTCTACCCAGTGGGGGAAGAT
TACTACGCTTGCACAGAGACCAACTTTATTACAAAGATTAATCCAGAGACCTTGGAGACAATTAAGCAGGTTGATCTTTG
CAACTATGTCTCTGTCAATGGGGCCACTGCTCACCCCCACATTGAAAATGATGGAACCGTTTACAATATTGGTAATTGCT
TTGGAAAAAATTTTTCAATTGCCTACAACATTGTAAAGATCCCACCACTGCAAGCAGTTTTGGTCTGACTCCCAACTATA
TCGTTTTTGTGGAGACACCAGTCAAAATTAACCTGTTCAAGTTCCTTTCTTCATGGAGTCTTTGGGGAGCCAACTACATG
GATTGTTTTGAGTCCAATGAAACCATGGGGGTTTGGCTTCATATTGCTGACAAAAAAAGGAAAAAGTACCTCAATAATAA
ATACAGAACTTCTCCTTTCAACCTCTTCCATCACATCAACACCTATGAAGACAATGGGTTTCTGATTGTGGATCTCTGCT
GCTGGAAAGGATTTGAGTTTGTTTATAATTACTTATATTTAGCCAATTTACGTGAGAACTGGGAAGAGGTGAAAAAAAAT
GCCAGAAAGGCTCCCCAACCTGAAGTTAGGAGATATGTACTTCCTTTGAATATTGACAAGGCTGACACAGGCAAGAATTT
AGTCACGCTCCCCAATACAACTGCCACTGCAATTCTGTGCAGTGACGAGACTATCTGGCTGGAGCCTGAAGTTCTCTTTT
CAGGGCCTCGTCAAGCATTTGAGTTTCCTCAAATCAATTACCAGAAGTATTGTGGGAAACCTTACACATATGCGTATGGA
CTTGGCTTGAATCACTTTGTTCCAGATAGGCTCTGTAAGCTGAATGTCAAAACTAAAGAAACTTGGGTTTGGCAAGAGCC
TGATTCATACCCATCAGAACCCATCTTTGTTTCTCACCCAGATGCCTTGGAAGAAGATGATGGTGTAGTTCTGAGTGTGG
TGGTGAGCCCAGGAGCAGGACAAAAGCCTGCTTATCTCCTGATTCTGAATGCCAAGGACTTAAGTGAAGTTGCCCGGGCT
GAAGTGGAGATTAACATCCCTGTCACCTTTCATGGACTGTTCAAAAAATCTTGAGCATACTCCAGCAAGATATGTTTTTG
GTAGCAAAACTGAGAAAATCAGCTTCAGGTCTGCAATCAAATTCTGTTCAATTTTAGCCTGCTATATGTCATGGTTTTAA
CTTGCAGATGCGCACAATTTTGCAATGTTTTACAGAAAGCACTGAGTTGAGCAAGCAATTCCTTTATTTAAAAAAAAAAG
TACGTATTTAGATAATCATACTTCCTCTGTGAGACAGGCCATAACTGAAAAACTCTTAAATATTTAGCAATCAAATAGGA
AATGAATGTGGACTTACTAAATGGCTTTTAATTCCTATTATAAGAGCATATTTTAGGTACCTATCTGCTCCAATTATATT
TTTAACATTTAAAAACCAAAGTCCTCTACACTTGATTTATATTATATGTGGCTTTGCTGAGTCAAGGAAGTATCATGCAA
TAAGGCTTAATTACTAAATGTCAAACCAAACTTTTTCTCAAACCAGGGACTATCATCTAAGATTAATTACAGTAATTATT
TTGCGTATACGTAACTGCTCAAAGATTATGAATCTTATGAATGTTAACCTTTCCGTTTATTACAAGCAAGTACTATTATT
TCTGATTTTATAATAAGAAAATCTGTGTTTAATCAACTGAGGCCTCTCAACCAAATAACATCTCAGAGATTAAGTTATAT
ATTAAAAGCTTATGTAACATAAAAGCAAGTACATATAGTAGTGACTATATTTAAAAAAACAGCATAAAATGCTTAAAAAT
GTAATATTTACTAAAATCAGATTATGGGATAATGTTGCAGGATTATACTTTATTGCATCTTTTTTGTTTAATTGTATTTA
AGCATTGTGCAATCACTTGGGAAAAATATTAAATTATTAACATTGAGGTATTAATACATTTTAAGCCTTTTGTTTTTAAA
TTTCTTTTCTTCCAGAGATTGTTTAAAAATAAATATTGACAAAAAT

>AW-bov-5RACE-no exon 7
CCTCAGTCCACAGTTGGTGCCAGAACTCTCAATCCTCTACTGAGAGAAAATGTCCAGCCAAGTTGAACATCCAGCTGGTG
GTTACAAGAAACTGTTTGAAACTGTGGAGGAACTATCCTCACCGCTCACAGCCCATGTTACAGGCAGGATCCCCCTCTGG
CTAACCGGCAGTCTCCTTCGATGTGGGCCAGGACTCTTTGAGGTTGGATCGGAACCATTTTACCACCTGTTTGATGGGCA
AGCCCTCCTACACAAGTTTGACTTTAAAGAAGGACATGTCACATACCACAGAAGGTTCATCCGCACTGATGCTTACGTAC
GGGCAATGACTGAGAAAAGGATCGTCATAACAGAATTTGGCACCTGTGCTTTCCCAGATCCCTGCAAGAATATATTTTCC
AGGTTTTTTTCTTACTTCCGAGGAGTGGAGGTTACTGACAATGCCCTTGTTAATATCTACCCAGTGGGGGAAGATTACTA

```
TGCCTGCACAGAGACCAACTTCATTACAAAGGTTAATCCTGAGACCTTGGAAACAATTAAGCAGGTTGACCTTTGCAACT
ATGTCTCAGTCAATGGAGCCACTGCTCACCCCCACATTGAAAATGATGGGACTGTTTACAACATTGGTAATTGCTTTGGG
AAAAATTTTTCAATTGCCTACAATATTGTAAAGATCCCACCACTACAAGCAGTTTTGGTTTGACTCCCAACTATATTGTT
TTTGTGGAGACACCAGTCAAAATTAATCTGTTCAAGTTTCTTTCTTCATGGAGTCTTTGGGGAGCCAATTACATGGATTG
TTTTGAATCCAATGAAACCATGGGGGTTTGGCTTCATATTGCTGACAAAAAAAGAAAAAAGTATATCAATAATAAATA
```

Supplementary figure S1. (A) Schematic representation of the RPE65 isoform without the exon 7. (B) Experimentally detected sequences of the isoform.

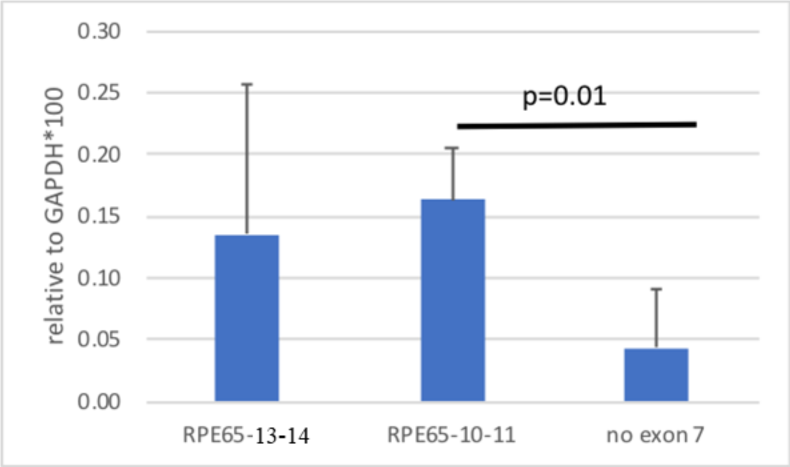

Supplemental figure S2. Expression level of different RPE65 isoforms in ARPE-19 cells relative to GAPDH multiplied by 100.

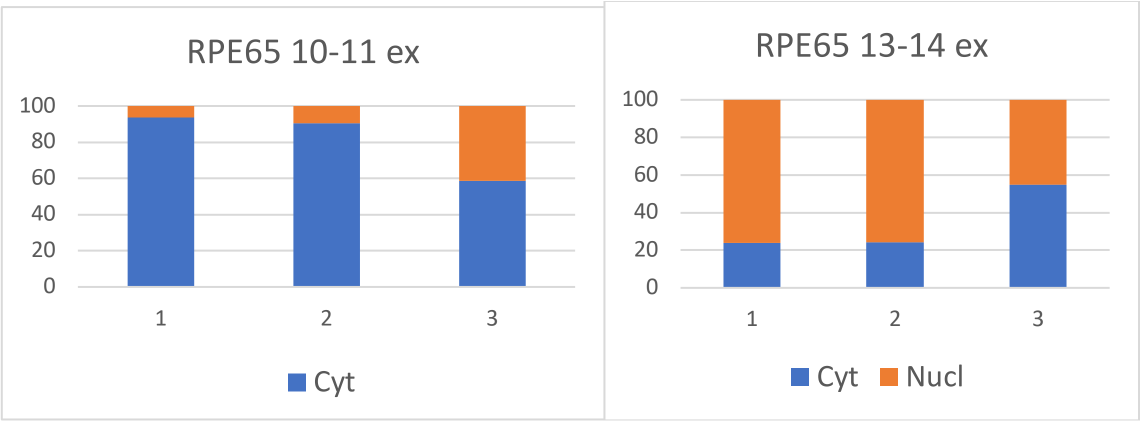

Supplementary figure S3. Distribution of RPE65 mRNA across cytoplasmic (cyt, shown in blue) and nucleus (nucl, shown in orange) fraction from three biological replicates (1-3) measured by qPCR using primers in exons 10 and 11 (spliced intron 10) and exons 13-14 (spliced intron 13).

)

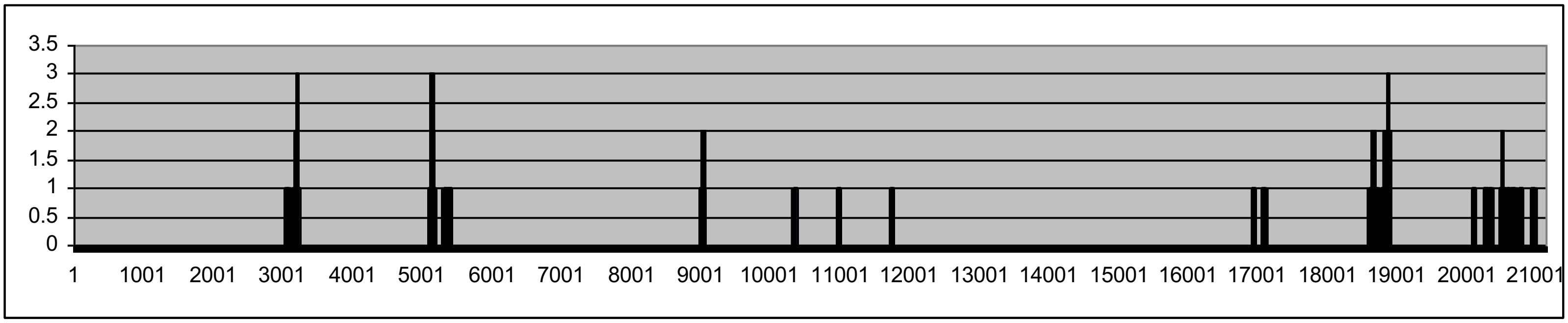

)

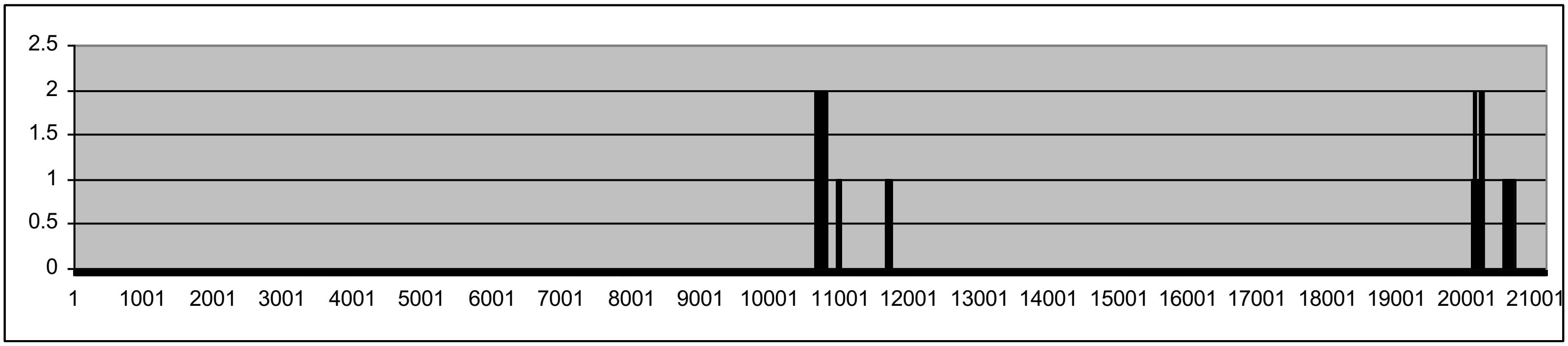

Supplementary Figure S4. Distribution of the number of raw reads (Y axis) in each position of human RPE65 mRNA sequences mapped on the human genomic sequences (X axis). The position one corresponds to the first position of RPE65 mRNA (NM_000329.2). A) Merged RNAseq data for Primary human RPE cells, B) Merged RNAseq data for human ARPE-19 cell line (4 day old).

Positions of exons:

Exon 1	1	65
Exon 2	1254	1336
Exon 3	3100	3250
Exon 4	5077	5184
Exon 5	5288	5429
Exon 6	8960	9107
Exon 7	10318	10399
Exon 8	10637	10769
Exon 9	10879	11018
Exon 10	11644	11773
Exon 11	18375	18489
Exon 12	18584	18678
Exon 13	18784	18895
Exon 14	20033	21136

(A)

ACAAATGAAACTAAAGCTCAGAAGAGTACTGTGACTTAGCAAAAGCAACTGCAGCAGAGCCAGGATAAGA
ACCAGGTTTTTAAATTCTAAATCTGCCGTTCTTGTATTTACAAAATTGTCTTTAAAATATACTTCCTCGG
TGAATAGTGCAGATCCACTGATTCCTATGATACTTTATCAATAATATAAATAGTTTAAATAATTTGAAGT
TCTGGAGAGATGAAGCATCAAAAAGTACTCTTGTAGGGAAACCAGTTCATTATTTTGGTTTTCTATAAAT
CTTCTGAAAAATATCACTAGAGGTTAAAAATCATTACTAACAATAGACAGTCATCTCTTTATTTATACTT
TTTCTTCCCTGTATTTAGAGACTGTCTTTTTAGAATAACCAGAGTAACATCTAGAATTTGTAGTGTGATG
GAAATACCAGTCAAAATC**AATAAAA**TATAATTCTCACATaaaaaaaaaaaaaaa

(B)

GTTGTCTGCAGAAAAACATTAATTATACTATATGGTTTGAGATCTTGTTGGTTTTGGTTTTGTTTTTCAG
AGGCCTTTCTTTGGAGCCCTAAATTTTCTTCAAGGATGGTCTCATTTTACTGAAGAGAAAATTGATGGCC
TAAGAGGTTATGTGACTTTCCCAGTGATACATAGCCAGTGACTGACACAGGCACAGATAGCTTGCCCCAG
TCGCGTCCCAGGTCCCTCATCTATAGCTTCCTGCAGTTCCTCCCTGCATGTTGACCTAAAAAAGAACTTA
GGAGCAAGACTTAAGAACTCTGCATTTCTGGCTGTTGAATTCTTTCCTGCTCACTGAGGTTCTGTATCTT
CTCTCCTAGGCTGACCCCAGGCACAGAATTTAGTCACGCTCCTCAATACCAACTGCCACTGCAATTCTGT
GCAGTGACGAGACTATCTGGCTGGGAGCCTGAAGTTCTCTTTTCAGGCGCTCGTCCAGGTGAGATGATCT
AGACGAACCTTCACACGGGAGTGAACAACATGTTTCTTTCCAAGAGCTCACGAGTTTCCTACGGCATGTG
CTCTATTTCGTACGCATTGCGGTTTCCTCCAATCATTACCGACGTCTGGTGGGAACCTTACACTTTTGCG
TTATGACTTGCTGGATCATTGTCCAACAGGTATTAACCTTCCTATATCTTGACCGGCTTGAGGTTTGCTA
CCAGTAAG

Supplementary Figure S5. ESTs from the human intron 10 (AW205227.1) (A), and from the human intron 10, exon 11 and intron 11 (BF673206.1) (B).

A) putative "AW205227.1" sisRNA

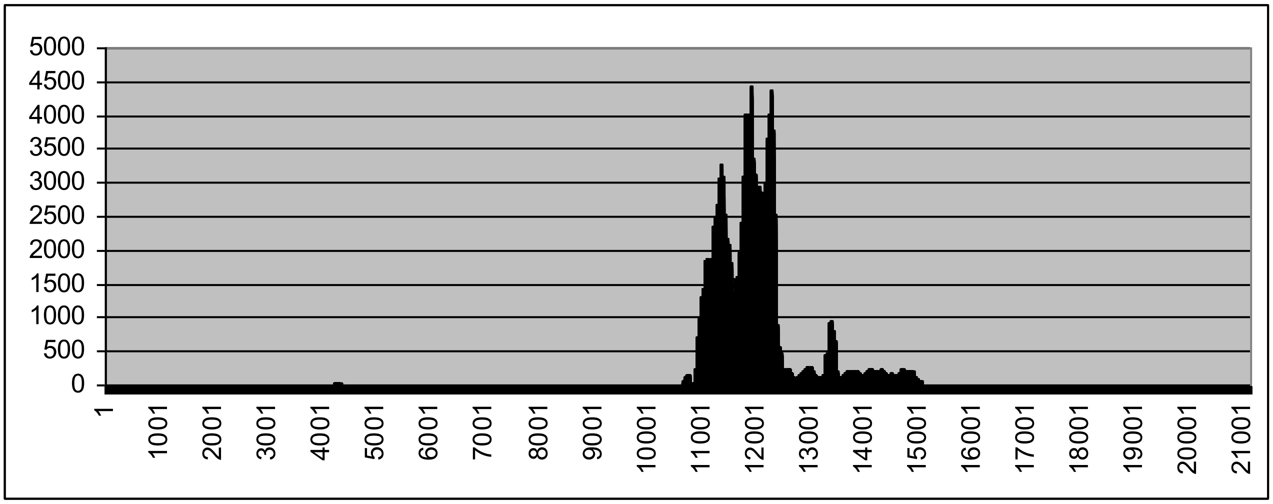

B) Bovine orthologous regions

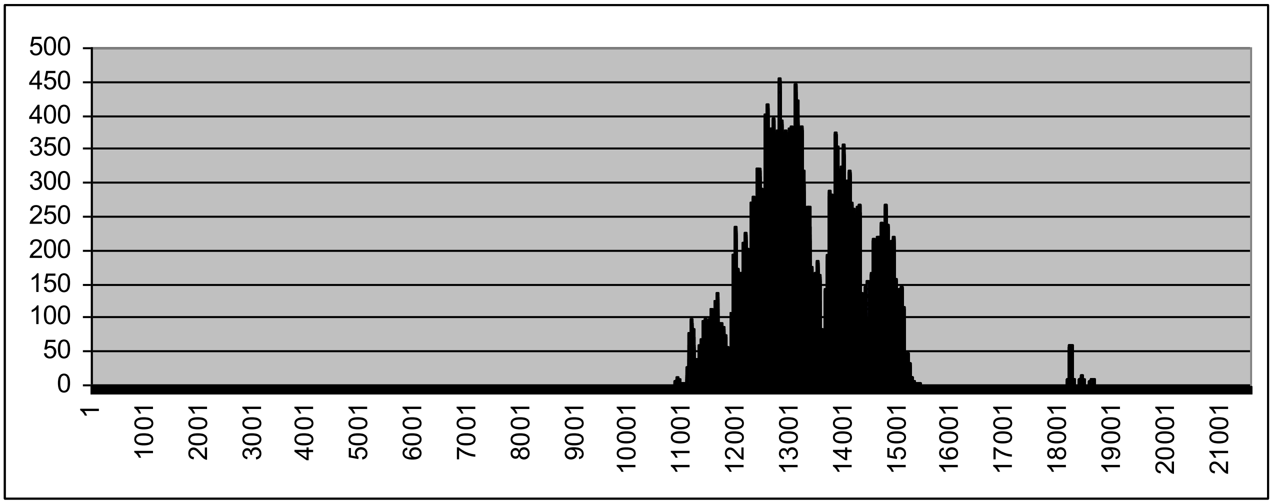

Supplementary Figure S6. Distribution of raw reads (native human/bovine RPE, Y axis) in each position of the putative human (A) / bovine (B) "AW205227.1" sisRNA sequence mapped on the human/bovine genomic sequences (X axis). The position one corresponds to the first position of RPE65 mRNA (NM_000329.2). We mapped paired reads only using the rule that at least one read should have an overlap (>10 nucleotides) with an intron, another read can have any location. Positions of bovine exons: Exon1 1 65, Exon2 1254 1336, Exon3 3100 3250, Exon4 5077 5184, Exon5 5288 5429, Exon6 8960 9107, Exon7 10318 10399, Exon8 10637 10769, Exon9 10879 11018, Exon10 11644 11773, Exon11 18375 18489, Exon12 18584 18678, Exon13 18784 18895, Exon14 20033 21136.

A) Putative “BF673206.1” sisRNA

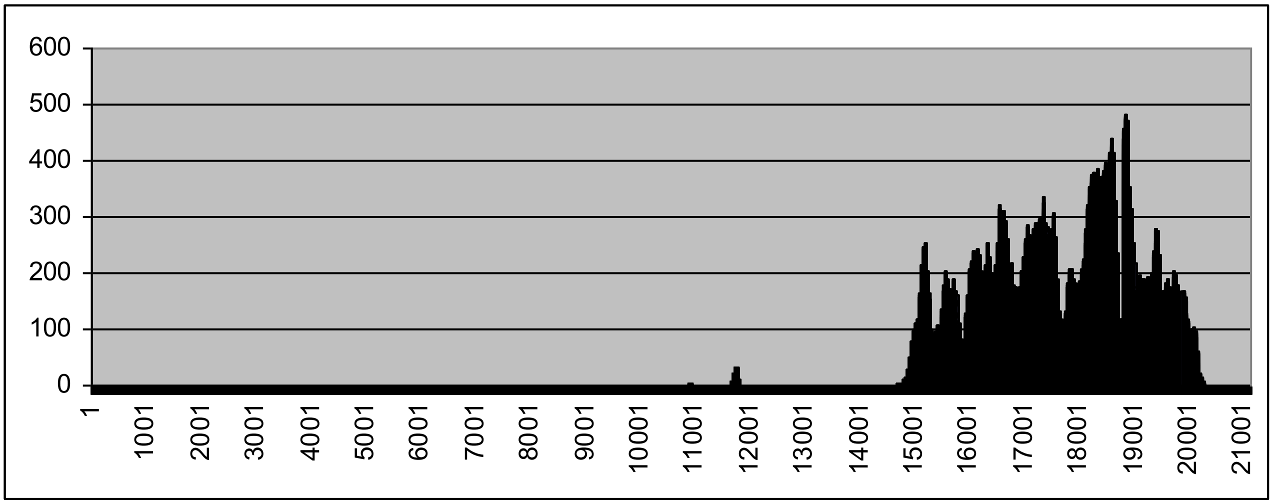

B) Bovine orthologous regions

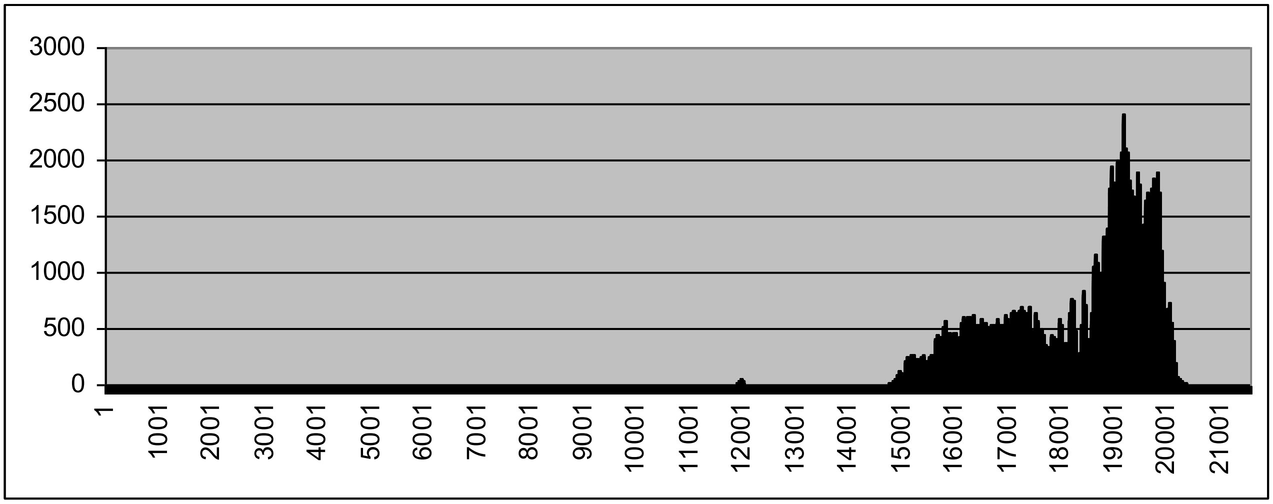

Supplementary Figure S7. Distribution of raw reads (native human/bovine RPE, Y axis) in each position of the putative human (A) / bovine (B) “BF673206.1” sisRNA sequence mapped on the human/bovine genomic sequences (X axis). The position one corresponds to the first position of RPE65 mRNA (NM_000329.2). We mapped paired reads only using the rule that at least one read should have an overlap (>10 nucleotides) with an intron, another read can have any location.

```
Query  44         GGGCAGGAGGAAATAGCTCTGATACACACCTAGCTCAATAACGGTTTTCTGGGTTGTGGA  103
                  |||| ||||||||| ||||||||||||  || |||||||| | |||| ||||||||||||
Sbjct  68438473   GGGCGGGAGGAAATGGCTCTGATACAC--CTGGCTCAATAGCAGTTT-CTGGGTTGTGGA  68438417

Query  104        ATAA-GAACAGGCAGGCACCTTGTGC---AAAGAGCAAGAATCATCTCTCTAAAATTATT  159
                  |||| |||||||||||||| ||||||   |||  ||||||||||||||||||||||||||
Sbjct  68438416   ATAAAGAACAGGCAGGCAC-TTGTGCTTAAAAAGGCAAGAATCATCTCTCTAAAATTATT  68438358

Query  160        TGTCGT-GCCTGTGCTCATGTTTGACTTTAT---TTTGCAGATTGAGGTTTGTTTATAAT  215
                  |||| | |||||||||||||||||||||| |   ||||||||||   |||||||||||||
Sbjct  68438357   TGTCATTGCCTGTGCTCATGTTTGACTTTTTATTTTTGCAGATTTGAGTTTGTTTATAAT  68438298

Query  216        TA--TATATTTAGCGA--TTACGTGAGAACTGGGAAGAGGTGAAAAAAAAAAAAATACCA  271
                  ||  |||||||||| |  ||||||||||||||||||||||||||||||||     | |||
Sbjct  68438297   TACTTATATTTAGCCAATTTACGTGAGAACTGGGAAGAGGTGAAAAAAAA-----TGCCA  68438243

Query  272        GAAAAGGCTCCC-AACCTGA-GTTGGAAGATATGTACTTCCTTTGAATATTGACAAGGTA  329
                  |||| ||||||| ||||||| ||| | |||||||||||||||||||||||||||||||||
Sbjct  68438242   GAAA-GGCTCCCCAACCTGAAGTTAGGAGATATGTACTTCCTTTGAATATTGACAAGGTA  68438184

Query  330        ACCTGCTTCTCTGTAGATTTCAGATTTAACCAGAATGTTTTCATCTCTCTCAAGAATTGT  389
                  ||||||||||||||||||||||||||||||||||||| |||||||||||||| |||||||
Sbjct  68438183   ACCTGCTTCTCTGTAGATTTCAGATTTAACCAGAATG-TTTCATCTCTCTCAGGAATTGT  68438125

Query  390        CCTCCTGCCTCATGTTTAT-CATAAAGTCTTGAAATTT--GAGCTA----GAGCTTTAAA  442
                  ||||||||||||||||||| ||||||||||||||||||  ||||||    ||||||||||
Sbjct  68438124   CCTCCTGCCTCATGTTTATACATAAAGTCTTGAAATTTGAGAGCTAGAAGGAGCTTTAAA  68438065

Query  443        AATAGACT-AGTAAACTTCCTTCATTTACAAAATGAAACAAAAGCTCAGAAAAATTACTG  501
                  ||||| || | ||||||||||||||||   ||||||||| ||||||||| || | |||||
Sbjct  68438064   AATAGGCTAATTAAACTTCCTTCATTTTACAAATGAAACTAAAGCTCAG-AAGAGTACTG  68438006

Query  502        TGACTTAACCAA--CAACTACCCAGCAAGAGCC------AGAACCAGGTTTTAAATTCCT  553
                  ||||||| | ||  |||||   |||| ||||||      ||||||||||||| || | ||
Sbjct  68438005   TGACTTAGCAAAAGCAACT--GCAGC-AGAGCCAGGATAAGAACCAGGTTTTTAAATTCT  68437949

Query  554        AAATCTGCCGTTCTTGTATTT--CAGAGTGTCTTTAAAATATACTTCCTCGGTGAATAGT  611
                  |||||||||||||||||||||   | | ||||||||||||||||||||||||||||||||
Sbjct  68437948   AAATCTGCCGTTCTTGTATTTACAAAATTGTCTTTAAAATATACTTCCTCGGTGAATAGT  68437889

Query  612        GCAGATCCACTGATTCCTATGATACTTTATCAATAGCCATAAATGGTTTAAATAATTTGA  671
                  |||||||||||||||||||||||||||||||||||   |||||| |||||||||||||||
Sbjct  68437888   GCAGATCCACTGATTCCTATGATACTTTATCAATA-ATATAAATAGTTTAAATAATTTGA  68437830

Query  672        AGTTCTACTGGA-AGATGAAACATCAAAAAGTACTCTTGTAG---AAACAGTTCATTA--  725
                  ||||   ||||| ||||||| |||||||||||||||||||||   || ||||||||||
Sbjct  68437829   AGTT---CTGGAGAGATGAAGCATCAAAAAGTACTCTTGTAGGGAAACCAGTTCATTATT  68437773

Query  726        TTGGTTTTCTAT-CGTCTTCACAGAAAAATATCACTAG-GGTTAAAAATCATTACTAACA  783
                  ||||||||||||   ||||  | ||||||||||||||| |||||||||||||||||||||
Sbjct  68437772   TTGGTTTTCTATAAATCTT--CTGAAAAATATCACTAGAGGTTAAAAATCATTACTAACA  68437715

Query  784        ATAGACAGTCATCTCTTTTTATTTC-AC-TTTTCTTCCCTGTATTTAGAAGACTGTC--T  839
                  |||||||||||||||  |||||||| || ||||||||||||||||||| ||||||||  |
Sbjct  68437714   ATAGACAGTCATCTC--TTTATTTCTACTTTTTCTTCCCTGTATTTAG-AGACTGTCTTT  68437658

Query  840        TTAGAATAACAAATGT--CTTATCGAATTTGTAGTGTGAT-GCGATACCAGTCAAAATCA  896
                  |||||||||| |  ||  | | | |||||||||||||||| |  ||||||||||||||||
Sbjct  68437657   TTAGAATAACCAGAGTAACATCTAGAATTTGTAGTGTGATGGAAATACCAGTCAAAATCA  68437598

Query  897        ATAAAATATAATTCTC  912
                  ||||||||||||||||
Sbjct  68437597   ATAAAATATAATTCTC  68437582
```

Supplementary Figure S8. Alignment of the bb840fee-400c-4858-9439-fb612ed78dfe read and Homo sapiens chromosome 1, GRCh38. The exon 9 is underlined, the putative poly-A signal is shown in green.

(A)

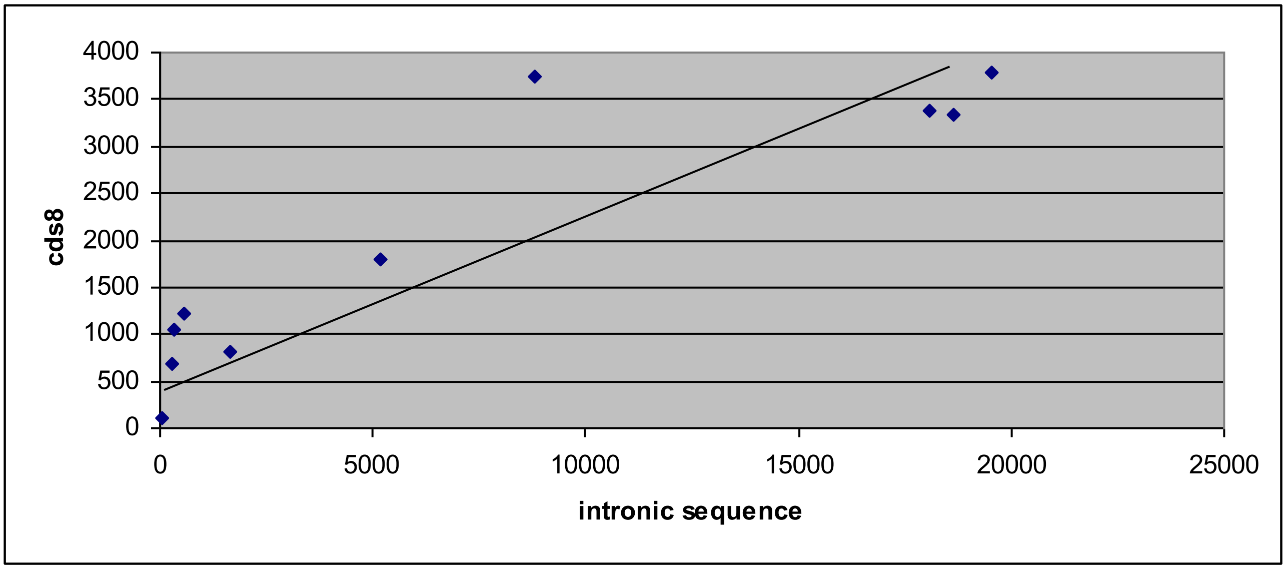

(B)

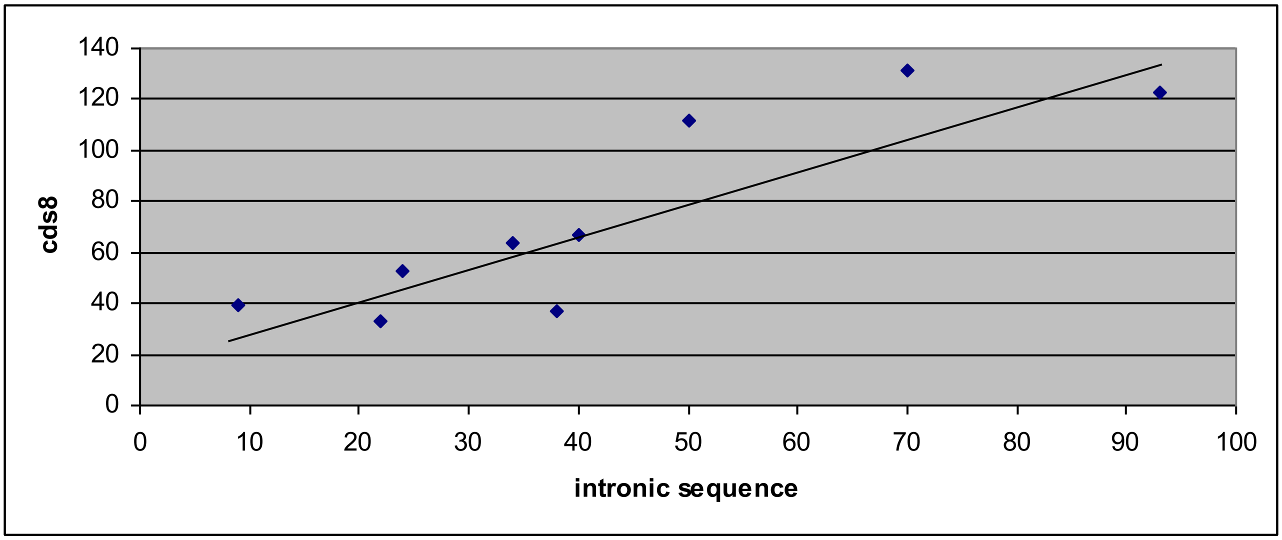

Supplementary Figure S9. Correlation between raw number of reads of the exon 8 and intronic parts of the "AW205227.1" sisRNA for: (A) native RPE samples (CC = 0.90, P = 0.0004); and (B) 4M_ARPE-19 samples (CC = 0.87, P = 0.0.002). Correlation was across different samples: 10 samples for native RPE and 9 samples for 4M_ARPE-19.

(A)

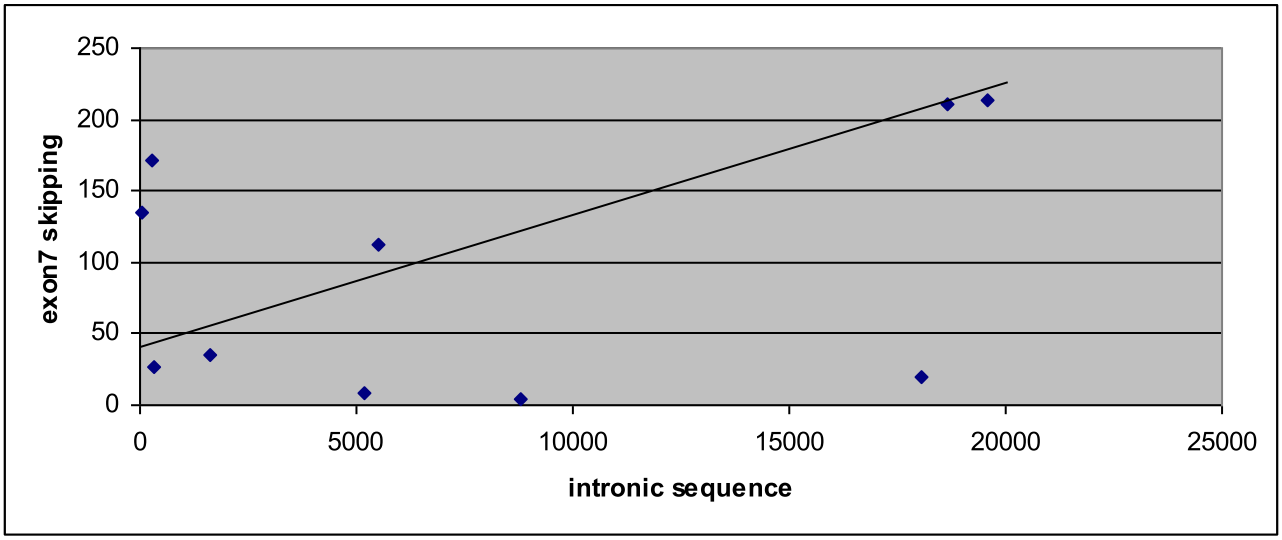

(B)

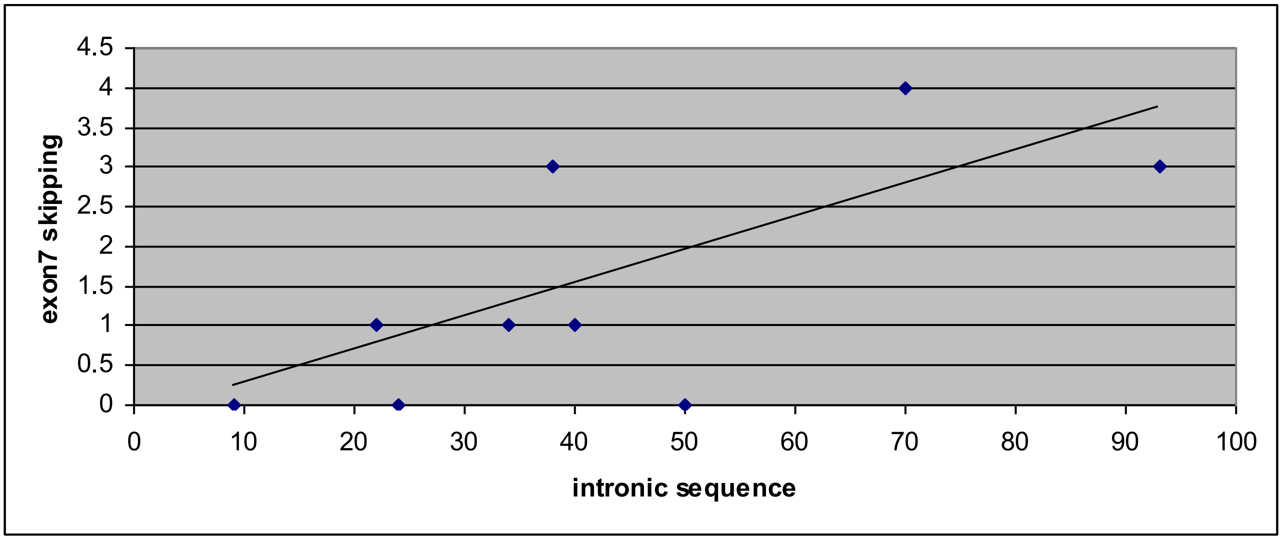

Supplementary Figure S10. Correlation between raw number of reads of exon 7 skipping events and intronic parts of the "AW205227.1" sisRNA for: (A) native RPE samples (CC = 0.90, P = 0.0004); and (B) 4M_ARPE-19 samples (CC = 0.5, P = 0.0.03). Correlation was estimated across different samples: 10 samples for native RPE and 9 samples for 4M_ARPE-19.

```
              Human  atgtgagaattatattttattga
              Chimp  atgtgagaattatattttattga
             Bonobo  atgtgagaattatattttattga
            Gorilla  atgtgagaattatattttattga
          Orangutan  atgtgagaattatattttattga
             Gibbon  atgtgagaattatattttattga
             Rhesus  atgttagaattatattttattga
Crab-eating macaque  atgttagaattatattttattga
 Pig-tailed macaque  atgttagaattatattttattga
     Sooty mangabey  atgttagaattatattttattga
             Baboon  atgttagaattatattttattga
       Green monkey  atgttagaattatattttattga
              Drill  atgttagaattacattttattga
   Proboscis monkey  atgttagaattatattttattga
    Angolan colobus  atgttagaattatattttattga
           Marmoset  atgtgagaattttattttattga
    Squirrel monkey  atgtgagaattatattttattga
White-faced sapajou  atgtgagaattaaattttattga
  Ma's night monkey  atgtgagaattatattttattga
            Tarsier  gtgtgaaaattacaccttcttga
        Mouse lemur  acgtgaaaataatatttttcttga
  Coquerel's sifaka  acgtgaaaataatatttttcttga
        Black lemur  gcatgaaaataatatttttcttga
    Sclater's lemur  gcatgaaaataatatttttcttga
           Bushbaby  ac--gaaagtg--atttttcttga
Consensus                           TTTATT
```

Supplementary Figure S11. Conservation of the putative poly-A AATAAA/TTATTT signal (the complimentary strand) for the “AW205227.1” sisRNA.
