# Supplementary material for: Stable Intronic Sequences and Exon Skipping Events in the Human RPE65 Gene: Analysis of Expression in Retinal Pigment Epithelium Cells and Cell Culture Models
Source: Front Genet. 2019 Jul 19;10:634. doi: 10.3389/fgene.2019.00634 (PMC6658614; doi:10.3389/fgene.2019.00634)
Supplement: Supplementary file 2 [file DataSheet_1.pdf]

[1]Supplementary Data File for Supplementary Table S1:

Nanopore reads within the RPE65 gene

>@9016c825-eb53-4a17-a379-42d4e40d3cf7 runid=1a9a02
TGACGAGACCATCTGGCTGAGCCTGAAGTTCCTTTCAGGTCTTCGTCACATTTGAGTTTCAAATCAATTA
CCAGAAGTATTGTGGAAACCTTACACATATGCGTATGGACTTGGCTTGAATCACTTTGTTCCAGATAGG
CTCTGTAAGCTGAATGTCAAAAAACTAAAGAAACTTGGGTTTGGCAAGAGCCTGATTCATACCCATCAGA
ACCCATCTTTGTTTCTCACCCAGATGCCTTGGAAGAAGATGATGGTGTAGTTCTGGAGGTGGTGGTAGCC
CAGGAGCAGGACAAAAGCCTGCTTATCTCCTGATTCTGAATGCCATGATTCTATGAAGTTGCCTGGGCTG
AAGTGAGATTACATCCCTGTCACCTTCATGGACTGTTCAAAAATCTTGAGCATACCCAGCAAGATATGTT
TTTCTTGGTAGCAAACTGAGAAAAATCAGCTTCAGGTCTGTAATCAAATTCTGTCTAATTTAGCTCGTTA
TATGTCATGGTTTTAATCTCTATAGATGCACAATTTTGCAAATGTTTTACAGAAAGCACTGAGTTGAGTA
AGCACCCTTTATTTAAAAAAAAAAAAAAAAAGTACGTATTTAGATAATCATACCCTGTGAGACAGGCCATA
ACTGAAAAAACTCTTAAATATTTAGCAATCAAATAGAAATGAATGTGGACTTACTAAATGGCTTTTAATT
CCTCATTATAAGAGCATATTTTAGTACCTATCTGCTCCAATTATATTTTTTGTTATTTGGGAAAACCAAA
GTCCTCTACACTTGATTTATATTATATGTGGCTTTGCTGAGTCAAGGAAGTATCATGCAATAAGGCTTAA
TTACTAAATGTCGAACCAAACTTTTTCTCAAACCAGGACTATCATCTAAAGATTAATTACAGTAATTATT
TTATGCATACGCAATCAGTCTTCAAAGGCTATGAATCTTATGACGTTAACCTTCCGTTTATTATTACAAG
CAAGTACTATTATTTCTGATTTTATAATAAGAAAATCTATGTTTAATCAACTGAGGCCTCTCAACCAAAT
AACATCTCAGAGATTAAGTTATATTAAAGCTTATGTAATATAAAGGTACATATAGTAGTGACTATATTAT
TTAAAAATAGAGCATAAAATGCTTAAAAATGCAATATTTACTAAAATCAGATTATGGGATAAATGTTGCA
GGATTATACTTTTTATTGCATCTTTTTGTTTAATTGTATTTAAGCATTGTGCAATCACTTGGGAAAAATA
TTAAATTATTAACATTGAGGTATTAATATTTTAAGCCTTTTGTTTTTAAATTTCTTTGTTCCAGAGATTG
TTTAAAAATAAATATTGACAAAAAAATAAAAAAAAAAAAAAAAAAAAAAAAAAAAAAACAAACAAAAAAAAA
AAAAAAAAGAAGATAGAGCGACAGGCAAGTAGCAATACGTAACTGAACGAAGTTTG

>@a0715f31-4ebd-4adb-9ccf-fc76d8f3f7f8 runid=1a9a02
TTACGTATTGCTTTTCTGTTGGTGCTGATATTGCTGGGGCTTCATTCTGGCAGTTGGTGCCAGAACCGAT
CCTGAACTGGAAGAAACTATCCAGGTTGAGCATCCTGCTGGTGGTTACAAGAGAAACTGTTTGAAACTCG
TGGAGGAACTGTCCTCGCTCACAGCTCATGTAACAGGCAGGATCCCCTCTGGCTCACCGGCAGTCTCCTT
CGATGTGGGCCAGACTCTTTGAAGTTGGATCTGAGCCATTTTACCACCTGTTTGATGGGCAAGCCCTCCT
GCACAAGCTTGACTTTAAAGAAGGACATGTCACATAAATTACTGTGAAGGTTCATCCGCACTGATGCTTT
CATGTACGGGCAAATGACTGAGAAAAGGATCGTCATAACAGAATTTGGCACCTGTGCTTTCCCAGATCCC
TGCAAGAATATATTTTCCAGGTTTTTTCTTACTTTCGAGGAGTAGAGGCACTGACAATGCCCTTGTTAAT
GTCTACCCAGTGGGGAAGATTAATAGCGCTGCACAGAGACCAACTTTATTACAAAGATTAATCCAGAGAC
CTTGGAGTATAATTAAGCAGGTTGATCTTTGCAACTATGTCTCTGTCAATGGGGCCACTGCTCACCCCTT
ATTGAAATGATGGAACCGTTTACAATATTGGTAATTGCTTGGAAAAAATTTTTCAATTGCCTACACAACA
TTGTAAGATCCTACCACTGCAAGCAGACAAGGGAAGATCCACAAGTAAGTCAGAGATCGCTGTACAATTC
TGCAGTGACCGGATTCAAGCCATCTTACGTTCATAGCCTTGGTCTGACTCTAACTATATCGTTTTGTGGA
GACACCAGTCAAAATTAACCTGTTCAAGTTCCTTCTCTTCATGGAGTCTTTGGGGAGCCAACTACGGGAT
TGTTTTGAGTCCAATGAAACCATGGGGTTTGGCTTCATATTGCTGACAAAAAGGAAAAAGTACCTAAATA
ATAAATACAGAACTTCTCCTTTTCAACTTTCTTCCATCACATCAACACCTATGAAGACAATGGGTTTCTG
ATTGTGATCTCTGCTGCCAAAAGTGACTTGAGTTTGTTTATAATTACTTATATTTAGCCAATTTACGTGG
AGAACTGGGAAAAGAGGTGAAAAAATGCCAGAAAGGCTCCTCAACCGAAGTTAGAGATATGTACTTCCTT
TGAATATTGACAAGGCTGACACAGGCAAGAATTCTAGTCACGCTCCCTCTTACCACAACTGCCACTGCAA
TTCTGTGCAGTGACGAGACTATCTGGCTGGAGCTCAGTTTTTTCAGGGCCTTCGTCGCTACTTGAGTTTC
AAATCAATTACCAGAAGTATTGTGGAAACCTTTACATATATGTGTATGGACTTGGCTTGAATCACTTTGT
TCCAGATAGGCCTGTAAGCTGAATGTCAAACTAAAGAAACTTGGGTTTGGCAAGAGCCTGATTCATACCT
ATCAGAACCCATCTTTGTTTCTCACCCAGATGCCTTGGAAGAAGATGATGGTGTAGTTCTGAGTGTGGTG
GTGAGCCCAGGAGCAGGACAAAAGCCTGCTTATCTCCTGATTCTGGAATGCCAAGGACTTAAGTGAAGCT
GCCTGGGCTGGAAGTGGGAGGATTAACATCCCTGTCACTCTTTCTATGGACTGTTCAAAATCTGAGAGCA
TACTCCAGCAAGATATAAGTTTTTGCAGCAAAACTGAGAAAATCAGCTTCAGGTCTGCAGTAAATCAAAT
TCTGTTCACTTTAGCCTGCTATATGTCATGGTTTTAACTTGCAGATGCGCCACACTCTGCAATGTTTACA
GAAAGCACTGAGTTGAGCAAGCAATTCCTTTATTTAAAAAAGTACGTATTTAGATAATCATACTTCCTCT
GTGAGATACTATAACTGAAAAACTCTTAAATATTTAGCAATCAAATAGGAAATGAATGTGGACTTACTAA
ATGGCTTTTAATTCCTATTATAAGAGCATATTTCTAGGTACCATCTGCTCCAATTATATTTCTTAACATT
TAAAACCAAAGTCCCTACACTTGATTTATATGGTATGTGCTTTGCTGAGCCAAGGAAGTATCATGCAATA
AGGCTTAATTACAAATGTCAAACCAGCTTTTCTCAAAACCAGGACTATCATCTAAGATTAATTACAGTAA

TTATTTTGCGTATACGTAACTGCTCAAAGGTTATGAATCTTATGAATGTTAACCTTTCCGTTTATTACAA
GCAAGTACTATTATTTCTGATTTTATAATAAGAAAATCTATGTTTAATCAACTGAGGCCTCTCAACCAAA
TAACATCTCAGATTAAGTTATATATTAAAGCTTATGTAACATAAAGCAAGTACATATAGTAGTGACTATA
TTTAAAAAAACAGAGAGCAAATGCTTAAAATGTAATATTTACTTAAATCAGATTATGATAATGTTGCAGG
ATTATACTTTATTGTATCTTTTTGTTTAATTGTATTTAAGCATTGTGCAATCACTTGGAAAATATTAAAT
TATTAACATTATGTATTAATATATTTTAAGCCTTTGTTTTTAAATTTTTTTGTTCCAGAGATTGTTTAAA
AATAAATATTGACAAAAAAAAAAAAAAAAAAAAAAGAAGATAGAGCGACAGGCAAGAGCACATGTAAACTGA
AACGAAGTACAAC

>@432ab81e-4271-46bf-b138-d710797bdbd1 runid=1a9a02
TTACGTATTGCTTTTCTGTTGGTGCTGATATTGCTGGGGCTTCATTCTGGCAGTTGGTGCCAGAACCGAT
CCTGAACTGGAAGAAACTATCCAGGTTGAGCATCCTGCTGGTGGTTACAAGAGAAACTGTTTGAAACTCG
TGGAGGAACTGTCCTCGCTCACAGCTCATGTAACAGGCAGGATCCCCTCTGGCTCACCGGCAGTCTCCTT
CGATGTGGGCCAGACTCTTTGAAGTTGGATCTGAGCCATTTTACCACCTGTTTGATGGGCAAGCCCTCCT
GCACAAGCTTGACTTTAAAGAAGGACATGTCACATAAATTACTGTGAAGGTTCATCCGCACTGATGCTTT
CATGTACGGGCAAATGACTGAGAAAAGGATCGTCATAACAGAATTTGGCACCTGTGCTTTCCCAGATCCC
TGCAAGAATATATTTTCCAGGTTTTTTCTTACTTTCGAGGAGTAGAGGCACTGACAATGCCCTTGTTAAT
GTCTACCCAGTGGGGAAGATTAATAGCGCTGCACAGAGACCAACTTTATTACAAAGATTAATCCAGAGAC
CTTGGAGTATAATTAAGCAGGTTGATCTTTGCAACTATGTCTCTGTCAATGGGGCCACTGCTCACCCCTT
ATTGAAATGATGGAACCGTTTACAATATTGGTAATTGCTTGGAAAAAATTTTTCAATTGCCTACACAACA
TTGTAAGATCCTACCACTGCAAGCAGACAAGGGAAGATCCACAAGTAAGTCAGAGATCGCTGTACAATTC
TGCAGTGACCGGATTCAAGCCATCTTACGTTCATAGCCTTGGTCTGACTCTAACTATATCGTTTTGTGGA
GACACCAGTCAAAATTAACCTGTTCAAGTTCCTTCTCTTCATGGAGTCTTTGGGGAGCCAACTACGGGAT
TGTTTTGAGTCCAATGAAACCATGGGGTTTGGCTTCATATTGCTGACAAAAAGGAAAAAGTACCTAAATA
ATAAATACAGAACTTCTCCTTTTCAACTTTCTTCCATCACATCAACACCTATGAAGACAATGGGTTTCTG
ATTGTGATCTCTGCTGCCAAAAGTGACTTGAGTTTGTTTATAATTACTTATATTTAGCCAATTTACGTGG
AGAACTGGGAAAAGAGGTGAAAAAATGCCAGAAAGGCTCCTCAACCGAAGTTAGAGATATGTACTTCCTT
TGAATATTGACAAGGCTGACACAGGCAAGAATTCTAGTCACGCTCCCTCTTACCACAACTGCCACTGCAA
TTCTGTGCAGTGACGAGACTATCTGGCTGGAGCTCAGTTTTTTCAGGGCCTTCGTCGCTACTTGAGTTTC
AAATCAATTACCAGAAGTATTGTGGAAACCTTTACATATATGTGTATGGACTTGGCTTGAATCACTTTGT
TCCAGATAGGCCTGTAAGCTGAATGTCAAACTAAAGAAACTTGGGTTTGGCAAGAGCCTGATTCATACCT
ATCAGAACCCATCTTTGTTTCTCACCCAGATGCCTTGGAAGAAGATGATGGTGTAGTTCTGAGTGTGGTG
GTGAGCCCAGGAGCAGGACAAAAGCCTGCTTATCTCCTGATTCTGGAATGCCAAGGACTTAAGTGAAGCT
GCCTGGGCTGGAAGTGGGAGGATTAACATCCCTGTCACTCTTTCTATGGACTGTTCAAAATCTGAGAGCA
TACTCCAGCAAGATATAAGTTTTTGCAGCAAAACTGAGAAAATCAGCTTCAGGTCTGCAGTAAATCAAAT
TCTGTTCACTTTAGCCTGCTATATGTCATGGTTTTAACTTGCAGATGCGCCACACTCTGCAATGTTTACA
GAAAGCACTGAGTTGAGCAAGCAATTCCTTTATTTAAAAAAGTACGTATTTAGATAATCATACTTCCTCT
GTGAGATACTATAACTGAAAAACTCTTAAATATTTAGCAATCAAATAGGAAATGAATGTGGACTTACTAA
ATGGCTTTTAATTCCTATTATAAGAGCATATTTCTAGGTACCATCTGCTCCAATTATATTTCTTAACATT
TAAAACCAAAGTCCCTACACTTGATTTATATGGTATGTGCTTTGCTGAGCCAAGGAAGTATCATGCAATA
AGGCTTAATTACAAATGTCAAACCAGCTTTTCTCAAAACCAGGACTATCATCTAAGATTAATTACAGTAA
TTATTTTGCGTATACGTAACTGCTCAAAGGTTATGAATCTTATGAATGTTAACCTTTCCGTTTATTACAA
GCAAGTACTATTATTTCTGATTTTATAATAAGAAAATCTATGTTTAATCAACTGAGGCCTCTCAACCAAA
TAACATCTCAGATTAAGTTATATATTAAAGCTTATGTAACATAAAGCAAGTACATATAGTAGTGACTATA
TTTAAAAAAACAGAGAGCAAATGCTTAAAATGTAATATTTACTTAAATCAGATTATGATAATGTTGCAGG
ATTATACTTTATTGTATCTTTTTGTTTAATTGTATTTAAGCATTGTGCAATCACTTGGAAAATATTAAAT
TATTAACATTATGTATTAATATATTTTAAGCCTTTGTTTTTAAATTTTTTTGTTCCAGAGATTGTTTAAA
AATAAATATTGACAAAAAAAAAAAAAAAAAAAAAAGAAGATAGAGCGACAGGCAAGAGCACATGTAAACTGA
AACGAAGTACAAC

>@c5597c60-f0ef-40e5-bd45-c0acf29eb582 runid=1a9a02
GTTGTGCTTGGACCAGTTGCATTGCTACTTTACCACTGTCATCTATCTTCTTTTTTTTTT
TTTTTTTATCACTTATAACTGTAAGTTTTTCAAGCAAAATCAAACATATTCTGCATTGTG
AGACAGAAGTATGCATTCTTTCATCCATCAGGCGGTCAGAGTCTTTGTTCTTGAAAAGGC
ATTGGTGTGTGTTGCTAGTTTTGAGATTTTTTTGTTTGGTTTTTGTTTTTTGTTTTGGTA
TTTTTTAGAAGGCCTTTCTTTGGAGCCCCTAAATTTTCTTCAAGGATGGTCCACATTTTA
CTGAAGAAAATTGATGGCCTAAGAGGTTATGTGACTTTCCCAGTACATAGCCAGTGAGCG
GCTGGCACCAGATGCTTTTGTATCACCTGGGAAATCACATAATCTTTAAACCGTATTCTC
TTCAGCAAAATGAACAAATCTTGAAAAAAATTAGGAGCCTAGATTATGTGACTTTCCAGT

GATACAAACTGTCTGTGCCTGTGTCAGTCTGGCTATCATGTATCACTGGAAGAGTCACGC
TGACACTCTTGAGCCATCAATTTTCTCTTCAGTAAGATGAGACCATCCTTGAAGAAAATT
TACAGGGCTCCAAAAGAAAGGCACTCTGAAAAACAAAGCAAAATAACTAAATCTCAAAAC
CATTTTCTGCAGAATGCAACTTTGAACTCTGCCTGTGAAAGAAAAAATCTTTAACATTTC
GTTCACGCGTAAATATGTTTTGATTTTGCAATTCATGTGAACAGGAAATGTCTGTTCTTT
TTCATCCTCAAACAATGAGAGTCTTTATTGTTCTGCAGAAAAGCATTAATTATTATTATT
ATTATTGGTTTTTATGATTTTTGTTTGGTTTTTGTTTTTGTTTTTCAGGTGCCTTCTTTG
GAGCCTAAATTTTCTTCAAGGATGGTCTCATTTACTAAATTCAATTATTTCTAAGAGGTT
ATGTGACTTTCCAGTGATGTACATGTATTGACTGACACAGGCGCAGATGGCTTGTATCGC
TGGGAAGTCACATAACCTCTTAGACCCTAAATTTCTTCAAGGATGTCTCATTTGCTGAAG
ATTGATGGCCTAGAGGTTATGTGACTTCCCATGATACAGGCTATCTGTACTGTGTCGGTC
ACTGGCTATGTATCTGCTGGGGAAGGTCACATAACCTCTTCAAACCATCAGTGCCTATAT
TCAGTAAATGGACCATCACTGAAGAAATTTAGGGCTCAAAGAGGTTATGTGACTTTCCCG
GTGATACATGGCCGGTGACGGCGCAGGCTGTGTCGCTGGAGGCCACGCTTAACCTGCAGG
CCATCGGTGTTTTTGTAACCAATAAAATGAACATCGCTGGCTAGGAGCTCAAAAAAAATG
AAAAAACTAAAACAAAAACAAATCTCAAAATCAATAATAATAGTATGTTTTTCTGCAAAT
AACTAAAAGACCTCGACCTACCACGAAGAATAACGTCGCTATTCGTAAAATATGTTTTTT
CAAGCAAATCATTATATTCTACATTATTGAGCAAACCTTGTATTCTTTTCTCACCATCCC
TCAGGCAGTCGAAGTCTTGTTTGTTCTGCTGAAAAACATTAATTATTATTATTATTGATT
TTTGGATTTTTGTTTGGTTTTTGTTTTTGTTTTCAGAGGCCTGCTTTAGACCTAGAATTT
TCTTCAAGGATGGTCTCATTTTACTGAAAGGTGATGGCCTAAGAGGTTGTGTGACTTTCC
CAGTATTATATGTGTCCAGTCACTCAGCTATATATCACTAGGAAAAATTATATCACATTA
TTCTTCCCAGGATGGTCTCATTTTGCGGAAGAAGAATTGATGGCCTAAGGGTTTAATGTG
TTTCCTTGGTGATACATGGCCAGTGACGGCAGGCACAGATGGCTTGCTGTGCTGGGAGAA
TCACCTGTTTGCAGGCCATCAGTACTACTCTTCAGCAGAACCATCATGAAAAATTTGGGA
GCCTAGACCCATGTGACTTTCCCAGTGATACAGCTATCTGTGCCATGTCAGTCACTGGCT
GTAGTATCACTGGGAAAGTCACTCAGCTAGGCCATCAATTTTCTCTTCAGTAAAATAGTG
TAAGACTCAAAACTCAAAAAACAAAACAACAAATCTCAAAATATAGTAATAATGATGTAT
TTTCTGTTTGTAAAACATGTTCACCTAAAAAATAAATCTTTACATCAAATATAATTTTTT
TCAAGCAAAATCAAAACATATTCTACGTGAACGAAATATGCATTCTTTCATCCCTCAGGC
AGTCAGAAGTCTTTTGTTGTTCTGCAGAAAAGCAGTAGTGTGTGTGTGGTTTGAGATTTT
TGTTTTAGTTTTTGTTTTTTTGTTTTCCAGGGCCTTTCTTTGGAGCCTAAATTTTCTTCA
GGATGGTCTCATTTTACTGAAGAAGAAGAATTGATAGCCTGAAGATTTATGTGACTTTCC
AGTGATACATAGCCGATGACTGACCCCACAGGCACAGAGTAGCTTG

>@00febf5a-3235-4a14-9cba-a4948eba0e5a runid=1a9a02
TCTGCAACGATCAACAAGCAAGCCAGAGATCGTTGTACAATTCCCCTGCGTGACCGAGGGATTCGCCATC
TTTACGTTCATAGCCTTGGTCTGACTCCCAACTATATCGTTTTTGTGGAGACACCAGTCGTACTCAATCC
TGTTCAAGTTCCTTTCATGGAGAGTCTTTGGGAGCCAACTACATGGATTGTTTTGAGTCCAATGAACCAT
GGGGGTTTGCCATATTGCTGACAAAAAGGAAAAGTAATTTCAATAATAATGACAGAACTTCTCTTTTCAA
CCTCTTCCATCACATCAACACCTATGAAGACAATGGGTTTCGATTGTGGATCTCTGCTGCTGGAAAGGAT
TTGAGTTTGTTTTATAATTACTTATATTTAGCCAATTTACGTGAGAACTGGGAAGAGGTGAAAAAAATGC
CAGAAAGGTTCCCAACCTGGAAGTTAGAGATATGTACTTCCTTTGAATATTGGACAAGGCTGACACAGGC
AAAGAATTTAGTCACGCCTCCCAATACAACGCCACTGCAATTCTGTGCAGTGACGAGACCATCTGGCTGA
GCCTGGAAGTCTCTTTCAGGGCCTCGTCAGTTACTTTGAGTTTCAAATCACCAATAGAAGTATTGTGGAA
ACCTACACATATGCGCATGGACTTTGGCTTGAATCACTTTGTTCCAGATAGGCTCTGCAAGCTGAATGTC
AAAACTAAAGAAACTTGGGTTTGGCAAGAGCCTGATTCATACCTATCAGAACCCATCTTTGTTTCTCACC
CAGATGCCTTGGAAGAAGATGATGGTGTATTCTGAGTGTGGTGGTGAGCTCAGGAGCAGACAAAAGCCTG
GCGATTCCGATTCTGAGAACGCTGGCGACTTAAGTAAGCTGCCTGGGCTGAAGTGGAGATTAACATCCCT
GTCACCTTCATGACTGTTCAAAATCTTGAGCATACTCCAGCAAGATATGTTTTGCAGCAAAACTGAGAAA
ATCAGCCTCAGGTCTGCAATCAAATTCTGTCTAATTTAGCCTGCTATATGTCATGGTTTTAATCTCTGCA
GATGCTAAAGCCAATTTGCAATGTTTTACAGAAAAAGCAATTGAGTTGAGCAAGCAATTCTTTATTTAAA
AAAAAAGACGTATTTAGATAATCATACTTCTCTGTGAGACAGGCCATAACTGAAAATCCTTAAATATTTA
GCAATCAAATAGGAAAATGAATGTGGACTTACTAAATGGCTTTTTAATTCCTACATAAGAGCATATTTAG
GCAATTTCATCTGCTCCAATTATATTTTTTAACATTTAAAACCAAAGTCTCTACACTTGATTTATATTAT
ATGTGGCTTTGCTGAGTCAAGGAAGTACCATGCAATAAGGCTTTTAATTACAAATGTCAAACCAAACTTT
TTCAAACCCAGTGGACTATCATCTAAGATTAATTACAGTAATTATTTTTATGTATACTGTAATCTGCTCA
AAGATTATGAATCTATGATCGCTAACCTTTCCGTTTATTACAAGCAAGTACTATTATTTGATTTTATAAT
AAGAAAATCTGAAGTGAATCAACTGAGGCCTCTCAACCAAATAACATCTCAGAGATTAAGTTATATACAA

AAGCTTATGTAACATAAAAAGCAAGTACATATAGTAGATGCGACTATATTTAAAAAAACAGCACAAAATG
CTTAAAAATGTAATATTTACTAAAATCAGATTATGGGATAATGTTGCAGGATTATACTTTATTGCATCCT
TTTTTTGTTTAATTGTATTTAAGCATTGTGCAATCACTTGGGAAAAATATTAAATTATTAACATTGAGTG
TATTAATACATTTTTAAGCCTTTTGTTTTTAAATTTTTCTTCCAGAGATTGCTTAAAAAATAAATATTGG
CAAAAAAAAAAAAAAAGAAGATAGAGCGACAGGCAAGTTTGAAGTACAATG

>@78289747-f173-41e0-87f8-f7745c4e11b3 runid=1a9a02
CCAAAGTCTCTACACTTTGATTTATATTATATGTGGCTTTGCTGAGTCAAGGAAGTATCATGCAATAAGG
CTTAATTACTAAATGTCAAACCAAACTTTTTCGCTAAACCAGGGACTATCATCTAAGATTAATTACAGTA
ATTATTTTGCGTGTATACGTAACTGCTCAAAGGTTATGAATCTTAAGTGAATGTTAACCTTCCGTTTATT
ACAAGCAAGTACTATTATTTCTGATTTTATAATAAGAAAATCTATGTTTAATCAACTGAGGCCTCAACCA
AATAACATCTCAGAGATTAAGTTATATATTAAAAGCTATGTAACATAAAGCAAGTACATATAGTAGTGAC
TATATTTTAAAAACAGAGCATAAAATGCTTAAAAATGTAATATTTACTAAAATCAGATTATGGGATAATG
TTGCAGGATTATACTTTATTGCATCTTTTTGTTTTTAATTGTATTTAAGCATTGTGCAATCACTTGGAAA
ATATTAAATTATTAACATTGAGGTATTAATATTATTTTAAGCCTTTTGTTTTTAAATTTTTTGTTCCAGA
GATTGTTTAAAAATAAATATTGACAAAATAAAAAAAAAAAAAAAAAACTAAAGAAAAAAAAAAAAAAAAAA
AGAGCAATACGTAACTGAACGAAGCACAAC

>@1fd7f69d-59f9-4bcd-80bc-05df9d905608 runid=1a9a02
CACGCTGTTCAGTTACGTATTACTTTTCTGTTGGTGCTACAGTATTTGCTGGGGGCTTCA
TTCTGCAGTTGGTGCCCAGAACTCTGGATCCTGAACTGGAAGAAAATGTCTATCCTCTGC
CTCTATCTCTCTGCGGACTTTGAGCATCAACATGGGCTTCTTCCTTATTCTTCCCACCAT
TTCAGGGTTGAGCATCCTGCTGGTGGTTACAGAAAACTGTTTGAAACTTGTGGAGGAACT
GTCCTCGCCGCTCTGGCGCAAGCCCAGCAACAGGCAGGATCCCCTCTGGCTCACCGGCAG
TCTCCTTCGATGTGGGCCAGGACTCTTTCAAGTTGGATCTGAGCCATTTTACCACCTGTT
TGATGGGCAGAAGCCCTCCTGCACAAGTTTGACTTTAAAGAAGGACATGTCACATGCACC
ACAGAAGGTTCATCCGCACTGATGCTTACATGCAGGCAATGACTGAGAAAGGATCGTCAT
AACAGAATTTGGCACCTGTGCTTTCCCAGATCCCCTGCAAGAATATATATTTTCCGGTTT
TTCTTACTTTCGAGGAGTAGGAGTTACTGACAATGCCCTTGGTGAAAATATCTGCAGTGG
GGGAAGATTTTCCTTGCACAGAGACCAACTTTATTACAAGATTAATCCAGGAGACCTTGG
AGACAATTAAGCAGGTTGATCTTTGCAACTAATAATCTCTGTCAATGGGGCCTCACTCAC
CCCATTGAAAATGATGGAACCGTTTACAATATTGGTAATTGCTTTGGAAAAATTTTCAAT
TGCTGGCATTGTAAAGATCCCACCACTGCAAGCAGACAAGGAAGATCAATAAGCAAGTCA
GAGATCGTTGTACAATTCCCCTGCAGTGACCGATTCAAGCCATCCTTACGTTCATAGTTT
TGGTCGACTCCCAGCTATATCGTTTTTGTGGAGACACCAGTCCAAAATTAACCTGTTCAA
GTTCCTTTCTTCATGGAGTCTTTGGGAGCCAACTTCATGGATTGTTTAGTCCAATGAGA
GCCATGGGGTTTGGCTTCATATTGCTGGCAAAAAAAAAAAGAAGATAAGCAATACGCAAC
T

>@8513c523-e8a1-44aa-a881-1a45b7f28a69 runid=1a9a02
GTAACTGCTCAAAGATTATGAATCTTGCGAATGTTAATCCTTCCAAGTTTATTACAAGCAAGTACTATTA
TTTCTGATTTTATAATAAGAAAATCTGTGTTAATCAACTGAGGCCTTCAACCAAATAATATCTTCAGAGA
TTAATATATAATGTATGCTTTATGTAACATAAAAGCAAGTACATATAGTAGTGACTATATTTAAAAAACA
GCATAAAAATGCTTAAAATGTAATATTTACTAAAAATCAGATTATGGGATAATGTTGCAGGATTATACTC
TTATTGCATCTTTTGTTTAATTGTATTTACACAGTAACCACTTGGGAAAATATTAAATTATTAACATTGA
GGTATTAATACAAAAAAAAAAAAAAACAAACAAAAAAAAAAAAAAAAAAAGAGCAATACGTAACTGAACGAA
GCACAATA

>@fa09503d-61eb-4954-bc72-ce29cf95b8a1 runid=1a9a02
ATTTAGATAATCATACTTCTCTGTGAGACAGGCCATAACTGAAAACTCTTAAATATTTAGCAATCAAATA
GGAAATGAATGTGGACTTACTAAATGGCTTTTAAGACTCCTATTATAAGAGCATATTTTAGGTACCATCC
GTCTAATTATATTTTTAACATTTAAAAACCAAAGTCCTCTACACTTGATTTATATTATATGTGGCTTGCT
GAGTCAAGGAAGCATCATGCAATAAGGCTTAATTACTAAATGTCAAACCAAACTTTTCTCAAACCAGGGA
CTATCATCTAAGATTAATTACAGTAATTATTTTGCGTATACGTAACTGCTCAAAGATTATGAATCTTATG
AATGTTAACCTTTCCGTTTATTACAAGCAAGTACTATTTCTGATTTTATAATAAGAAAATCTGGTGTTTA
ATCAACTGAGGCCTCCTCAACCAAATAACATCTCAGAGATTAAGTTATATATTATAGTGTTTATGTAACA
TAAAAGCAAGTACATATAGTAGTGACTATATTTAAAAAAAAAAAAAAAACAGCATAAAATGCTTAAAAATG
TAATATTTACTAAAATCAGATTATGGATATCGTTGCAGGATTATACTTTATTGCATCTTTTTGTTTAATT
GTATTTCTAAGTATCAGCAATCACTTCTGGGAAAATATTAAATTATTAACATTGACGAAAAAAAAAAAGA

AGATAGAGCGACAGGCAAGCAGCAATACGTAACTGAACGAAGCACTACCG

>@2de15eb7-5d94-42ec-b6bc-092168123b83 runid=1a9a02
AGCGACAGACACATCTATTCATTCCGCTCCAGATCTTTGATATCAGACGGGTCACTTGTTTAACACCTTG
AAGAAAAAGAAATTTAGCCCCAGCAGTTGTATCGGCAACACCAACGCCAATACGATCGACAGATCATCGC
TTAATAATTAAACAAAAAGACGTACGAGCGCAACCGTTCCGATTTGCAATACGCATATTTATGCTGTTTT
TTGCCGATAGTCACTACTATATACCAGCGCAGCAGCTACTTTTGAATATGACCATCTTTCTGAGATGCTT
GCTGGCGGTATTCTGCTGACAAACGGCTTATTTTTCTGGCGTAATCAGAAATACAGCGTTTTACAATAAA
CGGAGCGTTAACACCATAAGACATATCTTTTGGAGTTGTTGATGATACGCAACAACACCACTATCTTTAG
ATGTCCTGAGCTGAAAGCTTGTTTGACATTTGTACTGGTTTTACAGGCGATGGTTTCTGACTTCAGCGTG
CCACATAACAAATCGCGAGGACTTTGTTTTTAATGCTGTAAACAGGTCGGGCAGACAGTACCATGCTCTA
ACAAATGCCATTTAGTGTCCACACCGCTTCCTGCTTGACACAACAGCTTACAGCTTCACAGTGCCTGTCT
CATTGGTAGAAGTGGCGACACCAATGAATGCACTTTTTTTAAACAAAGGCTGTTAATCAGTGTTTTCGTA
ACACAGCCAGGTGTATCTGCAGTTGAAACTGGTTTATATGGCAGGTTGGGCTGAACAGACCTTCTGATTG
AACGGCTGATTTTTCAAAGCACACAAAAACATATCTTTTGTATGCTCGCATTTGGAACAAACCATGAGGT
GTATTCATTCGGGCCAGTCTCATATCAAACACCAGAACACATCACTTCTCAGTACCTGGGCGGTCGGTAC
ATGGTTCCCGGCGGAACCCAGCCTTTAGTTTGACATCGCTGGCAGCCTGGTTCAACAAAGTGACCAAGCC
AGTCCATACGCGTGTGGCTTTCCCCACAACAGCTTCTGGTATGAGTGAAATACAGCCTGGACGAGGCCCC
AGTTCTCTTCAGGGGTCTCATGTCAGCATTTGAGTTTCTCAAATCAATTACCAGAAGTATTGTGGGAAAC
CTTACACATATGCGCATGGATTCTTGGCTAGCTTTGAATCACTTGTTCCAGATAGGCTCTGTAAGCTGAA
TGTCAAAACTAAAGAAACTTTTGGGTTTGGTAAGAGCCTGATTCATACCCATCAGGAACCCATCTTTGTT
TCTCACCCAGATGCCTTGGAAGAAGATGATGGTGTAGAAGTTCTGAGTGTGGTGGTGAGCTCCAGGAGCA
GGACAAAAGCCTGCTTATCTCCCTGATTCTGAATGCTAAGGACTTAAGTGAAGTTGCTCCGGCTGAAGTG
GAGATTAACATCCTGTCACCTTCTCATGGACTGTCTTAAAAATCTTGAGCATGTCTCCAGCAAGATATGT
TTTTGGTAGCAAAACTGAGAAATCAGCTTCAGGTCTGCAATCAAATTCTTCTGTTCAATTTTGTTCGCTA
TATGTCATGGTTTTAACTTGTGATGCGCACAATTTTGCAATGTTTGCCAGAAAGCACTGGAGTTGAGCAG
CTAATTCCTTTTTATTTAAAAAAAAAGTACGTATTTAGATAATCATACTTCCTCTGTGAGACAGGCCATA
ACTGAAAAACTCTTTAAATATTTAGCAATCAAACAGGAAAAATGAATGTGGACTCACTTAAATGGCTTTA
ATTCTATTATAATATATTTTAGGTACCTATCTGTTCCAATTATATTTTTAACATTTAAAACCAAAGTCCT
CTACACTTGATTTATATTATATGTGGCTTTGCTGAGTCAAGTGAAGCATCATGCAACAAGGCTTTTAATT
ACTAAATGTCAAACAAACTTTTCTCAAACCAGGGACTATCATCTAAGATTAATTACAGTAATTATTTTAT
GTATACGTAACTGCAGCCAAAGATTATGAATCTTGCGAATGTTAACCTTCCGTTTATTACAAGCAAGTAC
TATTGGTTTCTGATTTTATAATAAGAAAATCTGTGTTTAATCAACTGAGGCCTTCAACCAAATAACATCT
CAGAGATTAAGTTATGTTATTAAAAGCTTATGTAATTAAAAGGGGGTACATATAGTAGTGACTATATTTA
AAAAACAGCATAAATGCTTAAAATGTAATATTTACTAAAAATCAGATTATGGGATAATGTTGCAGGATTA
TACTTTGTATTGCATCTTTTGTTTAATTGTATTTAAGCACAGTAATCACTTGGGAAAATATTAAATTACA
ACATTGAGGTATTAATACATTTTAAGCCTTTTGTTTTAAATTTTTCTTCCAGAGATTGTTTAAAAATAAA
TATTGACAAAAAAAAAAAAAAAAAAAAAAAAAAAAAAAATAAACAAAAAAAAAAAAAAAAAAGAAGATAGA
GCTGACAGGCAAGTAGCAATACGTAACTGAACGAAGCACAAC

>@04640489-b7c7-43e4-aa1a-c5fff3c6bbe3 runid=1a9a02
CGCTTAATTCCCAAACGACAAACAACCTGTTATTTCATAGAAGCACTATAGAAAAAGAAACTACAGCAAA
AGAGTGTACCCAGAATTCTCCATTCATTCTTCTATTGCCAGGTTCATTGTTACCAATTACAGAATTATTT
GAATGTATACAGATTTTGTTTAGTATTATTTAGCAGTTCTCAAGTAGTCCTTGGACCAAGACCATCACCT
AGGAAACTGTTAGAAATACAATCTTGTTTTGTCCTGGGCACTGAATGAGAAATTTTGGGAGTGGAGCCTG
CAATCAGTATTTTAAGAAACTCTTATGTGATTCTGATGCACACTCAAAGTTTGAGACCATTTTCCACTTA
CTAGTGAGTAAATTTTAATGCATAAGAATTACTGTACAGGGCTATTGACACAAACCCATCTAAACTACAT
CCTTTAAGCATCTTAAGATGCTGCAAAAGGCATTTTGACCATTCTGAGGTTTCTTCCCTACTCCCTGACT
TAAAAACCCACTCTCCAGCTATTGCAAGATGACTCACTCATACCTCACTACAGTTACAACCTTACTTGAG
TGCACGTTTCAATCTGCTGCTATTTGGATTTTCTAAGATTTTGCAGCTTCACTACGAAATTTCACTAAGA
AGTGTAAATGATGGAGAAAATGAAAATAACCCCTCTAAAACAATCAAAATGTGTTTCTTTGCCTGTATAA
GCTTTCGTTCTAAATGCTTTGTATTAAAAATAAGAGGCTGTCTGGTAAAGCCTTTAAAACCACTTGATTT
CAGACAAGGAAGATCCAATAAGTCAGAGATCGTTGTACAATTTCTGCAGTGACCGATCTGGCTTCATCTT
ACGTTCATAGGTAATTTGAAGGCCTGCTATAATCTTCAGGAAACTCAAGTTTAAAAGATTTGCTTTGCCT
ACCTTTGATACCAATCCTGGATCACAATATTTTTTGCAGAAAACTAAATTTAAACATAATTAATTTCATG
TAGGCACTGTTGACTTGTAACTGAGCATACCACATATATACACAATTTGTTCAGGGCTTGAGAATCAGCC
TTTCATTCACAAGCCCATTTGTGTTTCTGGAACAGTTTTGGTCTGACTCCCAACTATATCGTTTCCTTGT
GGAGACACTGAATAAAATTAACCTGTTCAAGTTCCTTCTCTTCATGGAGTCTTTGGGAGCCAACTACATG
GATTGTTTTGAGTCCAATGAAACACATGGGGTTTGGCTTCATATTGCTGACAAAAGAAAAAAGTACCTCA

ATAATAAATACAGAACTTCTCCTTCTTTTCAACCTCTTCCATCACATCAACACCTATGAAGACAATGGGT
TTGATTGTGGATCTCTGCTGCTGGAAAGGATTTGAGTTTGTTTATAATTACTTATATTTAGCCAATTTAC
GTGAGAACTGGGAAGAGGTAAAAAATGCTTGAAAGGCTCTAACCTGAAGTTAGGAGATAGTACTTTGAAT
ATTGATGGGCACTGACACAGGTGTAATTAGTCACGCTCTAATACAACTGCCACTGCAATTCTGTGCAGTG
ACGAGAATTGTCTGGCTGGAGTCGCCTGAGCCTTTTCAGGTCCCTGTCAAGCATTTGAGTTTCCTCAAAT
CAACAATTACTAGAAGTATTGTGGGAAAACCTTACACATATGCAAAAAGGATCTGCTTGAATCACTTTGT
TCCAGATAGGCTCTAAGCTGAATGTCAGCAAAACTAAAGAAACTTGGGCTTTGGTAAGAGTCACATTCAT
ACTCATCAGAACCCATTCTGTGCTTCTCACCCAGATGCCTTGGGAAGCGATGGTGTAGTTCTGAGTGTGG
TGTGATCTTGAGAGTTAAGGACAAAAGCCTGCTTATCTCCCCATTCTGAATGCCAAGGACTTCTATGTAG
TTGCCTGGCTGAAGTGGAGATTAATGATCCCTGTCACCTTTCATGGACTGTTCAAAAAAATCTTGAGCAT
ACCTAGACAAAGATGTTTTTGCAGCAAACTGAGAAAATCAGCCTGTACAGTCTAAAATCAAATTCTGTCT
AATTTAGCCTGCATATATGTCATGGTTTTAATCTCGTGATGCAATAATTTTGCAATGTTTTACAGAAAGC
ACTGAGTTGGAGCAAGCAATTCTTTATTTAAAAAAAAAAAAAAAAAGAAAAAAAGAAAGATAGAGCGACAG
GCAAGTAGCAATACGTAACTGACGAAGTACAAC

>@8429b8de-c612-4dca-b744-0ff3e7093ccf runid=1a9a02
GTTGGCATGCTTCCGTTCAGTTACGTATTACTTTCTGTTGGTGCTGATATTGCTGGGCTC
TTCATTCTGCAGTTGGTGCCAAGAACTCTGGATCTGAACTGGAAAATGTCTATCAGGTTG
AGCATCCTGCTGGTGGTTACAAGAAACTGTTTGAAACTTTGTGGAGGAACTGTCCTCGCC
GCTCACAGCTCATGCCAACAGGCAGGTCCCCTCTGGCTCACCGGCAGTCTCCTTCGATAT
GGGCCAGGACTCTTTGAAGTTGGATCTGAGCCATTTTTACCACCTGTTTGAGTGGGCAAG
CCCTCACCACGCAAGTTTGACTTTAAGAAAGGACATGTCACACTTCTAGAAAGGTTCATC
CGCACTGATGCTTACGTACGGGCAGCTGACGAAAGGATCGTCATAGCAAATTTGGCACCT
GTGGCCAGATCCTGCAGAGCCATATTTTCAGGTTTTTTTCTTACTTTCGAGGAGTAGAGG
TTACTGACAATGCCCTTGTTAATGTCTACCCAGTGGGGAAGATTACTACGCTTGCACAGA
GACCAACTTTATTACAAAAGATTAATCAGAGACCTTGGAGACAGTGCAGGTTGATCTTTG
CAACTATGTCTCTGTCAATGGGGCTGCTCACCCCACGTGAAAATGATGGAACCGTTTACA
ATATTGGTAATTGCTTTGGAAAAAATTTTTCAATTGCCTGCAACATTGTAAAGATCCCAC
CTGCAAGCAGACAAGGGAGAGATCCAATAAGCAAGTCAGAGATCGTTGTACAATTCCCCT
GCAGTGACCGATTCAAGCCATCTTACGTTCATAGTTTTTGGTCTGACTCCCAACTATATC
GTTTTTATTGGAGACACCAGTCAAAATTAACCTGTTCAAGGTTCAACTTTCTTCATGGAG
TCTTTGGGAGCCAACTGCATGGATTGTTTTTTATCAATGAAACCATGGGGTTTTGAGAAG
CTCATATTGCTGACAAAAAAGGAAAAGTACCTCAATAATAATGCAGAACTTCTCCTTTCA
ACCTCTTCCATCACATCAGCACACCTATGAAGACAATGGGTTTCTGATTGTGAATCTCTG
CTGCTGGAAAGGATTTGAGTTTGTTTATAATTACTTATATTTAGCCAATTTACGTGAGAA
CTGGGAAGAGGTGAAAAAATGCCAGAAAGGCTCCCCCAACCTGAAGTTAGGAGATATGTA
CTTCTTTGAATATTGACCACAAAGGCTGACACAGGCAAGAATTTAGTCACGCTCCCAGCC
ACAACTGCCACTGCACAATTCTGTGCAGTGACGAGACTAACTCTGGCTGGAGCCTGAAGT
TCTCTTTCAGGGCCTCGTCAAGCATTTGAGTTTCCTCAAATCCAATTACCAGAGAAATGT
AAGGAAACCCCTTACGCATATGCGTATGGACTTGGCAGATCACTTTGTTCCAGATGGGCT
CTGTAAGCTGAATGTCAAAACTAAAGAAACTGGGTTTGGCAAAGGCCGCAGTTCATACCC
GCATCAGAACCCATCTTTGTTTCTCACTTAGATGCCTTAAGGAAGAAGATGATGTGGTTC
TGAGTGTGGTAGGTGAGCCCAGGAGCAGGACAAAAAGCCTGCTTATCTCCTGATTCAGAT
ACCAAAGGACTTAAGTGAAGTTGCCCGGGCTGAAGTGGAGATTAACATCCTGTCACCTTT
CATGGACTGTTCAAAATCTTGAGCATACTCCAGCAAGATATGTGTTTTTGGTAGCAAAAC
TGGGAAAATCAGCTTCAGGTCTGCAATCAAATTCTGTTCAATTTTAGCCTGCTATATGTC
ATGGTTTTTAACTTGCAGATGCGCACAATTTTGCAATGTTTACAGAAGCACTGAGTTGAG
CAAGCAGAATTCCTTTATTTAAAAAAAAAAGTACGCTATTTAGATAATCCTTACTTCTCT
GTGAGACAGGCATAACTGAAAAACTCTTAAATATTTGCTTAATCAAATAGAAATGAATGT
GGACTTACTAAATGGCTTTTAATTCCTATTATAAGAGCATATTTTAGGTACCTATCTGCT
CAGTATATTTTAACATTTAAAAACCCAAAGTCCTCTACATTACTTGATTTATATTATATG
TGGCTTTGCTGAGTCAAGGAAGTATCATGCAATAAGGCTTAATTATAAAATGTCAAACCA
AACTTTTTCTCTAAATGGGGTTATCATCTAAGATTAGTACAGTAATTATTTTGCGTATAC
GTAACTGCTCAAGGTTATGAATCTTATGAATGTTAACCTTTCCGTTTATTACAAGCAAAT
TACTAATATTTCTGATTTATAATAAGAGAAAATCTATGTTTAATCAACTGAACCTCTCAA
CCAAATAACATCTCAGAATTAAGTTATATATTAAAAAGCATGTAACATAAAAGCAAGTAC
ATATAGTAGTGACTATATTTAAAAAATAGAGCATAAAATGCTTAAAAATGTAATGTTACT
AAAATCAGATTATGGGATAATGTTGCGAGTTATACTTTATTGCATCTTTTGTTTAATTGA
ATTTAAGCATTGTGCAATCACTTGGGGAAAAATATTAAATTATTAACATTGAGGAGTATT

AATACATT

>@abfead6d-c832-454b-bc5e-406773fd12bf runid=1a9a02
GTTACGTATTGCTTAGCAGAGAGGTGGGAGTTCAAGACCAGCCTACTAACATGGAAAACCTCATCTTTAC
TAAAAACACAGACTAGCTGGGCATGGTGGTGCATGCCTGTAATCCCAGCTACTCAGGAGGCTGAAGCAAG
AGAATCGCTTGAACCCAGGAGGCAGAGGTTGTAGTGAGCCGAGATCATACATATTGCACTCCAGCCTGGG
CAACAAGAGGAAATTCCATCATAAATAAATAAATAAAATAAATAAATAAATAAATAAATAAATAGAAGTA
ACAGGAGCTAGCTTGTAGAAAAAGAATAAATTTTACTTATAGATGTAATTTGAAAGTACCCACTAGCAAT
CTTAGCAAAGCATTCCATAGGCAGCTATTGCACATGGATCTGGAGCTCCAAAGAAAGATCATGGAGAGAG
AGATCAATCTGGGTACCTTAAGGATTTTTGCTTTCCCCTTACAGCTATGTTATCACTGACATGTAGTTGA
GTATTCTCAAAATACTCACCAAATGGATGAGTCAGTTAATTTCAGTAAACTTTACTGCTAATAATGGTAA
AATCACACCATCTTTAAGGAAATAAAGAGGATCACTTAACTTAAATATTCACACTGCAAAAAAAAAAAAA
AGAAGATAGAGCGACAGGCAAGTAGCAATACGTAACCGAACGAAGCACATCG

>@1f4c3f6f-ea58-475e-89ba-9e96a70c93c4 runid=1a9a02
GTTTATCTTTTTCTTGCGATAGTTAGCTTGTCAATATTTATTTAAACACTTGAACAAAAGAAATTTGAAA
CAAAAGGCTTAAAATGGCTATCATCGTGCCAATACTTAATATGATGCACAATGCTTAAACATAATAAACA
AAGAGCGCACAAAAACAGGTCCCGTGCAAGATTAACCATGGTCGATTTTAAATATTACATTATTTTGATG
TTTCCGTTTTTTGTCATAAGTCACTGGCCATATGTAGCCAGCTCATGTTACATGCTTTTACCAGTGGTCT
CAGCCTCGAGATGTTATTTAATGAGGTTCAGTTGATGTGATTTTCATTATAAAATCGCAGTCAACAGTAC
ATTGAACGGAGTAAGACATGTTTTTTGAGCAGTTGCGTGATACGCAAAGTCGGTTGGTCGGGTGGCTCAA
TTCAGATAGTCTCGGTTTGGCAAAGCATGGACATTTGCGTACTGCCTACGCATGATACTTCAGTAAAGCC
ACGCGTGGGAGACTTTGCTTTAAATGTTAAAAATACTGGCAGAGGCACTAAAATACAAGCCTATCAGAAT
GCCATTTAGTGTCCAATATCATTTTCCAGTTGACTGTTAAATATTTAAGAGTTTTCACAGCGGCTCGTTC
TCAGAGTGCAAAGTTAGCGATTACCCAAATACTTTTTCTTTAAATGGTGGAATGAAGCTGTCCCCAGCTT
TCAAAACACAAACAGGTGCATCTGCAGTGCCAAACCACATATAAGCAGCCGAACAGTTCTGATTGTGTAC
TGGATTTTCAGCTCTGTTGTCAGTGCCCAGCTGATCGCAGCCTCGCCCGGCAACCACTCTGCCCGGCACA
GCTTGTTCTTTCCTGTCATCGTCAGCCGTTCAAGGAGTCTGCGAGAACAGTTATTTTGGTTCTGATGGGT
GGCGAATCAGGGTCGCCTTTAGTTTGACATTCAAAGCACAGAGCCTGGTTCGGAACGCGAGATTCGCCGT
CCATACGCACATAAGGTTTCACACAGCTTCTGGTAATAGGCCAGATAAACCAAAAACAGCCGGCACAGCG
GCAGCTGTATTAGAGCGTGACTAGCTCCAGCCTGTATCAGTCTTGAACAATACCAAAAGGAAGCACATAT
CTCCTGATCCAGCTGGGAGTTTGGTTTTTTTTCGCTCTTCAGCCTCACGGGTAACAGCCGAATATGGGCG
GTTTATAAACAGTTCTCGTTCAGCAGCAGAACCATCAATCAGAAACTCGCCAGCTAGGTGCCGATGATGG
AAGAGGAGGCTTCTTTCCTTGCATCGTTGGTGAAATTTCATTATTGACTCGTGCCTGTCCATGTGCCGCT
CCACTCCATGAAATAGTGAATGAACGTGTTAATCATGTCTCCACAAAAACGAGCTATGTTGGGGGAGTCA
GACCAAACTATGAACGTAAGACGCTTGAATGTCAGCCGTAGCTAACGATTTGACTTGCCATCTTCGTAGT
GCGGGATCTGCCACTTTTCCAGTACCAAACGGTTCCATCATTTCAGCGTGGGGTGAGTTAGTCTCACGGC
ACATACGTAAAGATCAACCTGTTGTTCAATCTCTGGACAATCTGCAGCCAGTTGGTCTCAAAAAAAGCGT
GTATCTTTCCTCATTCGGGTACATTGTCGCGACCTCCGAAAGTAAGAAACTCAGTCAGGCTGTAGGATTC
GGGAGGCACAGGTGCCGCCTATCCGTCATTGCTCGGCAAAGATCAGCGAATTTTCTGTGACATGCCTTTC
TTGAACTTTGCAGGTATCAAACAGGTGGTGGTCGGTCAGATCCGTCCAGTTCTATCGAGCGAGGGGACCG
GCCCAGCGGCGACAAAGTTCAAACAGTTTCAGGACCTCACTGTGCCAGTGATGCTCACTTGACAGACATT
TTTCTTCCATGCCCAGATCCAGAGCCCTGGGCCGAGAACTCCTTCATTCCGCAGCTGCGCCAGAACCTGG
ATCCCGAACTGGAAGAAAATGTCTATCCAGGTTGAGCATCCTGGCTGGTGGTTACAAGAAACTGTTTGAA
ACTGTGGAGGAACTGTCCTGCCGCTCACAGCTCATGTAACAGGCAGGATCCCCCTCTGGCTCACCGGCAG
TCCTTTCGATGTGGGCCAGGACTCTTTGAAGCTGGATTCGAGCCATTTTACCTGTTTGATGGGCAAGCTC
CTGCACACAAGTTTGACTTGAAGAAGGACATGTCACATACCACAGAAGGTTCATCCGCACTGATGCTTAC
GTACGGGGCGACTGAGAGTTGATCGTCATAACAGAATTCTGGCACCTGTGCTTTCCCAGATCCCTGCAAG
AATATATTTTCCAGGTTTTTTTTCTTACTTTCGAGGAGTAGAGGTTACTGACAATGCCCTTTGTTAATGT
CTACCAGTGGGGAAGATTAATATGCTTTCTGCACAGAGACCAACTTTATTACAAAGAAAGATTAATCCAG
AGACCTTTGGAGACAATTACAGGCTGATCTTTTGCAACTATAAATCTCTGTCAATGGGGCCACTGCTCAC
CCCCCACATTGAAAATGATGGAACCGTTTACATAATATTGGTAATTGCTTTGGAAAAATCTTTCAATTGC
TCACAACATTGTAAAGATCCCACCACTGCGCTGGACAAGGAAGATCCAATAAGTGTCAGAGAGATCGTTG
TACAATTCCCTGCAGACCGATTCAAGCCATCTACGTTCATAGTTTTGGTCTGACTCAACTATATCTGCTT
TTTGTGGAGACACTAGTCAAAATTAACCTGTTCAAGTTCCTTTCTTCATGGAGTCTTGGGGAGCCACATG
GATTGTTTTGAGTCCATAATGAAACCATGGGGGGGTTTGGCTTCATATTACTGACAAAAAGGAAAAAAGT
ACCTCAATAATAAATACAGAACTTCTCCTTTCAACCTCTTCCATCAAGACATCAACACCTATGAAGACAA
TGGGTTTCTGATTGTGGATCTCTGCTGCTGGAAAGGATTTGAGTTTGTTTATAATACTTATATTTAAATA
ATTTACGTGAGAACTGGGAAGAGGTGGAAAATGCCAGAAAGGCTCCCCAACCTGAAGTTAGAGATATGTA

CTTCCTTTGAATATTGACAAGGCTGACACAGGCAAGAATTTAGTCACGCTCCCATCACAACTGCCACTGC
AATTCTGCAGTGACGAGACCATCTGGCTGGAGCCTGAAGTTCTCTTTCAGGGCCTCGTCGAAGCATTTGA
GTTTCCTCAAACTACTACCAGAAGTATTGTGTCCTTACACATATGCGTATGGACTTGGCTTGAATCACTA
TTCCAGATAGGCTCTGTAAGCTGAATGTCAAACTAAAGAAACTTGGGTTTGGCAAGAGCCTGATTCACTC
CATCGAACCCATCTTGTTTCTCACCCAGATGCTCTTGGAAGAAGATGGTGTAGTTCTGAGGTGGTGGTGA
GGCCCAGAGCAGGACAAAAGCCTGCTTATCTCCTGATTCTGAATGCCAAGGATTTTAAGTGAAGTTGCCT
GGCTGAAGTGGAGATTAACATCCCTGTCACCTTTCATGGACTGTTCAAAAAATCTTGAGCATACTCCAGC
AAGATATGTTTTTGTAGCAAAACTGAGAAAATCAGCTTCAGGTCTGCAATCAAATTCTGTTCAATTTTAG
CCTGCTATATGTCATGGTTTTAACTTGGCAGATGCGCACAATTTTGCAAATGTTTTACAGAAAGCACTGA
GTTGAGCAAGCAATTCTTTATTTAAAAAGCATGTATTTAGATAATCATACCCTCTGTGAGACAGGGCCAT
AACTGAAAACTCTTAAAATATTTAGCAATCAAATAGGAAATGAATGTGGACTTACTAAATGGCTTTTAAT
TCTATTATAAGAGCATATTTAGGTACCTATCTGCTCCAATTATATTTTTAACATTTAAAACCAAAGTCCT
CACACTTGATTTATATTATATGTGGCTTTGCTGAGTCAAGGAAGTATTGGCGCAATAAGGCTAATTACTA
AATGTCAAACCAAACTTTTCTCAAACCAGGGACTATCATCTAAGATTAATTACAGTAATTATTTTGCGTA
TACGTAACTGCTCAAAGGTTATGAATCTTAAGTGAATGTTAACCTTCTGTTTATTCATAAGTAAGTACTA
TTATTTCTCTGATTTTATAATAAGAAAATCTATGTTTAATCAACTGAGGCCTTCAACCAAATAACATCTC
AGAGATTAAGTTATATATTAAAAGCTTATGTAACATAAAGTAGTACATATAGTAGTGACTATATTTAAAA
AACAGCATAAAAATGCTTAAAATGTAATATTTACTAAAATCAGATTATGGGATAATAAGTTGCAGGATTA
TACTTATTGCATCTTTGTTTGACTGTATTTAAGCATTGTGCAATCACTTGGGAAAAAATATTAAATTATT
AACACAGGTATTAATACATTTTAAGCCTTTTGTTTTAAAATTTCTTTGTCTCCAGGTGAGATTGTTTAAA
ACAACATTACAAAAATAATGTTTTATATCTTAAAAAAAAAAAAAAAAACAAAAAAAAAAAGAAGATAAAT
ATGAGGCGTAGCAATACGTAACTGAACGAAGCACACTG

>@a2cb22a7-59ce-45c1-a0aa-64875b46b05a runid=1a9a02
AAAATGCTTAAAAATGTACATTTTACTAAAATCAGATTATGGGATAATGCTGCAGGATTATACTTTACAT
CTTTTGTTTAATTGTATTTAAGCATTGTGCAATCACTTGGGAAAAATATTAAATTATTAACATTGGAGGT
ATTAATACATTTTAAGCCTTGTTTTTAAATTTTTGTTCCAGAGATTGTTTAAAAACAAATATTGACAAAA
ATAATGTTTTATATCTTAATTCTAGTATCTGTTTTATGCTTGAAAGCATTACAGATCATGATACCTAAGA
TGTATCAGCATGGTGTTTGACTAGTAAAATAGGCTGAAGGAACAGACTGACCAGGTACCTGATGTATCCA
TTTGATCTTTATTTAGATAAGATGGAAACTCATTTTTAAATTACAAATAATTCAGTAAAGCATACTTTTC
GAAGAATAACTAAAAATACAAAAAGTAGAAATAAAGTAAAATCATACAGAGCTCATCTAAGTTCAACTGC
TCTTAAGATTTGGGTACTGGTCTTTTCTTCTTTCTATTCCAGATTTTTTTTATGTAATCACTATTTTTC
CATCAGTAATTACTAGATAGAATCCAAAATCTCAAAACAGAAGAGAATGCTAGGAATCCGTAAGGAATAT
GTCACTGTAGATAAATATCAATCCATTGGTTCCTGTCTCTAGACATGATATATGAACAGAATTCATTAAA
GAAACTATTTTTCTATTTTTAAAAAATGAAAATTAATTACTGAAAATTACTAAGTTTTAGACTTAATGTC
TTCAAATTAAAAAAAAAAAATTCCTACAATTCCACTTCCTACCCTTGCCAATCAATGATTCTCCCTGGCTT
CTTCCTATCTACATTTTTTATGGAAGCAAGTCTCTGCAATTCAAGGTGACCATATCTCCTGCTGTACGAT
CCCTTTAGAGATCACCAATGAAGAAAGGAAAGAGGCAACCTACGATAATATCGGCTAATACCATTTCACT
CATCATTGCATTCCTAATTACAATTATCACTACAATTAGAGCAAGGAGACTTAGGATTACTTTTCAAGTG
GCAAGATAGAATATGTTTTTAAAACTTAAATTAGAATAAGAGATATTTAAGTTATGAGAGATTAACTCGT
AAATTCGGACTTTCTAGGAAACACCTACTCCTCTGAGCGATCTAGAGAATTATGTCCATCTATACAAAAG
AAAATCTGTTTTCAGAAAAATAACTCAATTGAATTAATATAAATTAACCTGTTAATAAGTAGTTATTTTT
AACAACAAGCTGTTTGAAGCCTATTTTTTTTCTTCCATTTTTGTGCCTTATATTTGCTAATACAGCCAAGA
CGAAAAGATAATAAAATCTTTGACATAATATCATCAAGGGGTAATCAGTAACTGATAATCGCCTCATGGC
AGATAATCTAGACAGTCCCTGTCCTATATTCTAAAGCAGAAAAGACCAACAGACATCAAAGGGAGTCTGT
CTTCCAAGGATTCTATGGCAGTATACTCCATCATCCCACAGTCTGGCTGAGAGCTAAATGTGTATTTTCT
TCTGTTCAGCCTAATAGGAAGAAAGTATCAATGATTAGAGATTATAAACATATCCCTAAGGAATACTAAA
TCTGGTACAATGCAATCTCAATGAGAAAAACAATCTCTGGAAAGACAAGAGAAAAAAAAAAACCCATTTA
TCGAGTGCCTACTTCATTTACTGCATTTTTATTCCTCATACTACACCCATCATGGTACTATTTTCTACCA
TTTTAAAGATGAGGAAAGGAGGCTAAGTTACGTTCCTAATGAGCAATAAACCAGTTTTGTAGAATTCTGT
GAATTCTTACATTAGGAAATAGATAATAATTGTTAATATATATGCACCTAATAGATCCCTAAAATACATG
AAGCAAAACTGAGAGAATGCAAGGGAGAAAGAGATAATTCAACAATCATAGTTGGAGACCACTTTCTAAT
GCCTCATTTCAATATTATTGGACAGAAGAACTAGGCTGAATATCAAAAGGAAACAGAAACTTAACAACAC
TATGAACCAAGCAGGTCTAACAGACATCTATAGAAGAATCTGCCTTAACGATAGCAGAGAGTAGTCATTC
TTCCTGCCACACATGGAAACATTCTCCAGGCTAGACCATATGTTAGGCCATATAAACAAGTCTAAATAAA
TTTAAAATTATTTAGATCATACTTAAAATGTTATCTGTCCACAGTTGGGATAAATTAGAAGTCAGTAATG
CAAGAAATTTCGTTAAATTCACAAATAATATGTGGAAATTAAATCGCACACTCTTGAATATCCAAAGGAT
CAAAGAAGAAATCACAAGAGAAATTACAAAATCCTTTGTATAAATGAAAGAAAAACAAACACAACATACC
AAACTTATGGAATGCAGCTAATGGTGGGCTTACAGCAAAAATTTATAGCTGTGTATAAGTATATATTAAA

AACATATACCTTATTAAAAACAAAAAAGATCTCAAATCAATAATTTAAATATCCACCTTAAGTACTAAAA
GAACAAACCTCAAATAAGCAGAAAGAACAATAAAGATTTGAGTGCAAACAACAAAACGAAAAAAAAAAAA
AAAAAGAAGATAGAGCGACAGGCAAGAGCAATACGTAACTGAACGGAAGCACTACCG

>@6905266a-fff4-427d-9b25-55ff8bcfa7aa runid=1a9a02
AAAGATAGATATTTATTTTCACTGTCTTTCTTTCAGCAAAGAACATTTATTTTTTTAACATCTGAACAAG
AAATTTGTAACAGGCGAACAAGCACATTGAGCTTCCAGTGATCATAACATAATTAAACAAAGAGCGAAGT
GAGGATCCCGTGAACCCATGGCCTGACCCAGAATATTTATTTTTGCATTTATGCTCTGTTTTTTAATATA
GTTTCATTTGAGGTTGTTTTTTATAGCATGTTTTTTTATCATATGACTTAATCTTCGAGAGTTATTTGTT
GAAGAAGATAAACATATTTTTTCATTATGTGGCCCAGAAATACAGTGCTTTGTTGGGCCAACGAAAGTGT
TAACATCGCATCACCTTGACAAAGCTGAGGATACGCAACGGCAATTATCTTAGATGATATCCTTGTTTGC
TTATTTAGGCTCATTTTTATTGCATGAAGCATCATGGTCAAGGCTTAATTTCACTAAATGTCAAACCAAA
CTTTTCTCAAACCAGGGACCTGTCATCAAGATTAATTACAGTAATTATTTTGCGTATAATAACTGCTCAA
AGGTTATGAATCTGGCGAATGTTAACCTTCCGTTTTATTACAAGCAAGTACTATTATTTCTGATTTTATA
ATACAGTACCTATGTTTAATCAACTGAGGCCTCTCAACCAAATAACATCTCAGAGATTAAGTTATATATT
AAAAGCTTATGTAACATAAAAGCAAGTACATATAGTAGTGACTATATTTAAAAAAACAGAGCATAAAATG
CTTAAAAATGTAATATTTTTACTAAAATCAGATTATGGGATAATGTTGCAGGATTATACTTTATTGCATC
TTTTGTTTAATTGTATTTAAGACAGTAATCACTTGGGAAAAATATTAAATTATTAACATTGAGGTACACA
TTTTAGTCTCTTTATTTTTAAATTTCTTTGTTCCAGAGATTGTTTAAAATAAATATTGACAAAAAAAAAA
AAAAAACTAAAGAAAACGAAAAAAAAAAAAAAAAAAGAAGACAGATGACAGGCAAGAGCACATGTAACTGA
ACGAAGTATTACC

>@928c9746-c452-4bfc-ae13-ba002e4f89c5 runid=1a9a02
CAAATTCTGTCTAATTTAGCCTGCTATATGTCATGGTTTTAACTTGCAGATGCGCACAATTTTGCAATGT
TTTACAGAAAGTAATTGAGTTGAGCAAGCAATTCCTTATTTAAAAAAGTACGTATTATTTAGGATAATCA
TACTTCTCTGTAGATGCGCCATGCACCAAAAACTCTTAAATATTTAGCAATCAAATAGAAATGGAATGTG
GACTTACTAAATGGCTTTTTAATTCCTATAAGAGCATATTTTAGGTACCATCTGCCCTAATTATAGTTTT
AACATTTAAAACCAAAGTCCTACACTTGATTTATATTATATGTGGCTTTGCTGAGTCAAGGAAGTATCAT
GCACAAGGTTTAATTACTAAATGTCAAACCAAACTTTTTCTCAAACTGTAATTCATTATCTAAGATTACT
ATGGTACTATTTTGCAGGCTGCAACTGCTCAAAGGTTATGAATCTTATGAATGTTAACCTTTCCGTTTAC
GCGCGCACGGCTATTTCTGATTTTATAATAAGAAAATCTATGTTTAATCAACTGGAGGCCTTCTCAACCA
AATAAACATCTCAGAGATTGCTATATAATAAAAGCTTATGTAACATAAAAGCAAGTACATATAGTAGTGA
CGTTTAAAAAAACAGAGCATAAAATGCTTAAAAATGTAATATTTACTAAAACCAGATTATGGGATAATGT
TGCAGGATTATACTTATTGTATCTTTTGAGCGACCAGATTTGCACAGTAATCACTTGGGAAAATATTAAA
TTATTAACATTGAGGTACACAAAAAAAAAAAACAAATAAACAAAAAAAAAAAGAAGATAGAGCGACAGGC
AAGAGCAATATGCAACTGAACGAAGTACAATG

>@d51a9296-ec75-4381-b967-e82ac386edd0 runid=1a9a02
GATGTACTTCGTTCAGTTGCATTATTGCTTTTCTGTTGGTGCTGATATTGCTGGGCTTCA
TTCTGCAGTTGGTGCCCAGAACCACGGATCCTGAACTGGAAGAAAATGTCTATCCAGGTT
GAGCATCCTGCTGGTGGTTACAAGAAACTGTTTGAAACTGTGGAAGGAACTGTCACTCCT
GCTTTCCCACAGCTCATGCAACAGGCAGGATCCCCCTCTGGCTCACCGCGAGTCTCCTTC
AGTAATGGGCAGGACTCTTTGAAGTTGGATCTGAGCCATTTTACCACCTGTTTGATGGGC
AAGCCCCTCCTGCTAAGTTTGACTTTAAGAAAGGACATGTCACATACACGAAGGTTCGCC
GCACTGATGCTTACGTACGAGCAATGACTGAGAAAAGGATCGTCATAACAGAATTTGGCA
CCTGTGCTTTCCCAGATCCCTCACAAGAATATATTTTCGAGTTTTTTTCTTACTTTCGAG
GAGTAGAGGTTGCAGCAATGCCCTTGTTAATGTCTACCCAGTGGGAAGATTACTACGCTT
GCACAGAGACCAACTTTATTACAAGATTAATCCAGAGACCTTGGAGACAATTAAGCAGGT
TGATCTTTTTAGCTATGTCTCTGTCAATGGAGGCCTACTCACCCCACATTGAAAATGATG
GAACGTTTACAATATTGGTAATTACTTTGGAAAAATTTTCCAATTGCCTACAACATTGTA
AAAGATCCCACCACTGCAAACGAACAGGAAGATCAATAAGCAAGTCAGAGATCGTGTACA
ATTCCCCTGCAGTGACCGATTCAAACCATCTTACGTTCACTTAGTTTTGGTCTGACTCCC
AACTATATCGTTTTTGTGGGAACTACCAGTCAAAATTAACCTGTTCAAGTTCCTTTCTTC
ATGGAGTCTTTGGGAGTCTTCATGGATTGTTTTGGAATCAATGAAACCATGGGGTTTGGC
TTCATATTGCTGACAAAAGGAAGAATACCTCAATAATAAATACCAGAACTTCTCCTTTCA
ACCTCTTCCATCACATCAGCAACACCTATGAGACAATGGGTTTCTGATTGTGGATCTCTG
CTGCTGGAAGGGTAAGAAGGACACTGGACAAATGGGTTTACCTCCCATTGTTCCTGGAAA
TTACAGGGGTTTTACAGAGCTGCTGCACTCAGTCTGAATCTATCTAAAACAGCAAAGAGT
TGAGAGTCAAAATGTAATTGGAAAAACAGTGAGGAAGTTTAGGTAATCTGAACATCAAAA

CTATGATTAATGATTGAAAACTAATCAGGTCCACGAGCCCCCACCTGAAAAAAAAGATTC
TGTGACCACCGAGAGGGGTGAAGACTTGAGTTGACAAATCACTGGAAACTTGGGCCCATC
TTCTCTGCGAAAGGGAGAAAAGACATGCGTTTTTCAGACTAGCAAATCACTAAGAAGACA
ACAGCGCCTAAGGACACAAACAGAATGGTGACGGAAGACAGGGTAGAAAGTATCTCCAAG
GCTGCTGTTGTAAGCAAATCCATTAAACAGGTGCTTTATTTTAAGTCCTGTGTGTGGGCG
GGAGGAAATGGCTCTCTGATACACCTGGCTCAATAGCAGTTTCTGGGTTGTGGAATAAAG
AACAGGCAGGCACTTGTGCTTAAAGGCAGAATCATCTCTCTAAAATTATTTGTCATTGCC
TGTGCTCATGTTTGACTTTTTATTTTTTTGCAGATTTAAGGTTTGTTTATAATTACTTAT
ATTTGTAGTTTACGTGAGAACGCTAGGAAAGGTGAAAAAAATGCCAGAAAGGCTCCCCCA
ACCTGAGTTGAGAGATATGTACTTCCTTTGAATATTGACAAGGTAACCTGCTTCTCCTGT
GAATTTCAGATTTAGCAGAATGTTTCATCTCTCTCAGAATTGTCCTCTGCCTCATGTTTA
TATCATAAAGTCTTGAAATTTGAAGAGCTAGAAGGAGCTTTAAAAATAGAGCTAATTAAA
CTTCCTTCATTTTGCAAATGAAACTAAAGCTCAGAAGGAGTACTGTGACTTAGCAAAAGC
AACTGCAGCAGAGCCAGGATAAGAACCAGGTTTTTAAATTCTAAATCTGCCGTTCTTGTA
TTTACAAAATTGTCTTTAAAATATACTTCCTCGGTGAATAGTGCAGATCACTGATTCCTA
TGATACTTTATCAATAATATAAATAGTTTAAATAATTTAGAAGTTCTGGAGAGATGAAGC
ATCAAAAAGTACTCTTGTAGGAAACCAGTTCAGTATTTTGGTTTTCTATAAATCTTCTGA
AAAAAATATCACTAGAGGTTAAAAATCATTACTAACAATAGACAGTCATCTCTTTATTTC
TACTTTTCTTCCCTGTATTTAGAGACTGTCTTTTTAGAATAACCAGAGTAACATCTAAAA
TTTGTAGTGTGATGGAATACCAGTCAAAATCAATAAATATAATTCTCACACAAAAAAAAA
AAAAAAAAAGAATAGGCGACAGGCAGAAGTAGCAATACGTAACT

>@e28e7c7f-e095-4244-8bc5-e86f1e32e5b0 runid=1a9a02
CAAACAGCTGGTTCAGGTTACGTGTTGCTCTTGCTGTCGCTCTATCTTCTTTTTTTTTTT
TTTTTTTTGTTTGTTTTTTTTATGTGTGAAGATTATGTTTTATTAGTTTGACTGGTATTT
CCATCACACTACAAATTCTAGATGTTACTCTGGTTATTCTAAAAGACAGTCTCTAAATAC
AGGGGAAGAAAAAGTAGAAATAATAAAGAGATGACTGTCTATTGTTAGTAATGATTTTTA
ACCTCTGGTGATATTTTCAGAAGATTTATAGAAACCAAAATCAGCAATCAGCACCAACAG
AAAAACAATACTGTCGAGAAAAGC

>@5b069eaa-22b2-4acc-82d8-975edbcf37ff runid=1a9a02
GTTACGTATTGCTTGTTGGTTCGATATTGCTGGGCTTCATTCTGCAGTTGGTGCCAGAACTCTGATCCTG
GAATCAAAGAAAATGTCTATCCAGGCTGATATCCTGGGTTGCGTGGCTGGCAAGAAATCAGTTTGAAAAC
TGTGGAGGAACTGTCTCGCCGCTCACAGCCTGCGTAATAGTGCAGATCCCCTCCGCCTACCGGGCAGTCT
CCTTCGATGTGGGCCAGACTTCTGAAGTTGATCTGGAGCGATTTTACCACCTGTTTGATGCAAGTGCTTC
CTCCTGCACAAGTTTGACTTTTTGTAAAGAAGGACATGTCATATACTACAGAAGGTTCCATCTGCATTGA
TGCCATGTACGGGCAATGACTGAGAAAAGGATCGTCATAACAGAATTTGGCACCTGTGGGCTCAGATCCC
TGCAAGAATATATATTTTCCAGGTTCTTTTTCTTACTTTCGAGGAGTAGAGGTTACTGACAAATGCCCTT
TGTTAATGCTCCACCCAGTGGGGAAGATTTTCATTACGCTTGTACAGAGACCAACTTTTACATAAAGATG
TTAATCCAGACCTGGTGACAATTAAGCAGGCTGGATCTTGCAACTATGTCTCTGTCAATGTAGCTTCACT
GTTTTATCTTTACATTGAAAATGATGGAACTGTTTACAATATTGCAATTGCTTTGAAAATTTTCAATTGC
CTAAGCAACATTGTAAAGATCCCACCACTGCAAGCAGACAAGGGAAGATCCACAGCAGCCAGAGATCGTT
GTACCCCTGCAGTGACCGATCTTAAGCCATCTTACGTTCATAGCTCTGGTCTGACTCCCCAACTATAATA
TGTTTTGTGGAGACACCAGTCAAAATTAACCTGTTCAAGTCTTTCATGGAGCTTTTGGGTTTTCGAACCA
TATGGATTGTTTTGAGTCCCAATGAAACCATGGGTTTGGCTTCATATTGCTGACAAAAAGGAAAAAGTAC
TCTCAATAATAATACAGAACTTCCCTTTTCAATCCTCTTTCCATCACATCAAATACCTATGAAGACAATG
GGTTTCTGATTGTGGATCTCTGCTGCTGGGAAAGGATTTGAGTTTGCCGATAATTACGGTATTTAGCCAA
TTTATTTAGAACTGAAGAGAAAATGCTAGAAAGGCCCCCAACTTGGAAGTAGAGAGATATGTACTTTGAA
TATTGACAAGGCTGACACAGTAAAGAATTTAGCTACGCTCTCATTTACAACTGCCACTGCAATTTGTGCA
GTGATTTAGACTATCTGGCTGGCTTTAAAGCCCTCTTTCAGCCTTGCCTACATTTTGAGTTTCTAATCAA
TTAACAGAAAGCATTGGTGGGGAAACCTTGCACATATGCGTATGGATCTCATTTGAACTACTTTATTCCG
TAGATAGGTCAAGCTGAATGTCAAAACTAAAGAACTTTCAGTTTGGCAAGAGCCTGATTCATGTTCCACT
GTACGATCTTGTTTCTCACCCAGATGCTCTCTAAAGAAGATGATGGGTGTAGTCTCCTGAGGTGGTGGTG
AGCCCAGGAGCAGGACAAAAGCCCGCTTATCTCCTGATTCTGAATGCCAAGGATCATGAAGCTGCCTGGC
TGAAGGGAGATTTCAACATCCCTGTCACCTTCATGGATCGTTCAAAAATCTTGAGCATACTCCAGCAGCA
TATGTTTTGCAGCAAAACTGAGAAAATCAGCTTCAGGTCTGCAATCTAAATTCCCTGCCTACCTTAGCCT
GCATGTCATGGTTTTAATCTCTGTAGATGTACAATTTTGCAATGTTTTACAGAGCACTGAGTTGAGTGGG
CAATTCCTTTATTTAAAAAAAAGTATGTATTTAGATAATCATACTTTCCAGAGACAGGCCAACTGAAAAA
CTCTTTAAATATTTAGGCAATCAAATAGAAAGTAATGGGACTTGGCCTTTAAATGGCTTAATTCCTATTA

TAAGAGTATATTTTAGTACCTATCTGCTCCAATTATATTTTTAATATTTAAAACCAAAGTCCTCACTTGA
TTTATATTATATGGCTTTGCTGAGCCAAGGAAGTATCAAGTAATAAGGCTTAATTACTAAATGTCAAACC
AAACTTTTTCTCAAACTAGGGACTATCATCTAAGATTAATTACAGTAATTATTTTATGTATACGTAACTG
CTCAAAGGTTATGAATCTTATGAATGTTAACCTTTCCTGTTTATTACAAGTAAGTACCATTATTTGATTT
TATAATAAGAAAATCTATGTTTAATCAACTGAGGCTCTTCAACCAAATAATATCTCAGATTAAGTTATAT
ATTAAAAGCTTATGTAATATAAAAAGCAAGTATATAGTAGTACCATATTTAAAAAAAAAAAAAATAGAGC
ATAAAATGCTTAAATGTAATATTTACTAAAATCAGATTATGGATATCGTTGTAAGGATTATACTTTATTG
CATCTTTGTTTAATTGTATTTACATCAGTAATCACTGGGAAAAATATTAAATTATTAAATACTGAGGTAT
TAATATTTTAAGCCTTTTTGTTTTAAATTTCTTTTTGTTCCAGATAGATTGTTTAAAATAAATATTGACA
AAAAAAAAAAAAAAAAAGAAGATAGAGCGACAGGCAAGTAGTAATACGCAACTGAAACGAAGTGATAAAT
G

>@f7b1f02f-9ce3-41c4-8703-bf3e16ba7b30 runid=1a9a02
GTTACGTATTGCTACTCTGCCTGTCGCTCATCTTCTTTTTTTTTTTTTTTTTTTGACAATATTTATTTTT
AAACAATCTCTGGAACAAAAGAAATTTAAAAAAACAAAAGGCTTAAAATGTATTAATACTCTCAATGTTA
ATAATTTAATATTTTTCTCCCAAGTGATTGCACAACGCTAAATACAATTAAACAAAAAGATGCAATAAAG
TATAATCCTGCAACATTATCCCATAACTGAAAACCTTAAATATTTAGCAATCAACAGGGAAAATGAATGG
ACTTACTAAATGGCTTTTAATTCCTATTATAAGAGCATATTTTAGGTACCTATCTGCTCCAATTATATTT
TAACATTTAAAAACCAAAGTCCTCTACACTTGATTTATATTATATGTGGCTTTGCTGAGTCAAGGGAAGC
TATCATGCAATAAGGCTTAATTACTAAATGTCAAACCAAACTTTTTTCTCAAACCAGGGACTATCATCTA
AGATTAATTACAGTAATTATTTTGCGTATACGTAAACTGCTCAAAGGTTATGAATCTTATGAATGTTAAC
CTCTTCCTGTTTATTACAAGTACTATTATTTCTGATTTTATAATAAGAAAATCTATGTTTAATCAACTGG
AGGCCTTCAACCAAATAACATCTCAGAGATTAAGTTATATATTAAAGCTTATGTAACATAAAAAGTAAGT
ACATATAGTAGTGACCATATTTAAAAACAGAGCATAAATGCTTAAAAATGGGGTCATTTACTAAAATCAG
ATTATGGGATAATGTTGCAGGATTATACTTTATTGCATCTTTTGTTTAATTGTATTTAAGCACAGCAATA
CGTAACTGAACGAAGTATTACCG

>@a3622fdb-3848-4cc7-b896-789e83e31809 runid=1a9a02
CCAGCGGTTGCATTGTTGAATGTTTGTTTTCTTTTTTTTGAAAGCTTCTTCTTTTCGCCATTTATCTCAT
CGCCATTACCTCTATTATTGACATAAACAAATAAAAAGAGCATGGGTGATAACCCGTAACAGGTTCATGG
TCTGATTTTGCAATATTTAAGATTTGCGCGCCTGTTTTTTAGTCAATAATTATAGTATGTTACATAAGCT
TTTGGTATTCATCTTCGAGAGCGTTATTTGTTGAGAGGTTTCAGCTGATTAAACAGCGATTTGGCTATAA
TCAGAAATAATGAGGAAGCAGCCAAACGCGTGAAAGGTCAACACTGCAAGATTCATACCTTTGAAAGGTG
CCAGGCGTCGGCTCATCGTAATTAATCTTAGATGAGGTCTTTGTTTGATAAAGCTTGTTTGACAGTACTG
TTTTTTACTGCATGATAGCTTCGATTGCAAAAGCCAATATTATAAACAGTGTAGAGACTTTGCTTCACCA
AAATATAATGATAGACAGTACCAAAACATGCCTTTTTAATTCCCACTATAAGAGCACATTTTAGGTACCC
ATCTCCGCTCCACCATATTTTTAACATTTAAAACTCAAAGTCCCCACACTTCTGATTTATATTATATGGC
TTTGCTGAGTCAAGGAAGCATCGCAAAAGTAATAAGGCTTAATTACTAAAATGTCAAAACCAAACTTTTT
CCTCAAACTAGGACTATCATCTAAGATTAATTACAGTAATTATTTTGCGTATACGTAACTGCTCAAAGGT
TATGAATCTTAAGTGAATGTTGACCTTCTGCTTTATTGCCAAGCAAGTACTATTATTCTGATTTTATAAT
GGCACCCATGTTTAATCAACTGAGGCCTCTCAATCAAATAACATCTCAGAGATTAAGTTATATTAAAGTT
ATGCAAATATTAAAAGGCACATAGTAGTGACTCATATTTAAAAAACAGAGCATTAAAAATGCTTAAAAAT
GTAATATTTAATAAAATCAGATTATGGGATAATGTTGCAGGATTGAGTTATTTTATTAAGATCTTTTTTG
TTTAATTTGGGATTTAAGCATTGTGCAAATCACTTGGAAAATATTAAATTATTAACATTGAGGTAAAAAA
AAAAAAAAAAAAAAAAAGGAAAATAAGAAGATAGAGTGACAGGCGGCAGTACGTAACCGAATTAGCATAC
CG

>@e963c893-6168-4551-af0b-c5d77ce789eb runid=1a9a02
GCTACGTACGTTTTCGTTGCGCTGATATTGCTGGGTCTATTCTGCAGTTGGTGCCAGAACTCTGATCCCG
GAACTGGAAGAAAATGTTCATCCAGGTTGAGCATCCTGCTGGTGTTACAAGAAACCGTTTGAAACTGTGG
AGGAACTGTCCTCGCCGGCTCACAGCTCATGTAACAGGCAGGGATCCCTCTCTGGCTCACCGGCAGTCTC
CTTCGATGTGGGCCAGGACTCTTTGAAGTTGATCTGAGCCATTTTACCACCTGTTTGATGGGCAAGCCCT
CCTGCACAAGTTTGACTTTTAAAGAAGGACATGCTCAAGCATACCACAGAAGGTTCATCCGCACTGATGC
TTACGTACGGGCAATGACTGAGAAAAGGATCGTCATAACAGAATTTGGCACTCCAGAAGCTTCCCAGATC
CCTGCAAGAATATATTTTCCAGGTTTTTTTTCTTACTTCGAGGAGTAGAGGTTACTGACAATGCCCTTTT
GTTAATGTCTACCCAGTGGGGAAGATTAATACGCTTGCACAGAGACCAACTTATTACAAAAGATTAATCC
AGAGACCTTGAGACAATTACAGGTTGATCTTTGCAACTATGTTCTGTCAATGGGGCCACTGCTCACCCCC
TTATTGAAAATGATGGAACCGTTTACAATATTGGTAATTGCTTGAAAAAATTTTTCAATTGCCTACAACA
TTGTAAAGATCCCACCACTGCAAGTAGACAAGGAAGATCCAATAAGCAAGTCAGAGATCGTTGTACACCC

CTGCAGTGACCGCTGATTCAGCTTTCATCTTACGTTCATAGTTTTGGTCTGACTCCCAACTATATTTGTT
TTGTGGGAGACACCAGTCAAAATTAACCTGCTCAAGTTCCTTCATGAGCTTTGGGGAGCCAACTACATGG
ATTGTTTTGAGTCCAATGAAACCAAGGGGTTTGGCCATATTGCTGACAAAAAAAGGAAAAAGTACTCTAA
TAATAAATAACTTCTCCTTTCAACCTCTTCCATCACATCAACACCTATGAAGACAATGGGTTTCTGACGG
TGGATTCCCTGCTGCTGGAAGGATTTGAGTTTGTTTATAATTATTTATATTTAGCCAATTTACGTGAGAA
CTGGGAAGAGGTGAAAAAATGCCAGAAAGGCTCTGCAACCAGTTAGAGATATGTACTTCCTTTGAATATT
GACAAGGCATGGGACAGGCAAGAATTTAGTCACGCTCTAATACAACTGCCACTGCAATTCTGTGCAGTGA
CGAGACTATCTGGCTGGAGCCTGAAGTTTCTCTTTTCAGGGTCTTCACACAAGCATTTGAGTTTCTCAAA
TCAATTACCAGAAGTATTGTGGAAACCTTATGACATATGTATGGATTCATTTTCTGAATCACTATTTGTT
CCAGATAGGCTCTGTAAGCTGAATCGTCAAAACTAAAGAAACTTGGGTTTGGCAAGAGCCTGATTCATAC
CCACTGTGAACCCATCTTTGTCTCTCACCCAGATGCCTTGGAAGAAGATGCTGATGGTGTTCTGAGTGTG
GTGGTGAGTCTGAGAGCAGGACACAAAAGCTGCTTTATCTCCTGGTGATTCTGTCGCTAAGGACTTATGA
AGTTGCCTGGCTGAAGTGAGATTAAATATCCCTGTCACCTTCATGGACTGTCTAAAAAATCTTGAGCATA
CCTAGCAAGATAAGTGTTTTTGGTAGCAAACTGGAGAAATAACTAGTCAGGTCTGCAATCAAATTCTGTC
TAATTTTAGCCTGCTATATGTCATGGTTTTAACTTGCAGATGCGCACAATTTTGGCAATGTTTTACAGAA
AGTACTGAGTTGAGCAAGCAATTCCTATTTAAAAAAAAAAAGTACGTATTTGATAATCATACTTCTCTGT
GAGACAGGCCAGTAACTGAAAACTTTAAATATTTAGCAATCAAATAGGAAATGAATGTGACTTACTAAAT
GGCTTTTAATTCTATTATAGCATATTTTAGGTACCTATCTGTCTAATTATATTTTTAACATTTAAAACCA
AAAGTCCTCTACACTTGATTTATATTATATGTGGCTTTGCTGAGTCAAGGAAGTATCATGCAATAAGGCT
TAATTACTAAATGTCAAACCAAACTTTTCTCTTTCAAACTAGGGACTGGTCATCTAAGATTAATTACAGT
ACTATTTTATGTATACGTAACTGCTCAAAGGTTATGAATCTTTATGAATGTTAACCTTTCCGTTTATTAC
AAGTACTATTATTTCTGATTTTATAATAAGAAAATCTGAGTATTAATCAACTGAGGCCTCTCAACCAAAT
AATATCTCAGAGATTGCTATATATTAAAAGCTTATGTAACATAAAAGTAAGTACATATAGTAGTGACTAT
ATTTAAAAAAACAGAGCATAAAATGCTTAAAAATGTAATATTTACTTTAAAATCAGATTATGGGATAATG
TTACAGGATTATACTTTACTGCATCTTTTTTTTGTTTAATTGTATTTAAGCATCTGTGCAATCACTTGGG
AAAAAATATTAAATTATTAACATTGAGGTATTATCATATTTTAAGCCTTTGTTTTAAATTTCTTTGTTCC
AGAGATTGTTTAAAAATAATATTAAAAAAAAAAAAAAAAAAAGAAGATAGGAGCGACAGGCAAGAGCAATA
CGTAACTGAACGAAGCACTACCGATTTTACGC

>@43b6d550-0bdb-4335-b8dc-0318acca5000 runid=1a9a02
ATCATGCAATAAGGCTTAATTACCAAATGTCAAACCAAACTTTTCAAACCAGGACTATCATCTAAGATTA
ATTACAGTAATTATTTTGCGTATACGTAACTGCTCAAAGGTAATCTTATGAATGTTAACCTTTCCGTTTA
TTACAAGCAAGTACTATTATTTGATTTTATAATAAGAAAATCTATGTTTAATCAACTGAGGCCTCTCAAC
CAAATAACATCTCAGAGATTAAGTTATATATTAAAAGCTTATGTAACATAAAAGCAAGTACACATAGTGT
GTGACTATATTTTTTAAAAAAACAGAGCATAAAATGCTTAAAATGTAATATTTACTAAAATCAGATTTAT
GGGATAATGTTGCAGGATTATACTTTATTGCATCTTTTTTTGTTTAATTGTATTTAAGCATTGTGCAATC
ACTTGGGAAAAATATTAAATTATTCAATACTGGAGGTATTAATGTTATTTTAAGCCTTTTGTTTTAAATT
TCTTTTGTTCCAGAGATTGTTTAAAACAACATTGACAAAAATAAAAAAAAAAAAAAAAAAAAAAAACAAA
CAAAAAAAAAAAAAAAAAAAAAAGAAGATAGAGTTGACACAGGCAAGTAGCAATACGTAACTGAACGAAG
CACAAC

>@c9bd1a7e-d69c-4c61-8bc6-99fba7a6267f runid=1a9a02
TATTTTGCTTCGTTCGGTGCGTATTGCTTTCTGTTGGTGCTGATATTGCTGGGGGCTTCA
TTCTGCAGTTGGTGCCAGAACTCTGGATCCTGAACTGGAAGAAAATGTCTATCAGGTTGA
GCATCCTGCTGGTGATTGAAACTGTTTTGAAACTGTGGAACTGTCTCGCCGCTCACGCTC
ATGTAACAGGCGGATCCCCTCTGGCTCTGGCAGTCCCACTCCTTCGATGTGGGCCAGGAC
TCTTGAAGTTGGATCTAGGCCATTTTACCACATTTGATGGGCAGCCTCCTGCACAGAAGA
GTTTGACTTTAAAGAAGGACATGTCTTTACAGAAGGTTCATCCGCACTGATGCTTGTACA
GGCAATGACCAGGAAAAAGGATCGTCATAACAGAATTTGCTACCTGTGCTGAGATCCCTA
CAAGGAGAATATATTTTCAGGATTTTCTTACTTTCGAGGAGTAGAGGTTACTGACAATGC
CCTTGTTAATGTCTACCCAGTGGGAAGATTACTACCTTGCTAGAGACCAACTTTATTACA
AAGATTAATCCAGAGACCTTGGAGACAATTAAGCAGGTTGATCTTTGCAGCTATGTCTCT
GTCAATGGGGCCTGCTCACCCCACATTGAAAATGATGGAACCGTTTACAATATTGGTAAT
TGCTTTGGAAAAAATTTTCAATTGCCTGGCATTGTAAAGATCCCACCACTGCAAGCGAAC
AGGAAGATCAATAAGCAAGTCAGAGATCGTTGTACAATTCCCCTGCGGTGACCGATTCAA
GAAGCCATCTTACGTTCATAGAGTTTTGGTCCCTGACTCTCCCAACTATATCGTTTTGTG
GGGAACACCAGTCAAAATTAACCTGTTCAAGTTCCTTTCTTCATGGCCAAAGTCTTTGGG
AGCCAACTACATGGATTGTTTTGAGTCAATGAAACCATGGGGTTTGGCTTCCATATTGCT
GACAAAAAAGGAAAAAGTACCTCAATAATAAATACCAAGGCTTCTCCTTTCGACCTCTTC

CATCACATCACTTGCACCTAGAAGACAATGGGTTTCTGATTGTGGATCTCTGCTGCTGGA
AAGGATTTGAGTTTGTTTATAATTACTTATATTTATGGTACGTGAGAACTGGGAAGAGGT
GAAAAAAAAATGCCAGAAAGGCTCCCCCAACCCTGAAGTTAGGAGATATGTGCTTCCTTTG
AATATTTGACAAGGCTGACACAGGCAAGAATTTAGTCACGCTCCCAATACAACTGCCACT
ACTGCAATTCTGTGCAGTGAGACTATCTGGCTGGAGCCCTGAAGTTCTCTTTTCAGAGAC
TATAAATCAAGCATTTGGAGTTTCCTCAAATCAATTACCAGAAGTATTGTGAGAAACCTT
ACACATATGCGTATGGACAGCTTGAATCACTTTGTTCCAGATAGGCTCTGTAGCTGAATG
TCAAAACTAAAGAAACTTGGGTTTGGCAGAGCCTGATTCATCACCCATCAGAACCCCATC
TTGTTTCTCGCAGATGCCTTTAGAGAAGATGATGGTGTAGTTCTGAGTGTGAGTGGTGAA
GCCCAGGAGCAGGACAAGCCTGCTTATCTCCTGATTCTGAATGCCAGGACTTAAGTATTG
CCCAGGCTGAAGTGGAGATTAACATCCCTGTCACCTTTCATGGACTGTTCAAAAAATCTT
GAGCATACTCCAGCAAGATATGTTTTTAGTAGCAAAACCTTTGAAAATCAGCTTCAGGTC
TGCAATCAAATTCTGTTCAATTTTAGCCTGCTATGTCATGGTTTTAACTTGCAGATGCAC
AATTTGCAATGTTTTACAGAAGCACTGAGTTGAGCAAGCTTAATTCCTTTATTTAAAGTA
CGTATTTAGATAATCATACTTCCTCTGTGAGACAGGCCATAACTGAAAAACTCTTAAATA
TTTAGCAATCAAATAGGAAATGAATGTGGACTTACTAAATGGCTGAAAATTCCTATTATA
AGAGCATATTTTAGGTACCTATCTGCTCAATTATATTTTAACATTTAAAAAACAAAGTCC
TCTACACTTATTTATATTATATGTAGCTTTGCTGAGTCAAGGAAGTATCATGCAATAAGG
CTTAATTACTAAATGTCAAACCAAACTTTTTCTCAAACCAGGGACTATCATCTAAGATTA
ATTACAGTAATTATTTTGTATATACGTAACTATAAAGATTATGAATCTTATGAATGTTAA
CAACATACGATTTATTACAAGCAGTACTATTATTTCTGATTTTATAGCAGAAAATCTGTG
TTTAATCAACTGAAGGCCTCTCAACCAAATAACATCTCAGAGATTAGATTATATATTAAA
AACATGTAACATAAAAGCAAGTACATATAGTAGTGACTATATTTAAAAAAGCAACATAAA
ATGCTTAAAAATGTAATATTTACTAAAATCAGATTATGGGATAATGTTGCAGGATTATAC
TTTGTGCATCTTTTGTTTAATTGTATTTAAGCATTGCAATCACTTGGGAAAAATATTAAA
TTATTAACATTGAGGTATTAATACATTTAAACCTTTTGTTTTAATTTCTTTTCTTCAGAG
ATTGTTTAAATAAATATTGACAAAAATAAAAAAAGAAAAAAAAAAAGAAGATAGAGCGAC
AGGCAAGTAGCAATACGTAAC

>@29755ad7-6e08-4bc5-ae6f-71fdb0325219 runid=1a9a02
CTTTCCGTTTATTATGGAAGCAAGTACTACGCTTCTGATTTGGCAACAAGAAAATCTATGTTTAATCAAC
TGAGGCCTCTAAACCAAGCCACTTATCTCAGAGATTGCTATATATTAAAAGTCATGTAACATAAAGGCAG
TAGTGACTATATTTAAAAATAGAGCATAAAATGCTTAAAATGTAATATTTACTAAAATCAGATTATAGGA
TGTTGTTGCAGGATTATACCTATTGTGATCTTTTTTATTTAATTAGATTTACATCAGCAATCACTTGGGA
AAATATTAAATTCATACATTGAGGTATTAATACATTTTAAGCCTTTTGTTTTTAAATTTCTTTGTTCCAG
AGATTGTTTAAAACAAACATTGACAAAAAAAAAAAAAAAAAACAAAAAAAAAAAAAAAGAAGATAGAGCGA
CAGTGCAGCAATACGTAACTGAACGAAGCACAAC

>@bb840fee-400c-4858-9439-fb612ed78dfe runid=1a9a02
TACCTCGGTACTTCGTTCAGTTACATGTTGCTGGCAGAGATTGGGGCAGGAGGAAATAGC
TCTGATACACACCTAGCTCAATAACGGTTTTCTGGGTTGTGGAATAAGAACAGGCAGGCA
CCTTGTGCAAAGAGCAAGAATCATCTCTCTAAAATTATTTGTCGTGCCTGTGCTCATGTT
TGACTTTATTTTGCAGATTGAGGTTTGTTTATAATTATATATTTAGCGATTACGTGAGAA
CTGGGAAGAGGTGAAAAAAAAAAAAAATACCAGAAAAGGCTCCCAACCTGAGTTGGAAGAT
ATGTACTTCCTTTGAATATTGACAAGGTAACCTGCTTCTCTGTAGATTTCAGATTTAACC
AGAATGTTTTCATCTCTCTCAAGAATTGTCCTCCTGCCTCATGTTTATCATAAAGTCTTG
AAATTTGAGCTAGAGCTTTAAAAATAGACTAGTAAACTTCCTTCATTTACAAAATGAAAC
AAAAGCTCAGAAAAATTACTGTGACTTAACCAACAACTACCCAGCAAGAGCCAGAACCAG
GTTTTAAATTCCTAAATCTGCCGTTCTTGTATTTCAGAGTGTCTTTAAAATATACTTCCT
CGGTGAATAGTGCAGATCCACTGATTCCTATGATACTTTATCAATAGCCATAAATGGTTT
AAATAATTTGAAGTTCTACTGGAAGATGAAACATCAAAAAGTACTCTTGTAGAAACAGTT
CATTATTGGTTTTCTATCGTCTTCACAGAAAAATATCACTAGGGTTAAAAATCATTACTA
ACAATAGACAGTCATCTCTTTTTATTTCACTTTTCTTCCCTGTATTTAGAAGACTGTCTT
TAGAATAACAAATGTCTTATCGAATTTGTAGTGTGATGCGATACCAGTCAAAATCAATAA
AATATAATTCTCTGACTCAGAGATGAGAGCGACAGACAAATAACAATACAGTAAC

>@33cd27e9-8975-4acd-adce-f033b9f9324b runid=1a9a02
GGTTGAGCATCCTGCCAGGTTACAAAGAAACTGTTTGAAACTGTGGAGGAACTGTCCGGCTCACAGCCCA
TGGCCAGGCAGGATCCCCTGGCTCTGCTCGGCAGTCTCCTTCGATGGGCCAGGACCTTGGAAGTTGGATC

TGCTTATTTACCACTCCGCTGTGGGCAAGCCCTCCCTGCACAAGTTTGATTTTAAAGAAGACATGTCACA
TACCACAGAATAAGGTTTTTCTTACTGAGGAGTAGAGGTTACTGACAATGCCCTGTTAATGTCTACCCAG
TGGGGAAGATTACTACGCTTGCACAGAGACCAACTTTATTACAAAGATTAATCCAGTGGTCTGAGACAAT
TAAGCAGGTTGATCTGCAACTATGTCTCTGTCACTAGGTCACTGCTCACCCTCACATTGAAAATGAGATG
GAACTGTTTACAATATTGGTAATGCTTTGGAAAAATTTTCAATTGCCTACAACACAAAGATCCCACCACT
GCAAGCAGACAGGAAGATCCAATAAGCAAGTCAGAGATCGTTGTACAACCCTGCAGTGACCAGATTCAAT
ATCCCACGTCTATATTTTGGTCTGATCCCCAACTGTCGTTTTTGTGGAGACACCAGTCAAAAATTAACCT
GTTCAAGTTCCTTTCTTCATGAGTCTTGGGGAGCCAACCACATGGATTGTTTTGGAGTCCAATGAAACCA
TGGGGTTTGGCTTCATATTGACAAAAAGGAAAGTACCTCAATAATAAGCAGAACTTCTCCTTTAACTCTC
TTCCATCACATCAACACTTATGAAGACAATGGGTTTCTGGATTGTGGATCTCTGCTGCTGGAAAGCTGGG
TAAGAGAAAGGACACCAACAAATGGGACACTCCCATTGTTCCTGGAAATTACGGTTTTTACAGAGCTGCT
GCACTCAATCTGAATCACACTAGACAAAAAGAAGAGTGGTGAGGTCAAAACAATTGAAAGCAGTAGGAAG
TTTGTGTAATCTGAACATCAAAACTATGATTAATGATTGAAAACTAATCGGGTCCACGAACCCCACCTGA
AACAAAAGATTCCTGTGACCTGAGAGGTGAAACTTAAAGCTGACAAAATCCCTTGGGGAAACTTGAGCCC
ATCTTCTGCGAAGGAGAAAAGACGTTTTTCTGTGACTAGCCAAATCAACAAGAAGACAACAGCACCTAAG
GACACAAACAGAATGGTGACAGGAAGACAGAATAGAAAGTATCTCAAGCATGCTAAGCATTAAACAAATC
CATTAAGCAGGTTAAGCTTTATTTTCATCCTGTGTGGGCGGGAGGAAATGGCCTTGATACACCTGGGCCC
AATAGCAGTTTCTGGGCTGTGGAATAAAGAACAGGCAGGCACTGTGCTTAAAAAGGCAAGAATCATCTCT
AAAATTACTTGTCATTGCCTGTGCCTATGTTTGACTTTATTTTGCAGACGGAGTTTGTTTATAATTACTT
ATATTTAGCCAATTTACGTGAGAACTGGGAAGAGGTGAAAAATGCTAGAAAGGCTCCCAACCTGAAGTTA
GGAGATATGTACTTCCTTTGAATATTGATTAAGGTAATGCTTCGTAGATTTCAGATTTAAATCCAGAATG
TCTCTCCCATCTCTCTGTAGTCTGTCCTCCTGCCTCATGTTTATACATGTAAAGTCTTGAAATTTGAGAG
CTAGGGAAGGAGCTTTAAAAATAATATTAATTAAACTTCCTTCATTTTACAAATGAAACTAAAGTTCAGA
AGATATATAATTGTGACTTAGCAGCAAACGTAAGGCAGCATATTGCGGATAAAACCTGTACTTTAAATTC
CTAAATCTGCCGTTCTTGTATTTACAAATTGTCTTTAAAATATACTTCCTCGGTGAATAGTGCAGATCCA
CTGATTCCATGATACTTTATCAATAATATAAATAGTTTAAATAATTTGAAGTTTCTGAGATGAAGCATCA
AAAAGTACTCTTGTAGGGAAACCAGTTCTATTTTAGTTTTCTATAAATCTTCTGAAAAATATCACTAGAG
GTTAAAAATCACAACAATAGACAGTCATCTCTTTATTTCACTTTTTCTTCCCTGTATTTAGAGACTGTCT
TTAGAATAAACTAGAGTAACATCTAGAATTTGTAGTGTGATGGAAATACCAGCCAAAACCACAAATATAA
TTCTGCAAAAAAAAGAAGATAGATGACAGGCGCTACCACGTAACTGAACGAAGCACACC

>@6e98f670-c619-41cd-90ea-62b4fca5a437 runid=1a9a02
GCTGAAGTGGAGACAACATCCCTGTCACCTTTCATGGATTGTTCAAAAATCTTGAGCATACCCAGCAAGA
TATGTTTCTTTGCAGCAAACTGAAAATCAGCTTCAGGTCTGCAATCAAATTCTGTTCAATTCTAGCCTGT
TATATGTCATATATTTTAATCTCTTGCAGATGCGCACAATTTTGCAATGTTTTACAGATAAAGCAATTGA
GTTGAGTAAGCAATTCCTTTATTTAAAAAAAAGTATGTATTTAGATAATCATACTTCTCTGGGGAGATAT
GCCATAACTGAAAACTCTTAAATATTTAGCAATCAAATAGGAAATGAATGTGGACTTACTAAATGGCTTT
TTAATTCCTAATATAAGAGAGCATATTTTAGGTAACATTTTGCTCCACCTATATTTTTAACATTTAAACC
AAAGTCCTTTCTACACCTGATTTATATTATATGTGGCTTTGCTGAGTCAAGGAAAACATGCAATAAGGCT
TTAATTACTAAATGTCAAACCAAACTTTTTTCTCAAACCAGGGAATTATCATCTAAGATTAATTAGTAAT
TATTTTATGTGGCATAACTGCTCAAAGATTATGAATCTTATGAATGTTAACCTTCTGTTTATTACAAGGT
ACTATTATTTCTGATTTTATACAAGAAAATCTGTGTTTAATCAATTGAGGCTCTCTCTCAACCAAATAAC
ATCTCAGATTAAGTTATATATTAAAAGCTTATGTAACATAAAGCAAGTACATATAGTAGTGACTATATTA
AAAACAGCATAAAATGCTTAAAATGTAATATTTACTAAAATCAGATTATGGGATAATGTTGCAGGATTAT
ACTTTATTGCATCTTTGCTTAATTGCATTTAAGCATCAGTAATCACTTGGGAAAATATTAAATTATTAAC
ATTGAGGTATTAATATTTTAAGCCTTTGTTTTTAAATTTTTCCAGAGATTGTTTAAAATAACATGACAAA
AATAATAAAAAAAAAAAAAAGAAGATAGGAGTTGACAAAAGAGGAGCAATAATGCAACTGAACGAAGTACA
TC

>@294f75b3-df06-45a0-bcd9-0de09d900751 runid=1a9a02
CGTTGTACTTCGTTCAGTTACGTATTGCTTTTCTGTTGGTGCTGATATTGCTGTTCATTC
TGCAGTTGGTGCCAGAACTCTGGATCCTGAACTGGGAAGAAAATGTCTATCCAGGTTGAG
CATCCTGCTGGTGGTTACAAGAAACTGTTTGAAACTGTGGAGGAACTGTCCTCGCCGCTC
ACAGCTCATGTAACAGGCAGGATCCCCCTCTGTAGCTCACCGGCAGTCTCCTTCGATGTG
TAGACAGGACTCTTTGAAGTTGGATCTGAGCCATTTACCACCTGTTTGATGGGCAAGCCC
TCCTGCACACAAAGTTTGACTTTAAAGAAGGACATGTCACATGCCACAGAGGTTCATCCA
CCTGATGCTTACGTACGGGCAATGACTGAGAAAAGGATCGTCATAACAGAATTTGGCACC
TGTGCTTTCCCAGATCTACAAGAATATATTTTCCAGGTTTTCTTGCTTTCCGAGGAGTGA
GGTTGCACAGCCAATGCCCTTGGTAATGTCTACCAGTAGGGGAAGATTACTACCTTGCAC

AGAGACCAACTTTATTACAAAGGTGTCAAGTTTTGGAGACAATTAAAGCAGGTTGATCTT
TTGCAACTATAATCTCTCAATGGGGCCTACTCACCCACATTGAAAATGATGGAACGTTTA
CAATATTGGTAATTGCTGGAAAAAATTTTTCAATTGCCTACAACATTGTAAGATCCCACC
ACGCTACAGAGACTTGAGACAAGGAAGATCAATAAGCAAGTCAGAGATCGTTGTACAATT
CCCCTCGTGACCGATTCAAGCCATCTTACGTTCCTGGTTTTGGTCTGACTCCCAGCTATA
TCGTTTTGTGGAACACCAGTCAAAATTAACCTCATTCAAGTTCCTTTCTTCATGAGTCTT
TGAGAGCCAACTACATGGATTGTTTTGAGTCAATGAAACCATGGGGTTTGGCTTCATATT
ACTTTGACAAAAAAAAGGAAAAAATACCTCCAATAATAAATACAGAACTTCTCCTTTCAG
CCTCTTCATCCACATCAGCACCTATAGAGACAATGGGTTTCTGATTGTGGATCTCTGCTG
CTGGAAAGGATTTGAGGTTTTGTTATAATTACTTATATTTAGCCAATTTACGTGAGAACT
GGGAAGGAGTGAAAAAATGCCAGAAAGGCTCCCCAACCTGAAGTTGGAATATGCTTCCTT
TGAATATTGACAAGGCTGACACAGGCAAGAATTTAGTCACGCTCCCAATACAACTGCCAC
TGCAATTCTGTGCAGTGACGAGACTATCTGGCTGGAGCCTGAGTTCTCTTTTCAGGGCCT
CGTCAAGCAACATTTTGAGTTTCCTCAAATCAATTACCAAGTGTTGTAGGAAACCTTACA
TATGCGCGTATGGACTTGGCAGATCACTTTGTTCCAGATAGGCTCTGTAAGCTGAATGTC
AAAACTAAAGAAACTTGGGTTTGTAGAGGCCTGATTCATACCCATCAGAACCATCTTTGT
TTCTCACGAGTACCTTGGAAGAAGATGATGGTGTAGTTCTGAGTGTGGTGGTGACCAGGA
GCAGACAAAGCCTTATCTCCTGATTCTGAATGCCAGGACTTAAGTGAGATTGCTTGGGCT
GAAGTGGAGATTAACATCCCTCAACCTGCTGCTGGATATTTGAAAAATCTTGCTGGGCAT
ACTAACAGATAATGTTTTTAGTAGCAAAACTGAAAGGAAATCAGCTTCAGGTCTGCAATC
AAATTCTGTTCAATGATTTTAACCTGCTATATGTCATAGTTTTAACTTGCAGATGCACAC
AATTTTGCAATGTTTACAGAAAGCACTGAGTTAGACAAGCAATTCCTTTATTAAAAAAAA
GTACGTATTTAGATAATCATACTTCCTCTGTGAGACAGGCCATAACTGAAAACTCTTAAA
TATTTAGCAATCAAATAGGAAATGAATGTGGACTTACTAAATGACTTTAATTCTATTATA
AGAGCATATTTTAGAGTACCTATCTGCCAATTATATTTTAACATTTAAAGCAAAGTCCTC
TACTTGATTTATATTATATGTAGCTTTGCTGAGTCAAAGGAAGTATCATGCAATAGGCTT
AATTACTAAATGTCAAACCAAACTTTTCTCAAACCAGGGACTATCATCTAAGATTAATTA
CAGTAATTATTTTGCGTATACGTAACTATAAGGTTATGAATCTTGCAGATGTTAACCTTT
CCGTTGCCACAAGCAAGTACTATTATTTCTGATTTTTATAATAAGAAAATCTATGTTTAA
TCAACAGAGCCTCTCAACCAAATAACATCTCAGAGATTAAGTTATATATTAAAGCATGTA
ACATAAAAGCAAGTACATATTAGTGATATATTTAAAAACAGAGGCATCAAAATGCTTAAA
ATGTAATATTTACTAAAAAATCAGATTATGGATAATGTTTGGGATTATACTTTATTGCA
TCTTTTGTTTAATTGTATTTAGAAAGCATTGTGCAATCACTTGGGAAAAATATTAAATTA
TTAACATTGAGGTATTAATACATTTAAGCCTTTTGTTTTTAAATTTCTTTTGTTCAGAGA
TTGTTTAAAATAAATATTGGCCAAAAAAAAAAGAAGATAGAGCGACAGGCAAGAACAATA
CGTAAT
